# Chromosomal Integrity after UV Irradiation Requires FANCD2-Mediated Repair of Double Strand Breaks

**DOI:** 10.1371/journal.pgen.1005792

**Published:** 2016-01-14

**Authors:** María Belén Federico, María Belén Vallerga, Analía Radl, Natalia Soledad Paviolo, José Luis Bocco, Marina Di Giorgio, Gastón Soria, Vanesa Gottifredi

**Affiliations:** 1 Cell Cycle and Genomic Stability Laboratory, Fundación Instituto Leloir, IIBBA/ CONICET, Buenos Aires, Argentina; 2 Laboratorio de Dosimetría Biológica, Autoridad Regulatoria Nuclear, Buenos Aires, Argentina; 3 Centro de Investigaciones en Bioquímica Clínica e Inmunología/ CONICET, Universidad Nacional de Córdoba, Córdoba, Argentina; Duke University, UNITED STATES

## Abstract

Fanconi Anemia (FA) is a rare autosomal recessive disorder characterized by hypersensitivity to inter-strand crosslinks (ICLs). FANCD2, a central factor of the FA pathway, is essential for the repair of double strand breaks (DSBs) generated during fork collapse at ICLs. While lesions different from ICLs can also trigger fork collapse, the contribution of FANCD2 to the resolution of replication-coupled DSBs generated independently from ICLs is unknown. Intriguingly, FANCD2 is readily activated after UV irradiation, a DNA-damaging agent that generates predominantly intra-strand crosslinks but not ICLs. Hence, UV irradiation is an ideal tool to explore the contribution of FANCD2 to the DNA damage response triggered by DNA lesions other than ICL repair. Here we show that, in contrast to ICL-causing agents, UV radiation compromises cell survival independently from FANCD2. In agreement, FANCD2 depletion does not increase the amount of DSBs generated during the replication of UV-damaged DNA and is dispensable for UV-induced checkpoint activation. Remarkably however, FANCD2 protects UV-dependent, replication-coupled DSBs from aberrant processing by non-homologous end joining, preventing the accumulation of micronuclei and chromatid aberrations including non-homologous chromatid exchanges. Hence, while dispensable for cell survival, FANCD2 selectively safeguards chromosomal stability after UV-triggered replication stress.

## Author Summary

Here we show that irradiation with low doses of UV light causes modest accumulation of replication-coupled double strand breaks (DSBs), i.e. collapsed forks. Remarkably, the Fanconi Anemia protein FANCD2 is central to prevent the aberrant processing of UV-triggered DSBs and the generation of micronuclei and chromosome fusions but is dispensable to modulate cell death. Specifically, FANCD2 promotes homologous recombination-dependent repair of UV-triggered DSBs, thus preventing their aberrant processing by non-homologous end joining. Hence, the homologous recombination-dependent tumor suppressor function of FANCD2 is not restricted to inter-strand crosslinks but instead extends to replication-coupled DSBs that arise from a broader range of genotoxic stimuli.

## Introduction

Fanconi anemia (FA) is a rare recessive disorder characterized by increased spontaneous rearrangements of chromosomes, tumorigenesis and cell death [1,2]. Initial signs of FA include bone or skeleton defects, renal dysfunction, short stature and very frequently abnormal hyper- and hypo-pigmentation of the skin and café_au_lait spots [3]. FA is characterized by bone marrow failure and high risk of developing myeloid leukemias and squamous cell carcinomas [4]. Cells derived from FA patients are strikingly sensitive to DNA interstrand crosslinks (ICLs), i.e. cross-links between two DNA strands. Consequently, much of our current understanding of FA comes from studies that utilize ICL-causing agents, such as mitomycin C (MMC), diepoxybutane or cisplatin, as sources of DNA damage [1,2]. To date, 17 genes with described mutations in patients were defined as components of the FA pathway that are all required for ICL repair [5].

ICL removal is generally accomplished when the replication fork abuts the DNA lesion. ICL-stalled replication forks undergo a programmed collapse, which is regulated by all FA proteins [6]. Firstly, FANCD2 is loaded onto the ICL, a process that requires the FA core complex, the D2 partner FANCI and D2 monoubiquitination [7]. Indeed, FANCD2-FANCI bind preferentially to a variety of branched DNA structures formed by ICL repair intermediates [8,9]. Moreover, the crystal structure of FANCI with DNA suggests that the ID2 complex could accommodate the X-shaped DNA structures formed by replication forks that collide with ICLs [10]. Secondly, FANCD2 recruits the XESS nuclease complex (including the nucleases XPF-ERCC1 and SLX1 and the scaffold protein SLX4) and the FAN1 and SNM1A nucleases [8]. Thirdly, these enzymes co-ordinately incise the DNA 3´and 5´of the lesion, thus unhooking the ICL. Finally, FANCD2 masters the resolution of such DNA repair intermediate by coordinating the activation of translesion DNA synthesis (TLS), homologous recombination repair (HRR) and possibly Nucleotide Excision Repair (NER) [1,2]. Collectively, solid evidence demonstrates that FANCD2 is crucial to ICL repair.

Upon γIR, a source of replication-independent DSBs, ATM activates FANCD2 by phosphorylation [11]. However, FANCD2-deficient cells are only moderately sensitive to γIR and X-rays, another source of replication-independent DSBs [12–15]. In addition, FANCD2 does not play a predominant role in the repair of DSBs generated by restriction enzymes, but it is key to the resolution of ICL-dependent replication-coupled DSBs [16]. These results led to the assumption that FANCD2 is specifically required for the resolution of all replication-coupled but not direct DSBs. However, it is yet unclear whether FANCD2 resolves DSBs generated at replication forks stalled by lesions others than ICLs.

It has been shown that the activation of FANCD2 during unperturbed S phase [17] suggests that FANCD2 participates in mechanisms unrelated to DSB repair. Indeed, FANCD2 prevents the nucleolytic degradation of nascent DNA triggered by hydroxyurea (HU) or aphidicolin (APH) and promotes fork restart immediately after drug removal [18–22]. Hence, FANCD2 not only promotes DSB repair by HRR but also attenuates DSB formation by protecting persistently stalled replication forks and promoting their reactivation.

Intriguingly, FANCD2 is activated by UV irradiation, a DNA-damaging agent which rarely causes ICL accumulation [23,24] with no persistent stalling of replication forks at doses of 20 J/m^2^ or lower [25,26]. In contrast to ICL repair, the removal of UV-induced lesions does not require coordination between TLS and NER as both processes can occur independently from each other in UV-treated cells [27]. Moreover, NER efficiency is not altered in FA-defective backgrounds [28]. Importantly, FANCD2-deficient cells show normal spontaneous and UV-C-induced point mutation frequency [29] and null or very low sensitivity to UV-light [30–33]. Nonetheless, it is intriguing that the hypo/hyperpigmentation and the café_au_lait spots that characterize the FA disease are skin-associated defects. We thus reasoned that the function of FANCD2 after UV irradiation could be revealed by exploring processes that may not necessarily trigger cell death. We found that the UV irradiation of FANCD2-depleted cells with doses as low as 1.5 J/m^2^ cause a striking increase of genomic instability markers, such as aberrant chromatid exchanges and micronuclei (MN) formation. The generation of both aberrations require DSBs [34,35]. While UV irradiation is not expected to directly cause DSBs, replication-associated one-ended DSBs (also known as double strand ends–DSEs) could accumulate when elongating forks encounter UV lesions [36,37]. Our results demonstrate that FANCD2 does not majorly modulate DSB accumulation. On the contrary, FANCD2 guarantees the correct processing of replication-coupled DSBs after UV irradiation. In particular, FANCD2 promotes the recruitment of the HRR factor RAD51 to UV-damaged DNA and the resolution of replication-associated DSBs by HRR. When FANCD2 is depleted, unleashed non-homologous end joining (NHEJ) increases genomic instability after UV irradiation. Hence, FANCD2 operates beyond ICL processing, and such function might apply to all replication-coupled DSBs generated after different genotoxic insults.

## Results

### FANCD2 preserves genomic stability after UV irradiation

**Fig 1:**
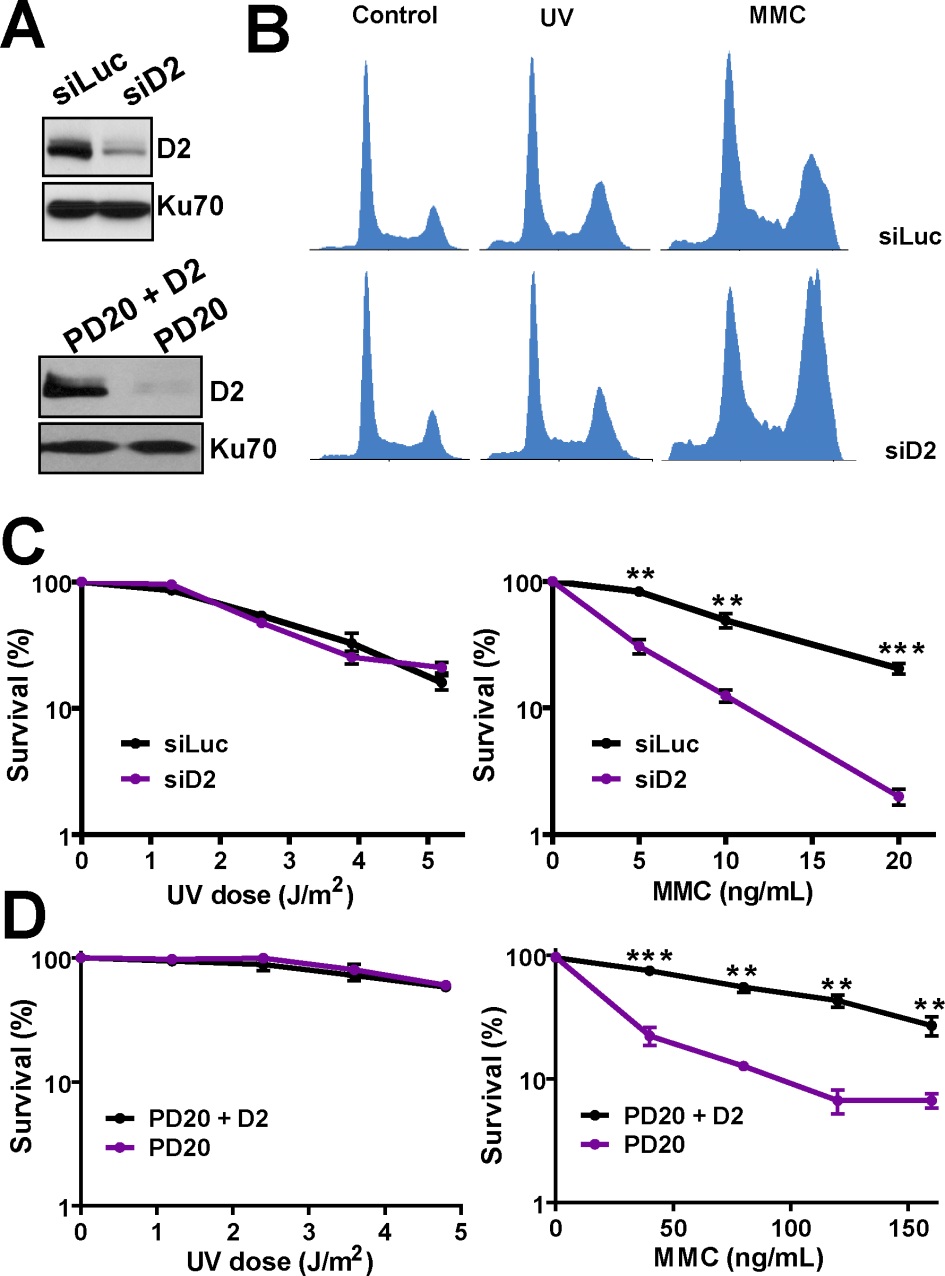
FANCD2 is activated but it is not required for cell survival after UV irradiation. A) Western blot (W.B.) of FANCD2 (D2) in U2OS and PD20 cells expressing D2 (PD20+D2). B) Flow cytometry analysis of U2OS cells transfected with control or D2 siRNA after UV irradiation (5 J/m^2^) and MMC (40 ng/ml). Samples were collected 72 hours after DNA damage induction C) Clonogenic assay in U2OS cells transfected with control and D2 siRNA and treated with the indicated doses of UV irradiation and MMC. D) Surviva (Cell titer Glo) assay in PD20 and PD20+D2 cells treated with the indicated doses of UV irradiation and MMC. In all cases, the survival rate was calculated with respect to untreated samples within the same curve. For each panel, three independent experiments were analyzed obtaining similar results. For all figures in this manuscript: significance of the differences are: *p<0.1; **p<0.01; ***p<0.001; when the p value is not shown the difference is not statistically significant. Error bars represent SEM (standard error of the mean).

As reported by others [23,38], UV irradiation induces focal organization (S1A and S1B Fig) and monoubiquitination (S1C and S1D Fig) of FANCD2, both in U2OS and PD20 cells expressing FANCD2 (PD20+D2). However, FANCD2 depletion (Fig 1A) did not alter the cell cycle distribution after UV irradiation (whereas it did alter the cell cycle profile after MMC treatment, Fig 1B). Moreover, both the transient depletion of FANCD2 in U2OS cells (Fig 1A higher panel) and the permanent loss of FANCD2 in PD20 cells obtained from patients (Fig 1A lower panel) did not affect cell survival both in short (2 days) and long (8–10 days) term assays (however we observed hypersensitivity to MMC in FANCD2-depleted samples, Fig 1C–1D). To corroborate that the doses of UV radiation used can impact the clonogenic potential of U2OS cells, we depleted Pol η (to impair TLS). We observed that TLS-Pol η-depletion reduced the colony formation ability of UV-irradiated cells (S1E Fig), therefore demonstrating that UV hypersensitivity can be revealed in our settings. The undetectable contribution of FANCD2 to UV resistance is in agreement with four previous reports that found no effect of FANCD2 depletion on cell survival after UV irradiation [30–33] and other manuscripts that showed similar results when depleting FA core proteins (see Discussion).

**Fig 2:**
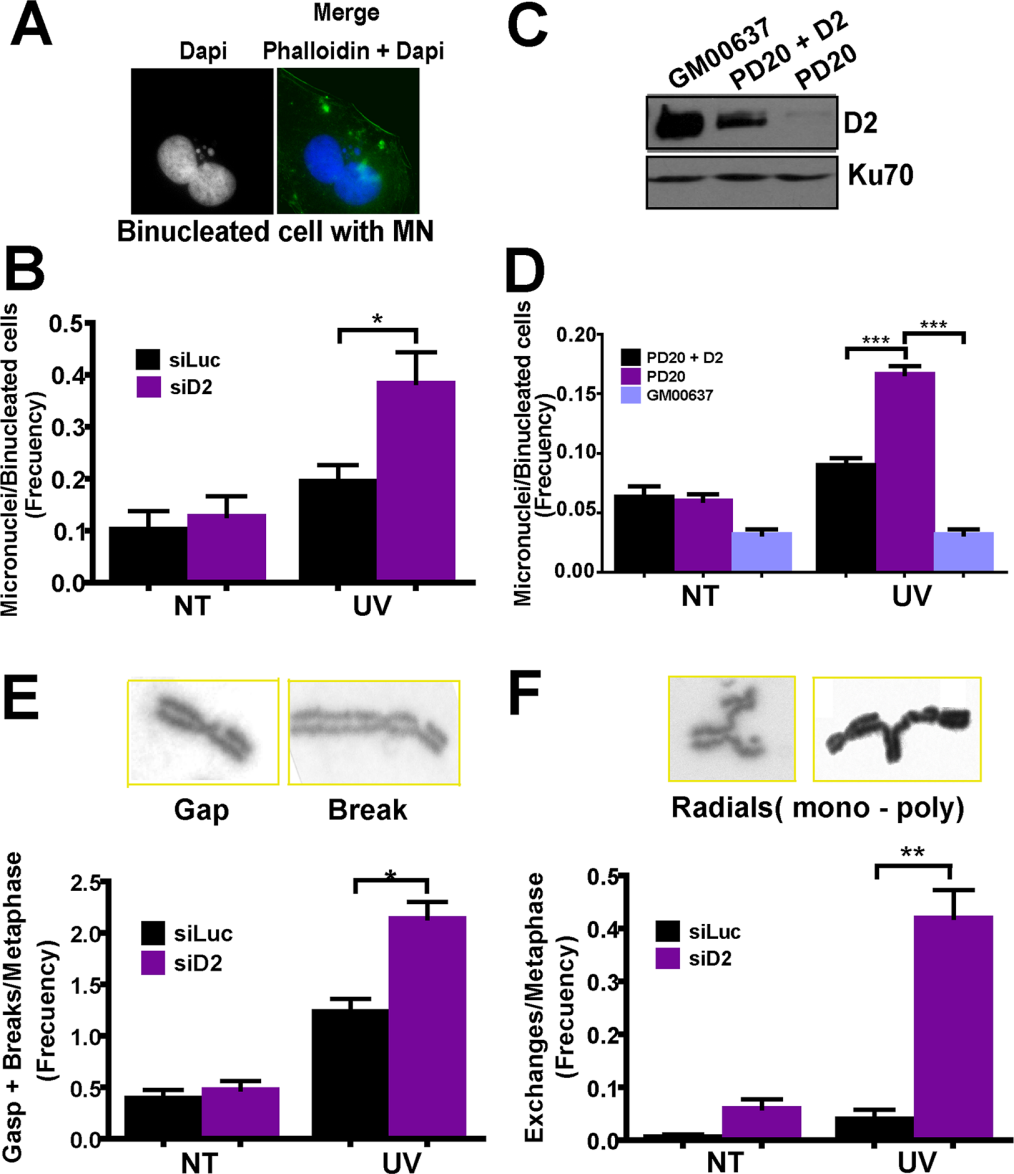
FANCD2 prevents gross chromosome rearrangements after UV irradiation. A) Representative binucleated cell with MN. B) MN accumulation in U2OS cells transfected with control and D2 siRNA after UV irradiation (5 J/m^2^). C) W.B. showing the levels of Ubi-D2 in PD20, PD20+D2 and GM00637 cells. D) MN accumulation in PD20, PD20+D2 and GM00637 cells after UV irradiation (5 J/m^2^). E) Gaps + breaks and F) complex chromatidic exchange accumulation in U2OS transfected with control and D2 siRNA after UV irradiation (1.5 J/m^2^). Three independent experiments were analyzed obtaining similar results.

We reasoned that, while not affecting cell survival, FANCD2 depletion could jeopardize the stability of the genome after UV irradiation. To evaluate this possibility, we first analyzed MN formation at the lowest dose required to achieve a detectable difference between untreated and UV-irradiated samples (5 J/m^2^). Strikingly, when depleting FANCD2, the frequency of MN increased in UV-irradiated U2OS (Fig 2A and 2B) and in PD20 cells, when compared to control GM00637 fibroblasts or PD20 reconstituted counterparts, respectively (Fig 2C and 2D). MN are formed when DSB are processed in a manner that excludes fragments of chromosomes from nuclei during/ after karyokinesis and before cytokinesis [34]. Although not widely accepted, UV irradiation has been reported as a source of DSB formation [39–41]. We therefore inferred that the increase in MN in UV-treated FANCD2-depleted cells results from an increase in the number of DSBs and/or because of aberrant DSB processing. After UV irradiation, the most likely sources of DSBs are replication-coupled, one-ended double-strand ends generated at collapsed replication forks. The deficient resolution of replication-coupled DSBs increases replication-derived chromatid aberrations, which are specifically generated in S phase [42]. Thus, we evaluated the role of FANCD2 on chromatid aberrations after UV irradiation. We first determined the lowest dose required to upregulate these aberrations in control samples (1.5 J/m^2^, Fig 2E and 2F). Interestingly, despite modest FANCD2 ubiquitination at low UV doses (S1F Fig), chromatidic breaks/gaps were upregulated in such conditions when FANCD2 was depleted (Fig 2E). Moreover, aberrations such as chromatid exchanges (mono and poly-radial chromosomes), which have been exclusively associated to replication-coupled DSBs [35,42] robustly increased in FANCD2-depleted but not in control cells (Fig 2F). Importantly, only chromatid (generated in S/G2 phase) but not chromosome (generated in G1/G0) exchanges [42] accumulated in UV-irradiated FANCD2-depleted samples (S2 Fig). Altogether, Figs 2 and S2 indicate that FANCD2 activation is required to avoid aberrant processing of replication-coupled DSBs after UV irradiation.

### FANCD2 does not modulate DSBs accumulation after UV irradiation

**Fig 3:**
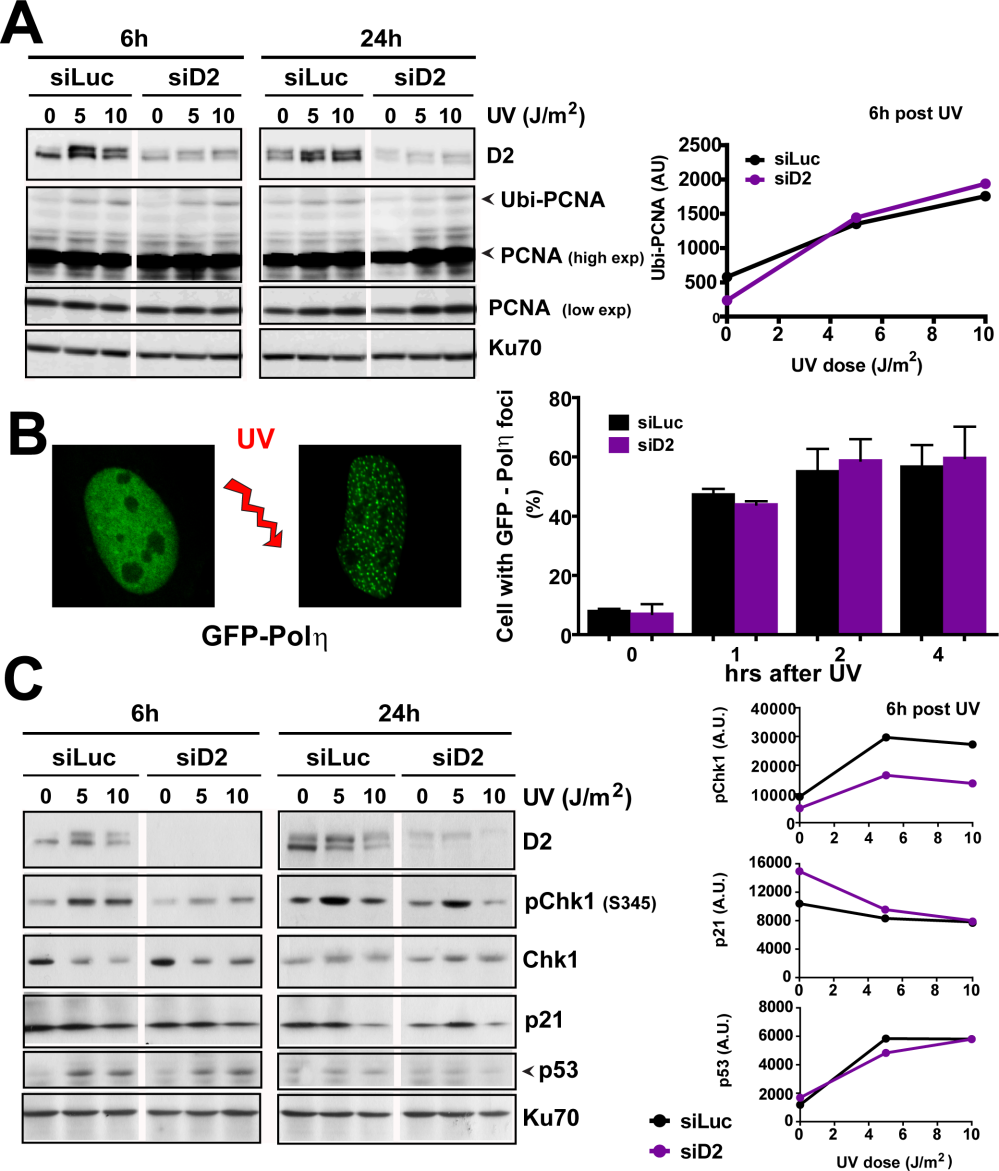
FANCD2 depletion does not modulate TLS or checkpoint markers after UV irradiation. A) W.B. showing the extent of PCNA ubiquitination in control and D2 depleted samples after the indicated doses of UV irradiation in U2OS cells. Images belong to lanes within the same gel and correspond to the same exposure. Quantification of Ubi-PCNA levels 6 hours post-UV is shown on the right. B) Percentage of U2OS cells with more than 10 GFP-Pol η foci at the indicated times after UV radiation (5 J/m^2^). C) W.B. showing phospho-Chk1 (S3545), Chk1, p53 and p21 levels in U2OS transfected with control and FANCD2 siRNAs. Images belong to lanes within the same gel and correspond to the same exposure. Quantifications of p-Chk1, p53 and p21 normalized to KU70 for the 6-hours´ time point are shown on the right. Figure is representative of 3 independent experiments for each panel.

The afore-mentioned results indicate that FANCD2 either prevents DSB formation or regulates their processing once they are formed. To explore the first possibility, we first analyzed PCNA monoubiquitination and Pol η recruitment to replication factories, two hallmarks of UV-triggered TLS, a well-characterized mechanism that aids DNA replication across UV-triggered DNA lesions and could thus prevent UV-induced DSB formation [43]. [43]. It has been previously demonstrated that FA core components [29,44] but not FANCD2 [29] promote TLS events after UV irradiation. However one report indicates that FANCD2 depletion reduces the ratio of Pol η focus formation over total Pol η signal in UV-irradiated (20 J/m^2^) Hela cells [45]. Based on these previous reports, we reasoned that the depletion of FANCD2 could modulate TLS markers in our settings. When analysing PCNA ubiquitination in FANCD2-depleted samples and PD20 cells (Figs 3A and S3A), alterations were not evident at time points (6 hours) in which TLS events are expected to be fully active [46] or at later (24hrs) time points at doses used in MN formation and induction of chromosomal aberrations (*≤5 J/m*^*2*^ *–*Figs 1 and 2*)*. To further explore a potential modulation of TLS activity after FANCD2 knockdown we also evaluated the recruitment of TLS Pol η to replication factories, which is another parameter of TLS activation [47]. Here we observed that the proportion of cells with Pol η foci was not modulated by FANCD2 depletion in our experimental settings at 5 J/m^2^ (Fig 3B). This is in agreement with the previously reported negligible contribution of FANCD2 to PCNA ubiquitination, Rev1 recruitment to replication factories and the unaltered TLS-dependent mutagenesis of UV-irradiated FANCD2-depleted samples [29,48]. Hence, two central TLS parameters were not modulated by FANCD2 knockdown at UV doses such as 5 J/m^2^, which do alter the genomic stability of FANCD2-depleted samples.

We then explored checkpoint activation, which is up-regulated by replication fork stalling and/or by increased DSBs levels. Chk1 phosphorylation is readily induced after low doses of UV irradiation [49] and increases when FA core components are depleted, possibly as a consequence of TLS defects [44]. In contrast, Chk1 phosphorylation at Ser 345 was transiently reduced 6hs -but not 24hs- post-UV in FANCD2-depleted U2OS and in PD20 cells (Figs 3C and S3B). Moreover, the extent and the timing of p53 activation and p21 downregulation after UV irradiation [50] were not modified when FANCD2 was depleted (Fig 3C). Together, these results suggest that there is no persistent reprogramming of TLS and Chk1 signals in FANCD2-depleted cells.

**Fig 4:**
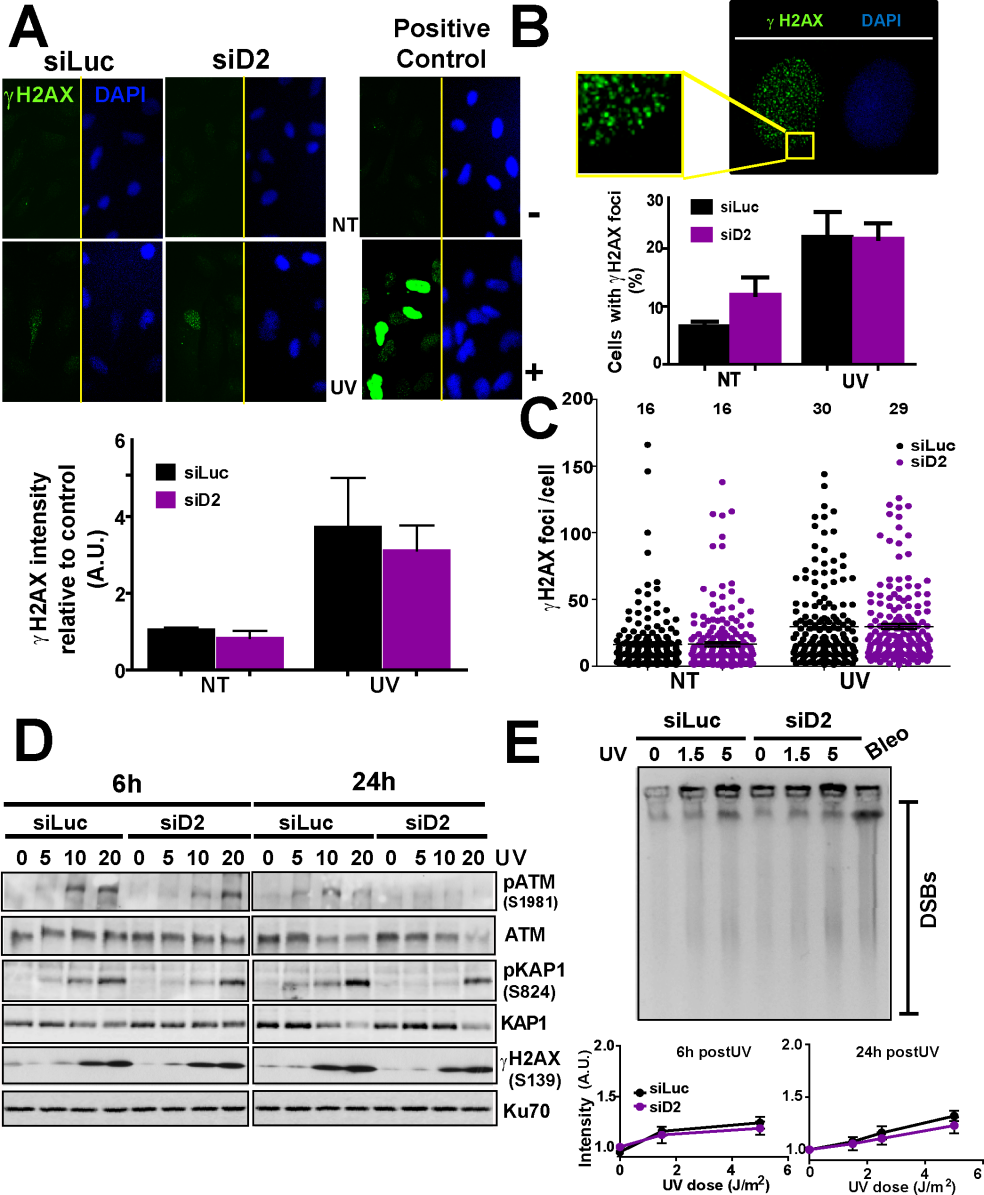
FANCD2 depletion does not increase DSB accumulation after UV irradiation. A) Representative panels showing γH2AX intensity and DAPI staining. Cisplatin treatment was used as positive control. The quantification of γH2AX intensity in 300 nuclei 24h after UV irradiation (5 J/m^2^) is shown. B) Quantification of the number of cells with γH2Ax foci; C) number of γH2AX foci/cell for the same experiment shown in A. γH2Ax intensity and foci/cell were quantified using ImageJ software. D) W.B. showing the levels of phosphorylated-ATM, γH2AX and KAP1 in U2OS cells transfected with control and D2 siRNA at the indicated time points and doses of UV irradiation. E) Pulse field gel electrophoresis showing the levels of DSB formation in U2OS transfected with control and D2 siRNA 6 hours after UV irradiation. Bleomycin treatment was used as positive control. Quantifications are shown for experiments performed at 6 and 24 hours post-UV irradiation. Figures are representative of 3 independent experiments.

We then asked whether the total number of DSBs increases in UV-irradiated samples after FANCD2 transient or permanent knockdown. Supporting the notion of a constant number of DSBs, we found that the activating phosphorylation of the histone variant H2AX (γH2AX—S139) [51] increased in a manner that depended on the UV dose but not on the levels of FANCD2 (Fig 4D). Moreover, when specifically focusing on the 5 J/m^2^ dose, the intensity of the γH2AX signal modestly increased with respect to sham-irradiated controls with no significant changes after FANCD2 depletion (Fig 4A). The percentage of cells with γH2AX foci (Figs 4B, S4A and S4C) and the number of γH2AX foci per cell (Fig 4C) were also unaffected by FANCD2 depletion. These results are opposite to those obtained when we analyzed FANCD2-depleted cells treated with the ICLs inducer, MMC (S4B and S4C Fig). Since γH2AX foci can be formed in the absence of DSBs [52] we evaluated other markers of DSBs such as the phosphorylation of ATM kinase at S1981 or of KAP1 at S824 [7,53]. p-ATM did not increase and rather decreased in FANCD2-depleted samples (U2OS in Fig 4D and PD20 cells in S3C Fig). Similarly, pKAP1 levels did not increase in UV-irradiated FANCD2-depleted samples (Figs 4D and S3C). Our results are thus in agreement with a recent report from the Vaziri group showing Tunnel negative staining of FANCD2-depleted samples [54]. Collectively, these data suggest that FANCD2 depletion does not increase the levels of DSBs both before and after UV irradiation. To confirm this hypothesis, we set up a Pulse Field Gel Electrophoresis (PFGE) analysis to directly measure DSB formation. We observed no significant differences between control and FANCD2-depleted samples both 6 and 24 hours post-UV irradiation (Fig 4E). Similar results were obtained in PD20 cells (S4C Fig). While it might be argued that PFGE might have low sensitivity to detect small amounts of DSBs, our experimental setup proved to be sensitive enough to detect DSBs even at the lowest doses of UV irradiation (Fig 4E). Moreover, as a control of our PFGE experimental setup, we confirmed that FANCD2 prevents DSB accumulation in MMC-treated (S4E Fig) but not in UV-irradiated samples (S4D Fig). Collectively, the experiments in Figs 3 and 4 and S3 and S4 suggest that the infrequent DSBs that accumulate after UV irradiation are not upregulated by FANCD2 depletion.

### FANCD2 promotes HHR at replication associated-DSBs and prevents NHEJ after UV irradiation

**Fig 5:**
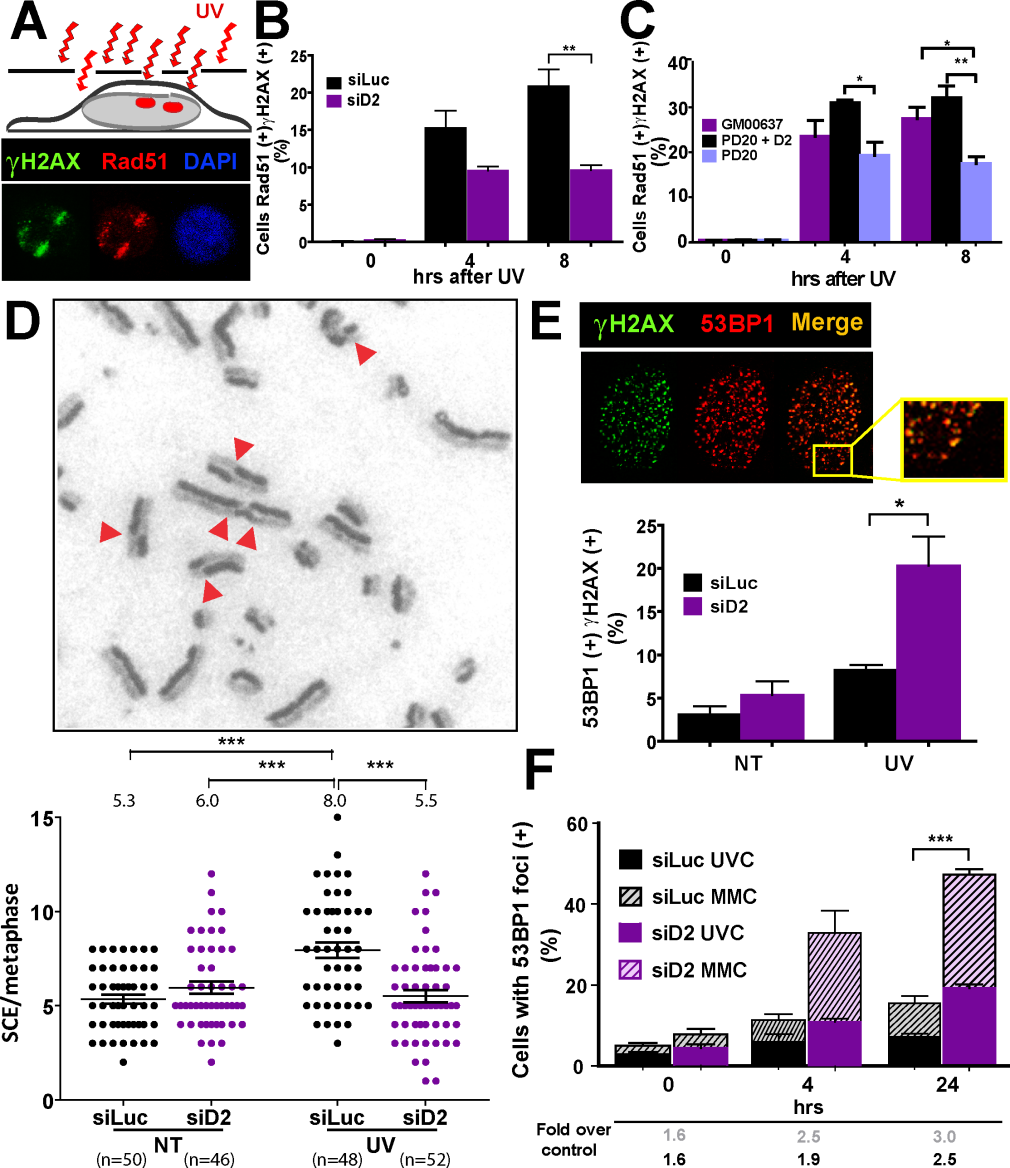
FANCD2 facilitates the recruitment of Rad51 to UV-damaged DNA and the activation of sister chromatid exchanges. A) Schematics and representative image of the recruitment of Rad51 to UV-irradiated sub-nuclear regions (visualized with γH2AX staining). B) Rad51 recruitment to damaged nuclear regions in U2OS cells transfected with control and D2 siRNAs, and C) in PD20 and PD20+D2 samples after UV irradiation (5 J/m^2^). D) Representative panel and SCE quantification in U2OS cells transfected with control and D2 siRNA (1.5 J/m^2^). E) 53BP1 and γH2AX focal organization in control and UV-treated cells (5 J/m^2^) transfected with FANCD2 or control siRNA. F) Focal organization of 53BP1 after UV irradiation (5 J/m^2^- solid color columns) and MMC treatment (40 ng/ml- striped columns) in U2OS cells transfected with control and D2 siRNA. The percentages of cells with 53BP1 foci in both UV- and MMC-treated samples are expressed as folds compared to untreated cells. Fold increases with respect to controls are shown below in black (UV) and grey (MMC). Significant differences for UV-treatment are shown (for MMC, ***p<0.001 at 24hrs). Figures are representative of 3 independent experiments.

Rad51 is a highly conserved protein that promotes homology search and strand invasion events during HRR [55]. Rad51 recruitment to chromatin is thus a hallmark of HRR activation. We therefore analyzed the local recruitment of Rad51 to unshielded regions within UV irradiated nuclei to explore the FAND2 contribution to UV-dependent HRR (Fig 5A–5C). Interestingly, transient or permanent depletion of FANCD2 in U2OS and PD20 cells impaired Rad51 recruitment to unshielded nuclear regions (Fig 5B and 5C). To evaluate the functional contribution of FANCD2 to UV-induced HRR, we explored the frequency of homologous recombination events evidenced as the exchange of large DNA regions between sister chromatids (sister chromatid exchange-SCE) [56]. The defective accumulation of SCEs indicates defects in HRR activation at replication-associated DSBs [57]. Notably, the number of SCE decreased in UV-irradiated FANCD2-depleted samples (Fig 5D). This result suggests that FANCD2 directs the processing of UV-triggered DSBs generated at collapsed forks into HRR resolution.

Cells choose to repair DSBs by HRR or NHEJ mainly depending on its replicative status [58]. Therefore, we evaluated the effect of FANCD2 depletion on the recruitment to γH2AX foci of a factor that is recruited to DSBs committed to NHEJ, the BRCT-containing protein 53BP1 [59,60]. Interestingly, the percentage of 53BP1 foci colocalizing with γH2AX foci was upregulated in UV-irradiated FANCD2-depleted samples (Fig 5E). Consistently, the total number of cells with 53BP1 foci increased in UV-treated FANCD2-depleted samples, albeit less markedly than after MMC treatment (to allow an easier comparison, the percentages of cells with 53BP1 foci in UV and MMC treated-cells are shown as overlapped bars in Fig 5F). Moreover, the cells with increased 53BP1 foci were almost exclusively those that were transiting S phase at the time of UV irradiation (S5A–S5C Fig), demonstrating that FANCD2 may prevent NHEJ events in S-phase. It is important to mention that other functions of 53BP1 such as the shielding of fragile DNA in G1 phase were recently documented [61,62]. Such 53BP1 structures are generated because of defective chromosomal segregation and are characterized by fewer but larger 53BP1 foci in G1 [61,62]. To evidence such 53BP1 foci we performed an EdU incorporation right before fixation and focused our analysis in EdU negative samples (S5D Fig). In such experimental settings, the percentage of cells with 53BP1 foci were unaffected by UV-irradiation in cells depleted from FANCD2 (S5E Fig). Hence, FANCD2 promotes the recruitment of HRR factors but not of NHEJ factors to UV-damaged DNA in cells transiting S phase.

### NHEJ generates aberrant chromosomes in FANCD2-depleted UV-irradiated cells

**Fig 6:**
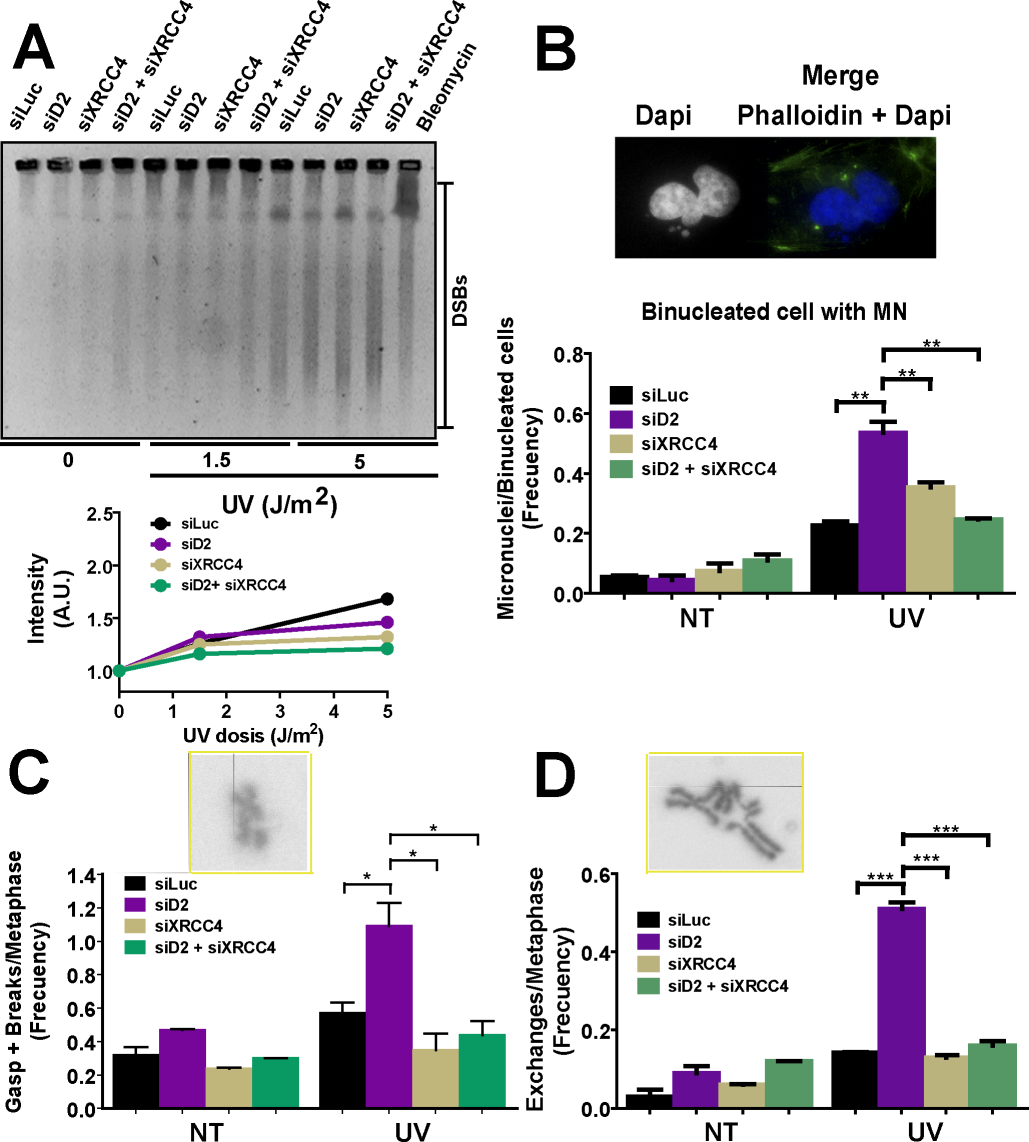
Chromosome aberrations caused by UV irradiation of FANCD2-depleted cells are completely reverted by NHEJ inactivation. A) Pulse field gel electrophoresis showing the levels of DSB formation after 24 hours of UV irradiation in U2OS transfected with the indicated siRNA. Data quantification is shown underneath the PFGE image. B) MN accumulation in binucleated cells; C) gaps and breaks and D) complex chromatidic exchanges. Two independent experiments were analyzed obtaining similar results.

To evaluate the contribution of NHEJ to the genomic instability of FANCD2-depleted cells, we transiently downregulated the NHEJ core component XRCC4 [63]. The depletion of XRCC4 in U2OS cells (S6A Fig) had no effect on the number of cells with 53BP1 foci (S6B Fig), the levels of DSBs (Figs 6A and S6C), the clonogenic potential (S6D Fig) or the accumulation of chromosomal abnormalities (Fig 6B–6D) in sham- or UV-irradiated samples. As NHEJ is a pathway that resolves replication-independent DSBs [15], this result indicates that UV is not a source of such DSBs, while UV might trigger replication-coupled DSBs. The simultaneous depletion of XRCC4 and FANCD2 did not affect DSB accumulation (Figs 6A and S6C) in comparison to FANCD2- or sham-depleted samples, thus reinforcing the notion that FANCD2 depletion does not contribute to DSB formation. Cell survival was also unaffected by simultaneous depletion of XRCC4 and FANCD2 (S6D Fig). However, the percentage of cells with 53BP1 foci increased (S6B Fig), thus suggesting a potential delay in the processing of DSB at such foci in XRCC4- and FANCD2-depleted cells. Remarkably, XRCC4 depletion rescued the accumulation of chromatid aberrations and MN formation caused by FANCD2 depletion (Fig 6B–6D). Similarly, XRCC4 depletion rescued MN accumulation in PD20 cells (S7A–S7C Fig). Importantly, the prevention of MN accumulation after UV irradiation depended predominantly on FANCD2 ubiquitination as PD20 cells expressing the FANCD2 K561R mutant (PD20+D2 KR^o^) had MN levels similar to those in PD20 cells (S7B and S7C Fig). Moreover, the increased UV-associated genomic instability of PD20 cells expressing FANCD2 K561R was also rescued by XRCC4 depletion (S7B and S7C Fig). Finally, MN accumulated primarily in EdU-positive cells (S7D and S7E Fig), i.e. cells transiting S phase at the time of UV irradiation (see timeline in S7D Fig). Altogether, these results demonstrate that FANCD2 is crucial to the repair of replication-derived DSBs generated independently from ICLs. In contrast to FANCD2 function during ICL repair, FANCD2-dependent DSB repair pathway choice after UV irradiation is irrelevant to cell survival but it is key to safeguarding genomic stability.

## Discussion

In this work we show a novel role for FANCD2 in the maintenance of genome stability in response to UV-induced DNA lesions. Moreover, our study provides strong support to previous models proposing that FANCD2 facilitates HRR-dependent resolution of DSBs generated from different sources of genotoxic stress (see model in S8 Fig).

### DSBs are formed after UV irradiation and require FANCD2 for their correct processing

It has been previously reported that the elimination of the FA pathway has a modest or null effect on UV sensitivity. In fact, with the exception of FANCM, cells deficient in FANCC, FANCA, FANCE, FANCL, FANCD2 and FANCJ are not or modestly hypersensitive to UV light ([23,30–33,44,64–67] and this work). Remarkably however, we have unveiled a function of FANCD2 after UV irradiation. In particular, we show that FANCD2 preserves genomic stability by modulating the correct processing of DSBs generated during the replication of UV-damaged DNA. Whereas DSBs are not caused directly by UV irradiation [68], the accumulation of DSBs have been previously reported after 8–10 J/m^2^ [39–41]. Indeed, we demonstrate herein that DSBs are formed at UV doses of ≤5 J/m^2^. For example, UV doses as low as 1.5 J/m^2^ induce SCE in control samples and complex aberrations (radials) in FANCD2-depleted samples. The formation of SCEs, aberrant chromatid exchanges and MN in binucleated cells require not only DSBs, but also DNA replication [69]. Therefore, UV-triggered DSBs are most likely generated as a consequence of DNA replication across damaged DNA. In fact, ATM phosphorylation after UV irradiation takes place predominantly in S phase [41]. Moreover, 53BP1 foci and MN in FANCD2-depleted cells accumulated almost exclusively in cells transiting S-phase at the time of UV irradiation (see S5 and S7 Figs). Thus, UV irradiation generates DSBs, most likely at collapsed replication forks. It should be noted that occasional ICLs, which depend on an alternative conformation of DNA that approximates pyrimidines from different strands, were also reported after UV irradiation [70–72]. In fact, when irradiating plasmidic DNA in vitro, a dose of 1000 J/m2 (260 nm) was required to accumulate ~0.07 ICLs/kbp [24]. While we cannot formally discard their contribution, it is unlikely that such a sporadic event could predominate over other types of fork collapses (at frequent UV lesions such as unrepaired cyclobutane pyrimide dimers and 6–4 photoproducts). Moreover, FANCD2 differentially contributes to the replication of UV- and MMC-damaged DNA (see next section), thus reinforcing a difference in the fork-collapsing event after both treatments.

Despite their increased genomic instability, FANCD2-depleted cells did not show increased DSB levels. In fact, PFGE did not reveal substantial changes in the accumulation of DSB in FANCD2–depleted samples. Consistently, KAP1 phosphorylation (a DSB marker) was not upregulated, and ATM and Chk1 phosphorylation were transiently downregulated. These results indicate that FANCD2 might process DNA repair intermediates at collapsed forks, generating substrates for ATM and Chk1 activation. Such speculation is supported by recent results indicating that after ICLs, DNA ends are resected into HRR-proficient substrates that promote robust ATM activation [73]. While we cannot further speculate on the signals leading to impaired ATM and Chk1 activation in UV-irradiated FANCD2-depleted samples, it is evident that in agreement with our PFGE results, the lack of upregulated phosphorylation of KAP1, ATM and Chk1 argues against a role of FANCD2 in the prevention of DSB accumulation after UV irradiation.

### Differential contribution of FANCD2 to the replication of UV- and MMC-damaged DNA

Our results indicate that the main role of FANCD2 after UV irradiation is to direct DSBs into HRR repair (Figs 2 and 6). This implies that FANCD2 may be crucial to the repair of replication-coupled DSBs that arise from sources other than ICLs. Indeed, our results suggest that the functions of FANCD2 in the cellular response to UV irradiation and ICL accumulation partially overlap. However, the responses are not equivalent. This conclusion is supported by the following observations: A) FANCD2 depletion does not trigger cell death after UV irradiation, which is strikingly different from the significant increase in cell death observed after MMC treatment in FANCD2-depleted cells. Moreover, while NHEJ deficiency either rescues or exacerbates cell death in FANCD2 deficient samples treated with ICL inducers [74–76], we revealed an insignificant effect of XRCC4 depletion in the survival of UV-irradiated FANCD2-depleted cells. B) NHEJ depletion abrogates all the chromatid aberrations caused by UV irradiation in FANCD2-depleted cells. While similar results were reported in other systems using MMC [74,75], the simultaneous elimination of FANCD2 and KU80 after MMC and cisplatin treatments in mammalian cells not only fails to abrogate, but instead further increases chromosomal instability [76]. Hence, while in response to UV- and ICL-damaged DNA FANCD2 facilitates HRR, the quality and/or quantity of DSBs may not be equivalent in both scenarios. In fact, HRR most likely takes place after the convergence of two opposite replication forks at the ICL [8]. Therefore, the HRR substrate during ICL repair may resemble a canonical double-ended DSB, which could be repaired by NHEJ without causing a massive chromosomal rearrangement. In contrast, fork collapse induced by UV irradiation may generate DSEs which, in FANCD2-depleted backgrounds, may induce gross chromosomal rearrangements when processed by NHEJ. Alternatively, different nucleases may be recruited to DSBs after UV irradiation or ICLs. In this respect, it should be mentioned that FANCD2 not only recruits nucleases to ICLs but also to DNA lesions generated by HU [21]. While the nuclease in charge of the processing of UV-triggered DSBs remains unidentified, we postulate that FANCD2 mediates the processing of collapsed forks into HHR-proficient substrates. In fact, as mentioned before, defective Chk1 activation may indicate defective processing of DNA in the absence of FANCD2.

### Chromosome protection by FANCD2

Since FA patients are not normally exposed to ICLs agents, a major concern of clinical relevance is to identify life-threatening sources of stress in FA patients. The group of K. Patel has elegantly shown that aldehydes are an endogenous source of ICLs [77] and that the enzyme Aldh2 is essential to prevent the accumulation of aldehyde-derived ICLs [78]. Tissues with low levels of Aldh2, e.g. the hematopoietic linage, rely heavily on the FA pathway to process ICLs generated from endogenous aldehydes [79,80]. Hence, endogenous ICLs represent important triggers for oncogenesis in FA patients. But whether they represent the sole trigger for genomic instability in FA patients is still unresolved. While previous studies have proposed that the contribution of FANCD2 to the resolution of DSBs might be *specifically* linked to inter-strand ICLs [12,14,16,81], our report demonstrates that replication-coupled DSBs unrelated to ICLs may require FANCD2 for their repair through the HRR pathway.

In addition, unanticipated HRR-independent functions of FANCD2 have been recently identified. Pioneer work from K. Schlacher and M. Jasin showed that FANCD2 and BRCA2/FANCD1 prevent degradation of nascent DNA in HU-treated cells [18,19]. It has also been shown that after HU, and in a core-independent manner, FANCD2 in concert with the Bloom helicase (BLM) restart stalled replication forks while suppressing origin firing [20,22]. In FANCD2 depleted samples, increased aberrant rearrangements of chromosomes were reported in [18,19] and increased frequencies of MN where reported in [20,22]. It is therefore possible that the defects in chromosomal integrity observed after UV irradiation are the indirect consequence of DSB-independent functions of FANCD2 at replicating DNA after UV irradiation. However, a number of evidences disfavour such hypothesis. First, the DSBs-independent contribution of FANCD2 after HU has been associated with persistent fork stalling [19], which is not frequent event after UV irradiation doses used in this study [25,26]. Second, the chromosomal integrity of FANCD2-depleted cells after UV is restored when NHEJ is silenced, therefore suggesting that the main function of FANCD2 is related to the processing of DSBs rather than to DNA replication events taking place prior to DSB formation. Third, the events taking place prior to DSBs processing after HU are independent of FANCD2 ubiquitination [82] whereas the UV-triggered events, which take place after DSB formation, are dependent on FANCD2 ubiquitination (see S7 Fig). Moreover, it is also reasonable to speculate that after HU treatment, the accumulation of at least some aberrations in FANCD2-depleted cells, e.g. the non-homologous exchanges [19] require the elimination of the FANCD2-mediated facilitation of DSB resolution by HRR (in addition to the disruption of FANCD2 functions at nascent DNA). Hence, while it is conceivable that during the replication of UV-damaged DNA FANCD2 participates in more than one (HRR-dependent and independent) process, results in Fig 6 demonstrate that the inhibition of NHEJ is a function of FANCD2, which must obligatorily be disrupted to generate many -if not all- the chromosome aberrations observed after UV irradiation. Remarkably, uncontrolled NHEJ at replication-coupled DSBs might also be the source of the chromosomal abnormalities reported in FANCD2-depleted samples subjected to replication-stressing agents such as HPV 16 E6/E7 expression [83], HU/APH treatments [19,21,84], PARP inhibition [85], R-loop accumulation [86], and dysregulated Pol κ recruitment to replication forks [87].

It is unclear to us why genomic stability but not cell survival is affected by FANCD2 depletion after UV irradiation. Similar results were reported after HU treatment [19]. It is possible that the DSBs generated by UV irradiation and HU are infrequent and therefore only tangentially contribute to cell death. Alternatively, while unresolved DSBs could be extremely toxic, their resolution, even when aberrant (e.g. in a FANCD2 depleted sample), may suffice to prevent cell death. Indeed, our data reveals multiple backup mechanisms that promote resolution of DSBs. Hence, when forks collapse, resolution mechanisms that promote cell survival may prevail even when genomic stability is compromised with multiple rearrangements. Our results suggest that low levels of replication-associated DSBs may be an important oncogenic factor if FANCD2 is not available to direct them into an error free pathway. FANCD2 is also required for the spontaneous levels of SCEs in uveal melanoma [88], thus we speculate that even during unperturbed replication FANCD2 regulates the pathway choice for DSBs repair. We propose a surveillance role for FANCD2 that is required to resolve replication-associated DSBs arising from any stress source and which might be relevant for the etiology of cancer in FA patients.

## Materials and Methods

### Cell culture, transfection, and UV irradiation

The following cells were used: U2OS cells (ATCC), GM00637 (Coriell Repositories), FANCD2-deficient PD20 cells (GM16633—Coriell Repositories) and two reconstituted counterparts, PD20 + D2 (GM16634—Coriell Repositories, a microcell hybrid expressing low levels of wt FANCD2) and PD20 + D2^O^ (PD20 cells expressing full-length FANCD2 cDNA), and PD20 K561R (overexpressed FANCD2 mutant with mutated K561 lysine). PD20, PD20 K561R and PD20 + D2 cells were a gift from J. Surralles (Universidad de Barcelona, Spain) and PD20 + D2^O^ from T. Huang (New York University). All cells were grown in Dulbecco’s modified Eagle’s medium (Invitrogen) supplemented with 10% fetal calf serum. Transfections were performed using Jet Prime (Polyplus). GFP-Pol η was a gift from A. Lehmann. UVC irradiation was performed using a CL-1000 ultraviolet cross-linker equipped with 254 nm tubes (UVP) or a XX-15S UV bench lamp from UVP. For local irradiation, a polycarbonate filter with 5 μm pores (Millipore # TMTP01300) was positioned in direct contact with cells, which were then treated with 100 J/m^2^ -equivalent to a much lower dose than the one reported in [89].

### siRNA sequences used In this study

siRNA duplexes (Thermo-Fisher Scientific) were the following:

siFANCD2: 5-UUGGAGGAGAUUGAUGGUCUA-3 [90],

siXRCC4: 5-AUAUGUUGGUGAACUGAGA-3 [91]

siPol η:5-CUGGUUGUGAGCAUUCGUGUA-3 has been recently described [92] and in our laboratory was designed by using the Invitrogen Block-iT RNAi Designer program validated with Dharmacon siRNA design software.

siLuc: 5-CGUACGCGGAAUACUUCGA-3 [93].

### Immunostaining and microscopy

For the immunodetection of FANCD2, Rad51, 53BP1 and γH2AX, cells were fixed in 2% paraformaldehyde (PFA)/sucrose and permeablized with 0.1% Triton X-100 in phosphate buffered saline (PBS). Well-assembled GFP- Pol η foci were quantified after fixation with ice-cold methanol followed by a 30-second incubation with ice-cold acetone as previously described by us [93]. EdU was detected following manufacturer’s instructions (Click-iT EdU kit– C10338). Blocking was performed overnight in PBS 2% donkey serum (Sigma). Coverslips were incubated for 1 h in primary antibodies: α FANCD2 (Novus), α Rad51 (Calbiochem), α γH2AX (Ser 139, Upstate), α 53BP1 (Santa Cruz). Secondary α-mouse/rabbit-conjugated Cy2/Cy3 antibodies (Jackson Immuno Research) and α -rabbit Alexa 488 (Invitrogen) were used. GFP-Pol η was detected by GFP auto-fluorescence. Nuclei were stained with DAPI (Sigma). Images were obtained with a Zeiss Axioplan confocal microscope or a Zeiss Axio Imager.A2. When quantifying GFP-pol η nuclear focal structures, cells with more than 10 foci were considered positive. When quantifying cells with Rad51 recruitment to locally irradiated areas of nuclei revealed by DAPI staining, only fields with γH2AX(+) staining were analyzed. Rad51 was *always* recruited to γH2AX(+) regions for all conditions tested. γH2AX staining was positive in 50% of the nuclei for all conditions tested. To quantify γ-H2AX intensity 100x images were analyzed with ImageJ. Approximately 30 pictures per condition were evaluated (300 cells); DAPI images were used as a pattern to define the position of nuclei on the images. The γ-H2AX intensity was determined in 300 nuclei/sample in arbitrary units, which were expressed as a fold increase with respect to the untreated control (siLuc non-irradiated).

### Protein analysis

Western blots were performed using the following antibodies: α FANCD2 (Santa Cruz Biotechnology; FI17), α Ku70 (Santa Cruz Biotechnology; A9), α PCNA (Santa Cruz Biotechnology; PC10), α phospho-(S1981)-ATM (Millipore), α ATM (GeneTex 2C1), α phospho-(S345)-Chk1 (Cell Signalling), α Chk1 (Santa Cruz Biotechnology, G4), α p21 (Santa Cruz Biotechnology, C19), α p53 (DO-1 and 1801) and α γH2AX (Upstate). α phospho (S824) KAP1 (Bethyl Laboratories), α KAP1 (Bethyl Laboratories), α Pol η (Santa Cruz Biotechnology; H-300). Incubation with secondary antibodies (Sigma) and ECL detection (Amersham GE Healthcare) were performed according to the manufacturers' instructions. Western blot images were taken with Image QuantLAS4000 (GE Healthcare), which allows capture and quantification of images within a linear range. These images were then quantified with the ImageJ software.

### Cell viability and clonogenic assay

While U20S cells can be used in clonogenic assays, PD20 cells did not resist such harsh treatment in our experimental settings. Clonogenic assays performed in U2OS cells involved an initial siRNA transfection step in 35-mm dishes, followed by replating 200 cells per 60-mm plate (2 plates per condition) and UV irradiation 24 hours later. 8–10 days later, colony formation was visualized by crystal violet staining. Colonies with more than 40 cells were scored as positive. For PD20 (and U2OS) cells, a viability kit was used at earlier time points (up to 72 hours). Transfected or PD20 and PD20 + D2 cells were plated in 96-well plates; 24 hours later cells were UV irradiated or treated with Mitomicyn C (MMC, Roche). When using MMC, treatment was interrupted 15 hrs later and samples were washed and incubated with fresh growing medium. The analysis was performed at the indicated hours after release. PD20 cells were subjected to the Cell Viability Assay following manufacturer’s instructions (CellTiter-Glo Luminescent Cell Viability Assay G-7570, Promega).

### Cell cycle analysis

Cells were fixed with ice-cold ethanol and resuspended in PBS containing RNase I (100 mg/ml, Sigma) and propidium iodide (50 mg/ml, Sigma). Samples were subjected to fluorescence activated cell sorting (FACS, Calibur, Becton Dickinson), and data was analyzed using the Summit 4.3 software (DAKO Cytomation).

### MN assay

U2OS and PD20 cells were plated at low density, UV irradiated 24 hours later and incubated with cytochalasin B (4.5 ug/ml, Sigma) for 40 h (U2OS) and 24 hrs (PD20). Cells were washed 1 min with hypotonic buffer (KCl 0.0075 M), twice with PBS and fixed with paraformaldehyde (PFA)/sucrose 2% for 20 min. Phalloidin and DAPI staining served to visualize whole cells and nuclei respectively. 300 binucleated cells were analyzed and the frequency was calculated as MN/binucleated cells.

### Chromosomal aberration analysis

Metaphase chromosome spreads were generated introducing minor modifications to protocols previously used by us [94]. Briefly, U2OS transfected cells were replated and UV irradiated (1.5 J/m^2^). Before harvesting, cells were treated with Colcemid (0.08 μg/ml, KaryoMAX, Invitrogen) for 20 h. Cell pellets were incubated in hypotonic buffer (KCl 0.0075 M) at 37°C for 4 min, followed by fixation in Carnoy’s fixative (3:1 methanol:glacial acetic acid). Cells were dropped onto slides and air-dried before staining with 6% w/v Giemsa in Sorensen’s buffer (2:1 67 mM KH_2_PO_4_:67 mM Na_2_HPO_4_, pH 6.8) for 2 min. Samples were analyzed in an Applied Imaging Cytovision 3.7. 50 metaphase spreads were used to quantify chromosomal gaps, breaks and exchanges. This protocol was set up to enrich samples with cells transiting the first cell cycle after UV irradiation.

### Sister chromatid exchange analysis

Transfected U2OS cells (with siLuc and siD2) were UV irradiated (1.5 J/m^2^). To generate the differential staining of sister chromatids, cells were incubated with the thymine analogue 5-bromo-2´-deoxyuridine (BrdU, 20 μM, Becton Dickinson) for two complete cell cycles. Colcemid (0.08 μg/ml, KaryoMAX, Invitrogen) was added 20 h before harvest. Metaphase chromosome spreads were prepared as mentioned above (see Chromosomal aberration analysis). Slides were air dried for 5 days, stained with Hoechst (5 μg/ml, Invitrogen), irradiated with a sun lamp (Ultra-Vitalux, OSRAM) for 7 min and finally stained with 6% w/v Giemsa in Sorensen’s buffer for 2 min. The treatment with Hoechst dye and Giemsa allows the newly synthesized DNA within a chromatid to be recognized, since BrdU incorporation results in much weaker staining. Sister-chromatid exchanges (SCE) were scored analysing chromosomes in 50 metaphase spreads.

### Pulsed-field gel electrophoresis

To prepare agarose plugs we used the protocol reported in [52] with minor modifications. Briefly, samples were UV irradiated, 6 or 24 h later 1 x 10^5^ cells were melted into 1.0% Pulsed Field Certified Agarose (Bio-Rad Laboratories). Agarose plugs were digested in 0.5 M EDTA-1% N-laurylsarcosyl-proteinase K (1 mg/ml, Invitrogen) at 50°C for 48 h and washed four times in TE buffer and loaded onto a separation gel (1.0% Pulsed Field Certified Agarose). Electrophoresis was performed on CHEF DR II equipment (Bio-Rad Laboratories) as previously described in [52]. A second electrophoresis protocol was also used [49], with minor modifications: 9 h, 120°, 5.5 V/cm, 30–18 s switch time; 6 h, 120°, 4.5 V/cm, 18–9 s switch time; 6 h, 120°, 4 V/cm, 9–5 s switch time, for 24 hr. A 2h-bleomycin (100 μg/mL, Gador) treatment was used as a positive control. Ethidium bromide–stained gels were visualized in a White Ultraviolet Transilluminator (UVP) or with Image Quant LAS4000, which allows capture and quantification of images within a linear range. PFGE images were then quantified with the ImageJ software.

### Quantitative real-time PCR

Cells were lysed and total RNA was extracted using Trizol Reagent (Invitrogen). 1 μg of total RNA was used as template for cDNA synthesis using ImProm-II Reverse Transcription System (Promega) and oligo-dT. Quantitative real-time PCR was performed in a MX3005P qPCR instrument (Stratagene) with Taq DNA polymerase (Invitrogen) and SyberGreen and ROX as reference dyes (Invitrogen). All amplification reactions approached 100% efficiency as determined by standard curves. Three independent biological samples were analyzed and one representative set of results is shown.

Primers used for Quantitative Real Time PCR analysis:

XRCC4 (f) 5′-AAGATGTCTCATTCAGACTTG-3′

(r) 5′ CCGCTTATAAAGATCAGTCTC-3′ [95].

GADPH: (f) 5’-AGCCTCCCGCTTCGCTCTCT-3’

(r) 5’-GAGCGATGTGGCTCGGCTGG-3’. [96]

### Statistical analysis

GraphPad Prism 5 software was used to analyse SCE, for cytogenetic experiments and foci formation experiments we used the Student's *t* test. Other calculations and graphics were performed by using Microsoft Excel 2010.

## Supporting Information

S1 Fig FANCD2 is not required for cell survival after UV irradiation. A) U2OS cells with more than 10 FANCD2 (D2) foci were quantified at the indicated time points after UV irradiation (5 J/m^2^) and B) after the indicated UV doses (4 hours post-UV). C) Western blot (W.B.) revealing levels of D2 and Ubi-D2 in U2OS and PD20 cells expressing D2 at the indicated time points after UV irradiation (5 J/m^2^). The ratio monoubi-D2/D2 is reported below each lane. D) Dose curve of UV irradiation; W.B. analysis of FANCD2 monoubiquitination. The ratio monoubi-D2/D2 is reported below each lane. E) Clonogenic assay in U2OS cell line, transfected with control, FANCD2 and pol η siRNA treated with the indicated doses of UV irradiation. Figures are representative of three independent experiments. F) Western blot (W.B.) revealing levels of D2 and Ubi-D2 in U2OS and PD20 cells expressing D2 at the indicated UV dose (5 J/m^2^). The ratio monoubi-D2/D2 is reported below each lane.
(TIF)

S2 Fig Massive chromosomal rearrangements generated after FANCD2 depletion are formed in replicating cells. A) Chromatidic and chromosomal exchanges in U2OS cells treated with control and D2 siRNA after UV irradiated (1.5 J/m^2^). Two independent experiments were analyzed obtaining similar results. B) Schematics of the aberrant rearrangements that lead to chromosomal and chromatidic exchanges.
(TIF)

S3 Fig TLS, Checkpoint and DSB markers are not upregulated in PD20 cells at low UV doses. W.B. analysis of samples obtained from PD20 and PD20+D2 revealing the levels of A) ubiquitinated PCNA and PCNA, B) phospho-Chk1 and Chk1 and C) phospho-ATM (S1981), ATM, phospho-KAP1(S824) and KAP1 in PD20 and PD20 cells reconstituted with FANCD2 (PD20+D2) at the indicated time points and doses of UV irradiation. Quantifications of the Ubi-PCNA, p-Chk1, pATM and p-KAP1 levels normalized to the control Ku70 protein, at 6 hour post UV, are shown on the right side.
(TIF)

S4 Fig FANCD2 depletion increases DSB accumulation after MMC but not after UV treatment. Quantification of the number of cells with H2AX foci after UV (A) and MMC treatment (B) in PD20 and PD20+D2 cells. C) Representative panels of experiments quantified in A and B showing H2AX intensity and DAPI staining. C) Pulse field gel electrophoresis showing the accumulation of DSBs in the indicated cell lines at 24 hours after UV irradiation. Bleomycin (Bleo) treatment was used as positive control. D) PFGE showing the accumulation of DSBs in the indicated cells at 24 hours after UV treatment. Bleomycin (Bleo) treatment was used as positive control. E) PFGE showing the accumulation of DSBs in the indicated cell lines at 24 hours after MMC treatment. Bleomycin (Bleo) treatment was used as positive control.
(TIF)

S5 Fig The increased 53BP1 foci detected after UV irradiation of FANCD2 depleted cells occur in S phase and colocalizes with γH2AX foci. A) Time line of the experiment quantified in panels B and C. U2OS cells transfected with control and D2 siRNA were UV irradiated (5 J/m^2^) and incubated with EdU (10μM) for 30 minutes immediately after UV irradiation. B) Representative microphotography (left), percentages (middle panel)) and foci number/cell (right) of 53BP1 foci in EdU (+) cells. Nuclei containing more than ten 53BP1 foci were scored as positive when calculating percentage of 53BP1 positive cells. C) Representative microphotography (left), percentages (middle panel), and foci number/cell (right) of 53BP1 foci in the EdU (-) cells from the protocol described in A. Quantifications were performed as described in B. In B) and C) a representative 53BP1 positive (green)/EdU positive (red) or negative nucleus is shown with zoom in the indicated area, highlighting a 53BP1 distribution characteristic of cells transiting/arrested in S phase at the time of fixation. D) Time line of the experiment quantified in panel E. U2OS cells transfected with control and FANCD2 siRNA were UV irradiated (5 J/m^2^) and incubated with EdU (10μM) for the last 10 minutes before fixation. E) Representative microphotography (left), percentages (middle panel), and number (right) of 53BP1 foci in EdU (-) cells. Nuclei containing more than one 53BP1 foci were scored as positive when calculating percentage of 53BP1 positive cells.
(TIF)

S6 Fig 53BP1 recruitment to damaged DNA is not reverted when NHEJ is inhibited in FANCD2 depleted samples. A) Quantitative real-time RT-PCR of XRCC4 was performed in U2OS cells transfected with the indicated siRNAs. Samples were normalized using GAPDH primers. B) 53BP1 focal organization in U2OS cells transfected the indicated siRNAs and UV irradiated with 5 J/m^2^. Figures are representative of 3 independent experiments. C) Pulse field gel electrophoresis showing the levels of DSB formation after 6 hours of UV irradiation in U2OS transfected with the indicated siRNA. D) Clonogenic assay was evaluated in U2OS transfected with the indicated siRNA.
(TIF)

S7 Fig The ubiquitination of FANCD2 prevents MN accumulation in cells that were transiting S phase at the time of UV irradiation. A) W.B. showing FANCD2 levels in PD20 cells and in PD20 cells complemented with FANCD2 (PD20 +D2), overexpressing FANCD2 (PD20+D2^O^) and the K561R FANCD2 mutant (PD20 +D2 K561R^O^). B) Quantitative real-time RT-PCR of XRCC4 was performed in PD20 cells lines described in A. Samples were normalized using GAPDH primers. C) MN accumulation in PD20 cells lines transfected with control and XRCC4 siRNA and UV irradiated (5 J/m^2^). D) Time line depicting the protocol followed to identify binucleated cells which were transiting S phase at the time of UV irradiation. E) Frequency of MN accumulation in Edu (+) and Edu (-) PD20 cells after UV (5 J/m^2^) irradiation.
(TIF)

S8 Fig FANCD2 is required downstream of DSB formation after UV irradiation in order to protect chromosome integrity. A) The interplay between TLS, NER and HRR at inter- and intra-strand crosslinks. A1) It has been extensively reported in the literature that the FA pathway coordinates the onset of TLS, NER and HRR at ICLs. A2) It has also been reported that TLS, NER and HRR are activated after UV irradiation, albeit in this case, these are independent (not coordinated) events. B) The role of FANCD2 activation after UV irradiation. B1) When UV-triggered DNA lesions are encountered by replication forks TLS aids DNA replication by tolerating the UV-lesion. Checkpoint signals also assists DNA elongation by preventing the collapse of replication forks and promoting the TLS-dependent bypass of DNA lesions [93,97]. B2) However, a fraction of the replication forks that encounter DNA lesions are permanently/irreversibly stalled; such structures may collapse creating one-ended DSBs. B3) FANCD2 (and possibly other components of the canonical FA pathway) promotes HRR at such collapsed forks. B4) The depletion of FANCD2 disfavours the HRR-mediated resolution of replication-associated DSBs after UV irradiation and promotes NHEJ activation, thus jeopardizing the integrity of chromosomes.
(TIF)
