# Correction: Chromosomal Integrity after UV Irradiation Requires FANCD2-Mediated Repair of Double Strand Breaks

**DOI:** 10.1371/journal.pgen.1011094

**Published:** 2023-12-20

**Authors:** María Belén Federico, María Belén Vallerga, Analía Radl, Natalia Soledad Paviolo, José Luis Bocco, Marina Di Giorgio, Gastón Soria, Vanesa Gottifredi

In Fig 3B of this article [1], the representative image of a GFP-Pol η nucleus before UV irradiation is incorrect as it shows a GFP-Pol ί nucleus. This image was previously published in Fig 4A of [2]. An updated version of Fig 3 is provided with this notice in which the incorrect image has been replaced by with an image showing a GFP-Pol η nucleus pre-UV irradiation. Additional GFP-Pol η nucleus images are provided in S1 File, and quantitative data underlying Fig 3B are provided in S2 File.

In S4C Fig in [1], the panels for γH2AX and DAPI in PD20 cells without UV irradiation (NT) are incorrect as they are inadvertent partial duplications of the corresponding panels for PD20 + D2 cells. An updated version of S4C Fig is provided as S3 File. This issue does not affect the quantification presented in S4B Fig. The quantitative data underlying S4A–S4B Fig is provided in S4 File of this notice.

In addition, the primary data underlying results in this article were not included with the published article, although the Data Availability Statement for this article stated, “All relevant data are within the paper and its Supporting Information files.” With this Correction, the authors provide the original raw data in S4 File and uncropped Western blot images in S5 File.

The authors apologize for the errors in the published article.

**Fig 3:**
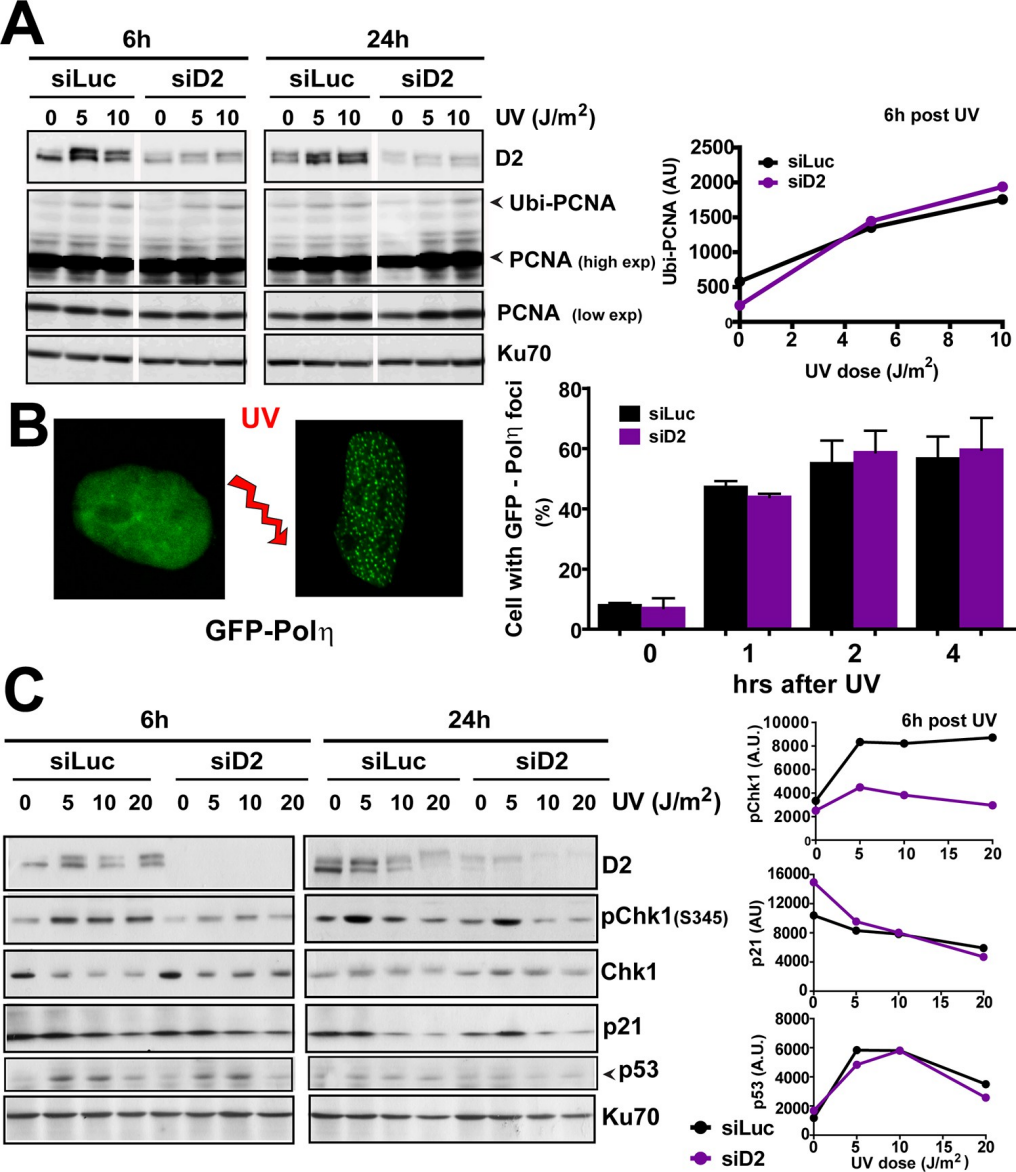
FANCD2 depletion does not modulate TLS or checkpoint markers after UV irradiation. A) W.B. showing the extent of PCNA ubiquitination in control and D2 depleted samples after the indicated doses of UV irradiation in U2OS cells. Images belong to lanes within the same gel and correspond to the same exposure. Quantification of Ubi-PCNA levels 6 hours post-UV is shown on the right. B) Percentage of U2OS cells with more than 10 GFP-Pol η foci at the indicated times after UV radiation (5 J/m^2^). C) W.B. showing phospho-Chk1 (S3545), Chk1, p53 and p21 levels in U2OS transfected with control and FANCD2 siRNAs. Images belong to lanes within the same gel and correspond to the same exposure. Quantifications of p-Chk1, p53 and p21 normalized to KU70 for the 6-hours´ time point are shown on the right. Figure is representative of 3 independent experiments for each panel.

## Supporting information

S1 File Representative GFP-Pol η and GFP-Pol ί nuclei pre- and post-UV irradiation. (TIF)

S2 File Quantitative data underlying Fig 3B. Data provided in Excel and GraphPad format.
(ZIP)

S3 File Corrected S4 Fig from [1]. γH2AX and DAPI panels in PD20 cells without UV irradiation (NT) have been replaced.
(TIF)

S4 File Raw quantitative data underlying the results in [1]. (XLSX)

S5 File Original Western blot images. (A) Annotated original Western blot images, and (B) individual original Western blot images.
(ZIP)
