# Supplementary figures and images for: Chromosomal Integrity after UV Irradiation Requires FANCD2-Mediated Repair of Double Strand Breaks
Source: PLoS Genet. 2016 Jan 14;12(1):e1005792. doi: 10.1371/journal.pgen.1005792 (PMC4712966; doi:10.1371/journal.pgen.1005792)

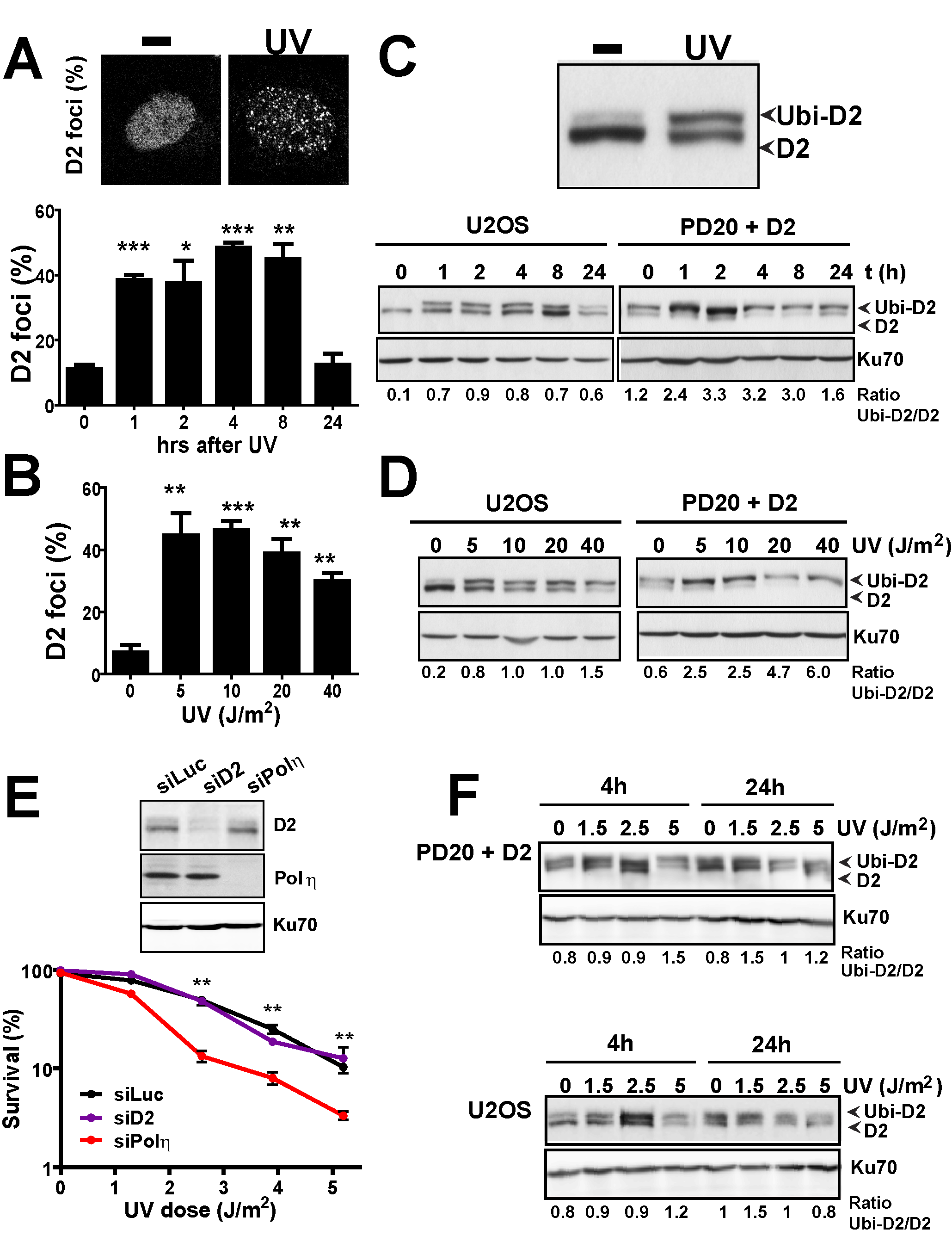

Supplement: S1 Fig — A) U2OS cells with more than 10 FANCD2 (D2) foci were quantified at the indicated time points after UV irradiation (5 J/m2) and B) after the indicated UV doses (4 hours post-UV). C) Western blot (W.B.) revealing levels of D2 and Ubi-D2 in U2OS and PD20 cells expressing D2 at the indicated time points after UV irradiation (5 J/m2). The ratio monoubi-D2/D2 is reported below each lane. D) Dose curve of UV irradiation; W.B. analysis of FANCD2 monoubiquitination. The ratio monoubi-D2/D2 is reported below each lane. E) Clonogenic assay in U2OS cell line, transfected with control, FANCD2 and pol η siRNA treated with the indicated doses of UV irradiation. Figures are representative of three independent experiments. F) Western blot (W.B.) revealing levels of D2 and Ubi-D2 in U2OS and PD20 cells expressing D2 at the indicated UV dose (5 J/m2). The ratio monoubi-D2/D2 is reported below each lane. (TIF) [file pgen.1005792.s001.tif]

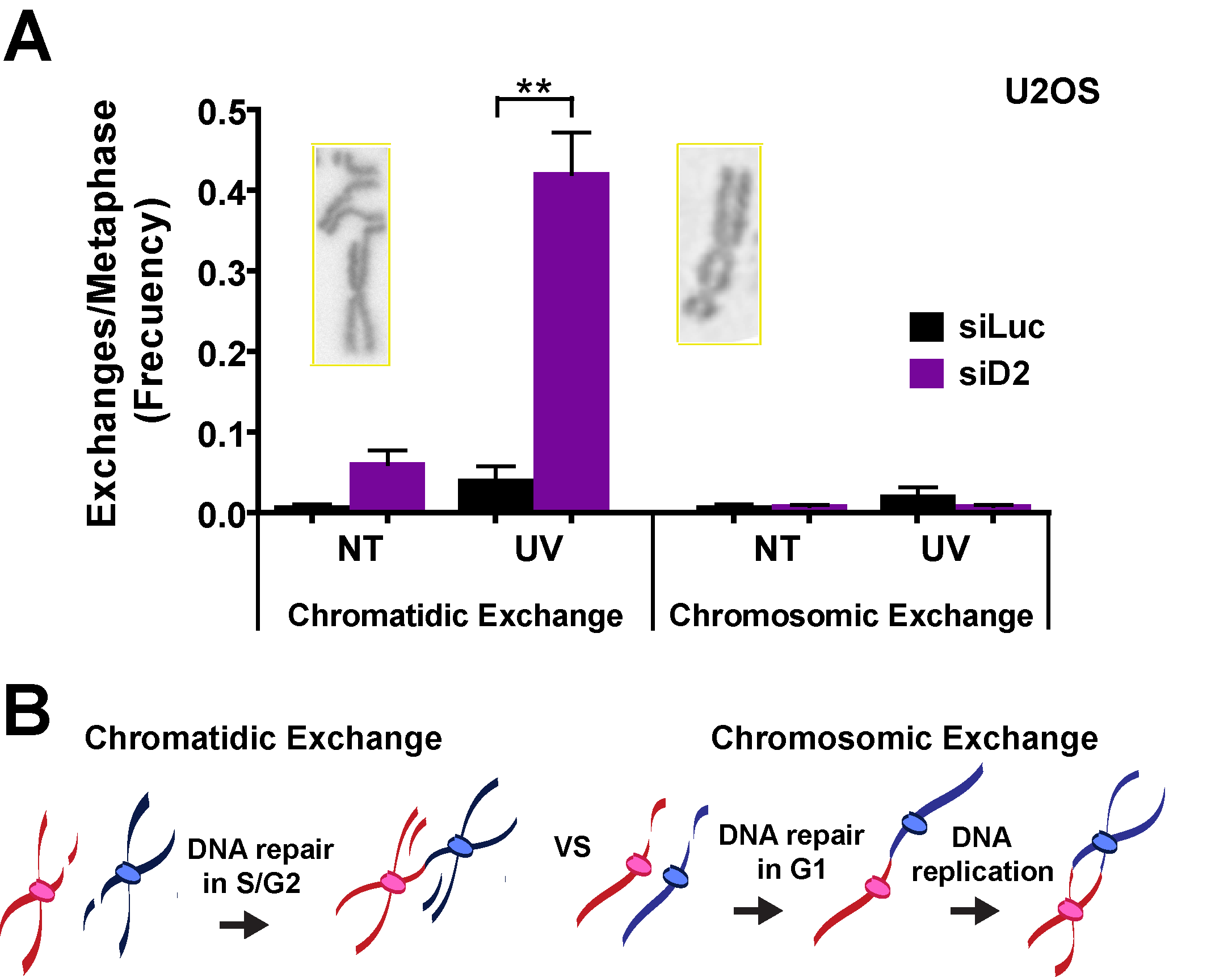

Supplement: S2 Fig — A) Chromatidic and chromosomal exchanges in U2OS cells treated with control and D2 siRNA after UV irradiated (1.5 J/m2). Two independent experiments were analyzed obtaining similar results. B) Schematics of the aberrant rearrangements that lead to chromosomal and chromatidic exchanges. (TIF) [file pgen.1005792.s002.tif]

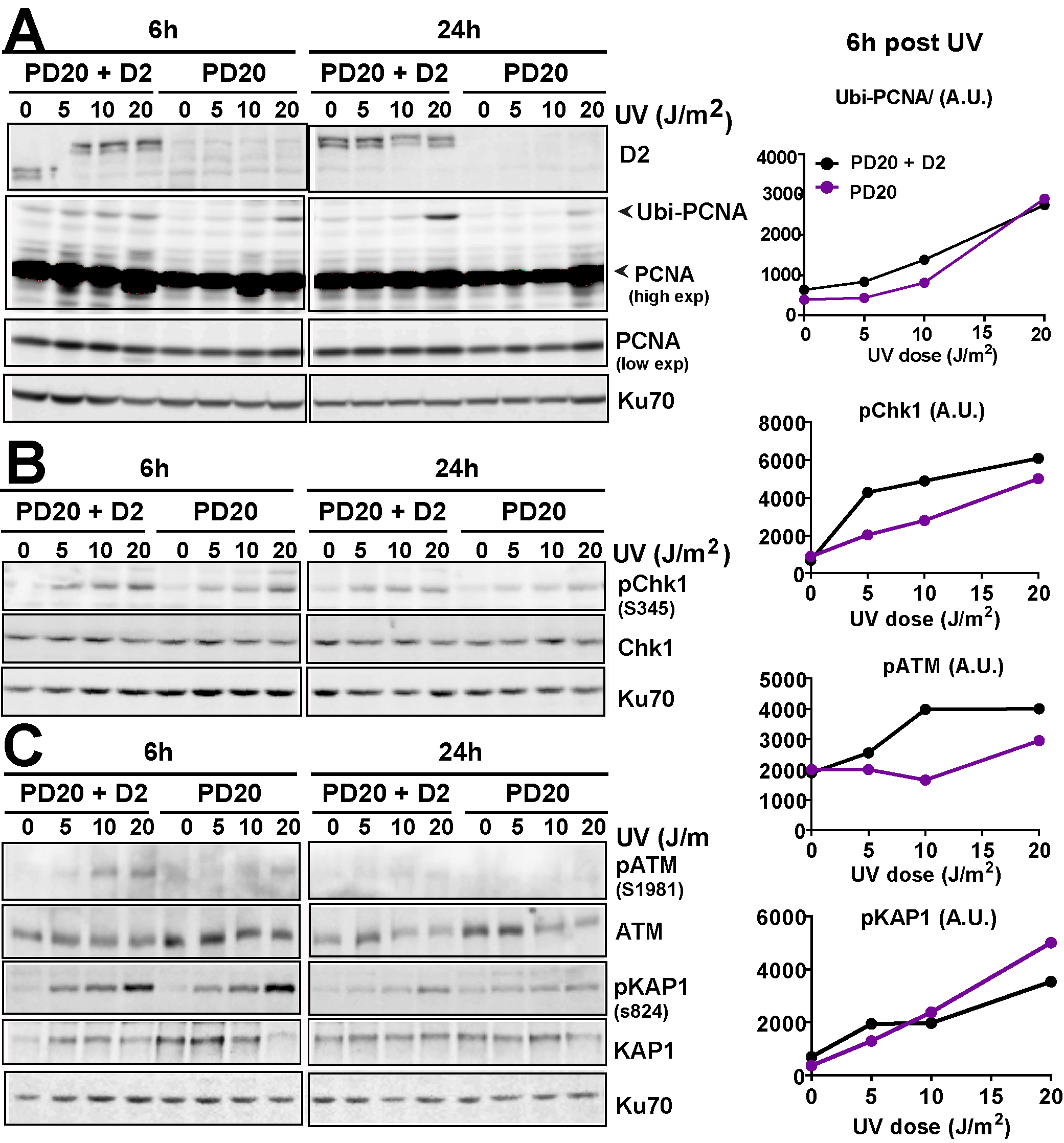

Supplement: S3 Fig — W.B. analysis of samples obtained from PD20 and PD20+D2 revealing the levels of A) ubiquitinated PCNA and PCNA, B) phospho-Chk1 and Chk1 and C) phospho-ATM (S1981), ATM, phospho-KAP1(S824) and KAP1 in PD20 and PD20 cells reconstituted with FANCD2 (PD20+D2) at the indicated time points and doses of UV irradiation. Quantifications of the Ubi-PCNA, p-Chk1, pATM and p-KAP1 levels normalized to the control Ku70 protein, at 6 hour post UV, are shown on the right side. (TIF) [file pgen.1005792.s003.tif]

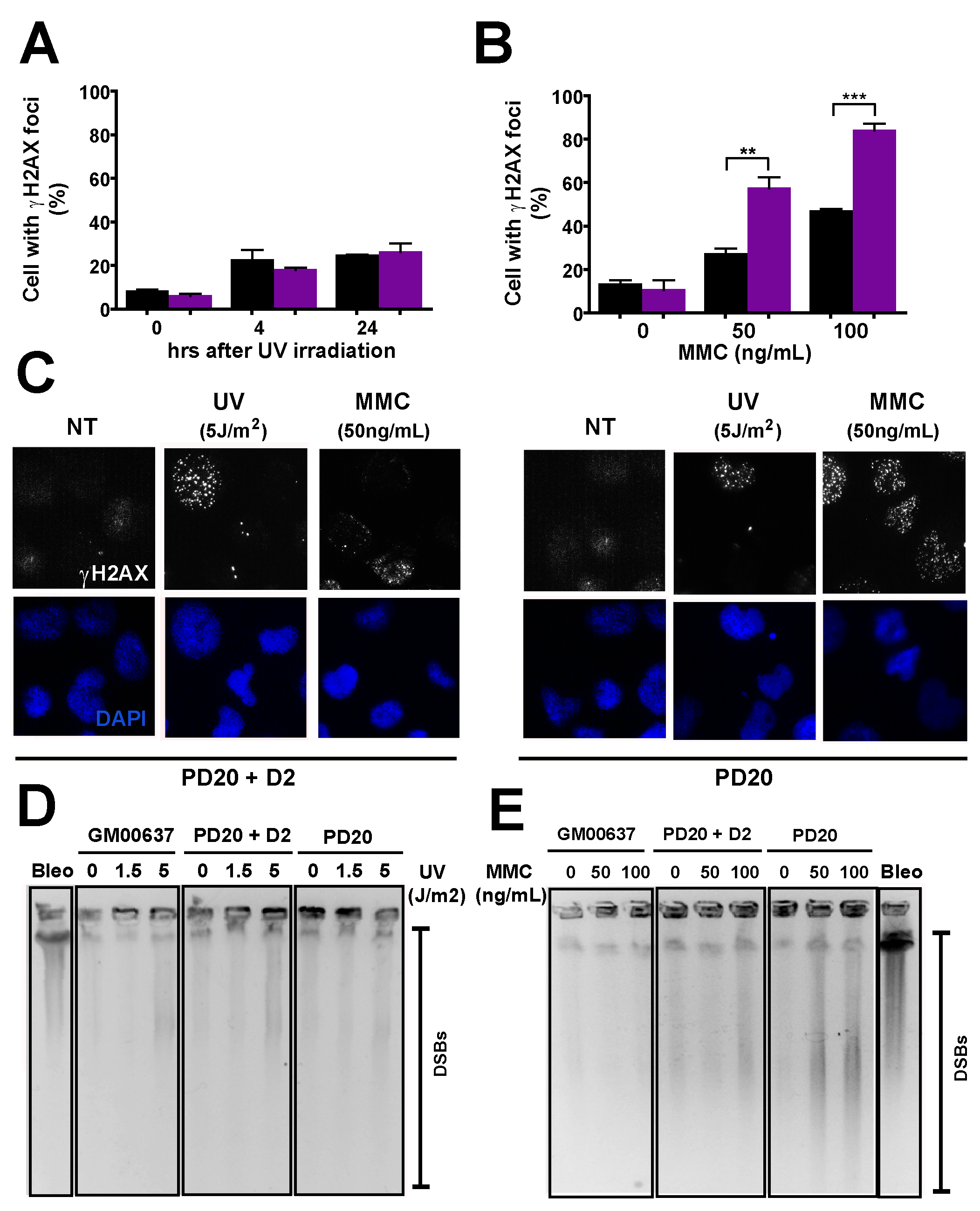

Supplement: S4 Fig — Quantification of the number of cells with H2AX foci after UV (A) and MMC treatment (B) in PD20 and PD20+D2 cells. C) Representative panels of experiments quantified in A and B showing H2AX intensity and DAPI staining. C) Pulse field gel electrophoresis showing the accumulation of DSBs in the indicated cell lines at 24 hours after UV irradiation. Bleomycin (Bleo) treatment was used as positive control. D) PFGE showing the accumulation of DSBs in the indicated cells at 24 hours after UV treatment. Bleomycin (Bleo) treatment was used as positive control. E) PFGE showing the accumulation of DSBs in the indicated cell lines at 24 hours after MMC treatment. Bleomycin (Bleo) treatment was used as positive control. (TIF) [file pgen.1005792.s004.tif]

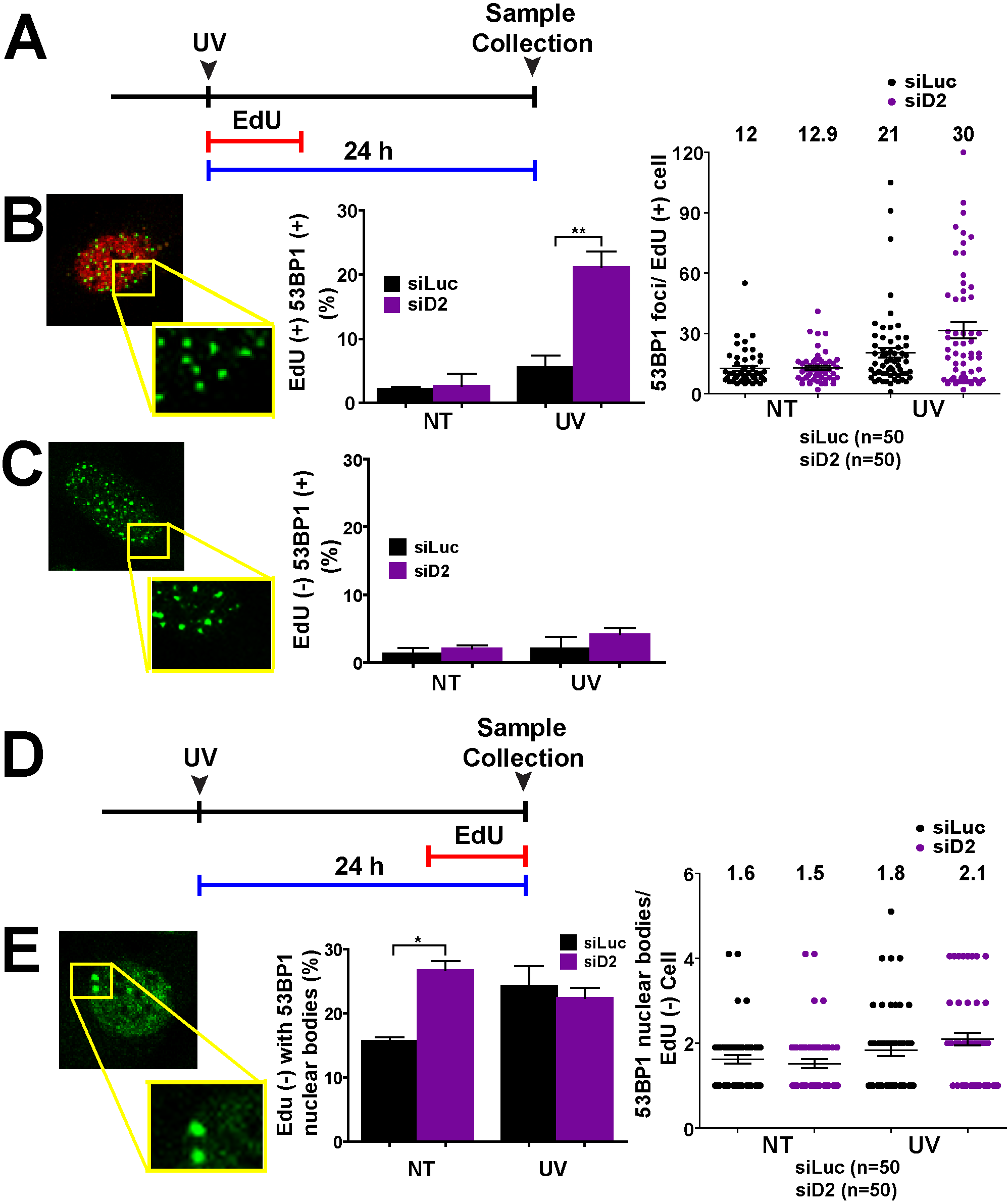

Supplement: S5 Fig — A) Time line of the experiment quantified in panels B and C. U2OS cells transfected with control and D2 siRNA were UV irradiated (5 J/m2) and incubated with EdU (10μM) for 30 minutes immediately after UV irradiation. B) Representative microphotography (left), percentages (middle panel)) and foci number/cell (right) of 53BP1 foci in EdU (+) cells. Nuclei containing more than ten 53BP1 foci were scored as positive when calculating percentage of 53BP1 positive cells. C) Representative microphotography (left), percentages (middle panel), and foci number/cell (right) of 53BP1 foci in the EdU (-) cells from the protocol described in A. Quantifications were performed as described in B. In B) and C) a representative 53BP1 positive (green)/EdU positive (red) or negative nucleus is shown with zoom in the indicated area, highlighting a 53BP1 distribution characteristic of cells transiting/arrested in S phase at the time of fixation. D) Time line of the experiment quantified in panel E. U2OS cells transfected with control and FANCD2 siRNA were UV irradiated (5 J/m2) and incubated with EdU (10μM) for the last 10 minutes before fixation. E) Representative microphotography (left), percentages (middle panel), and number (right) of 53BP1 foci in EdU (-) cells. Nuclei containing more than one 53BP1 foci were scored as positive when calculating percentage of 53BP1 positive cells. (TIF) [file pgen.1005792.s005.tif]

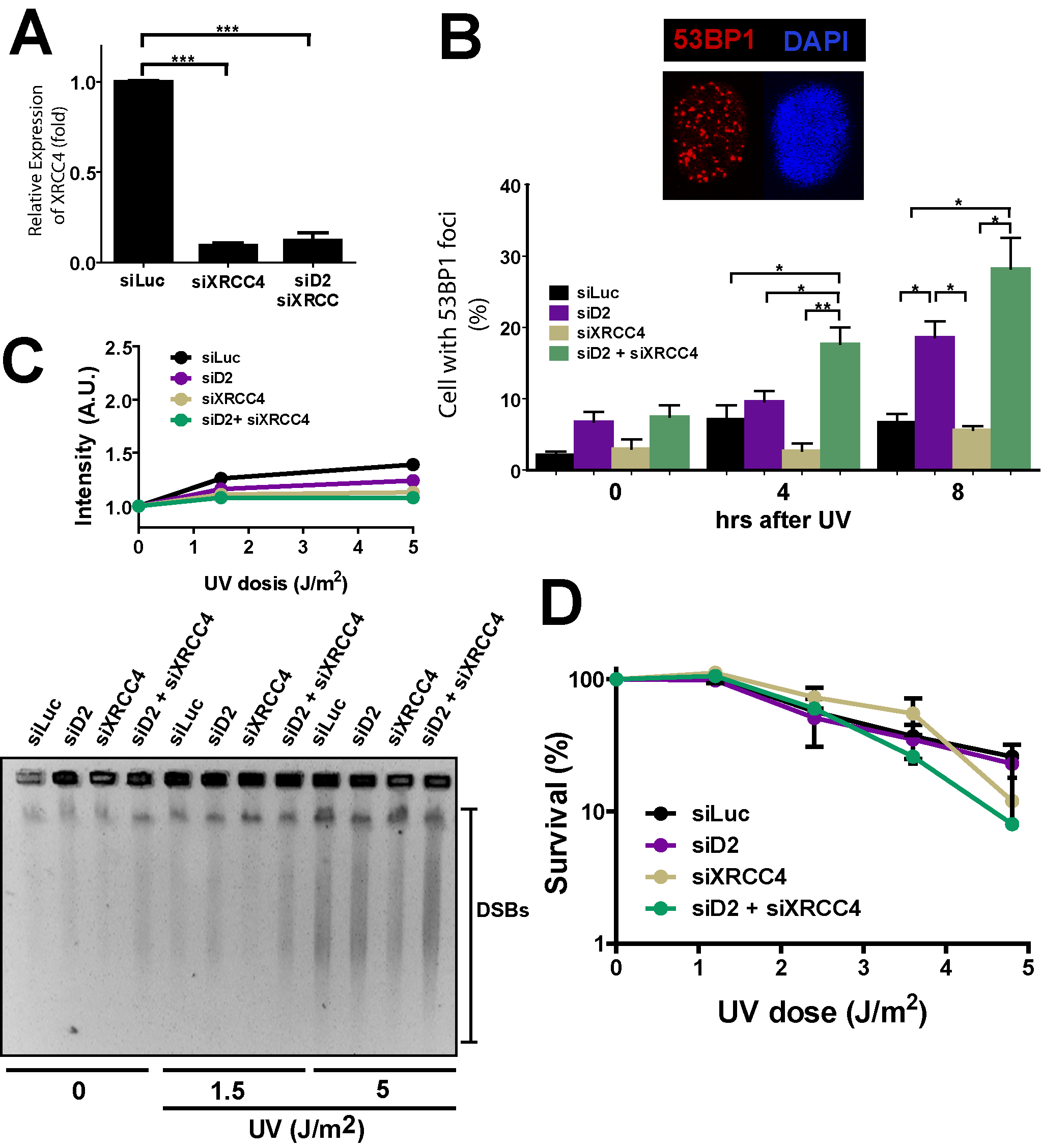

Supplement: S6 Fig — A) Quantitative real-time RT-PCR of XRCC4 was performed in U2OS cells transfected with the indicated siRNAs. Samples were normalized using GAPDH primers. B) 53BP1 focal organization in U2OS cells transfected the indicated siRNAs and UV irradiated with 5 J/m2. Figures are representative of 3 independent experiments. C) Pulse field gel electrophoresis showing the levels of DSB formation after 6 hours of UV irradiation in U2OS transfected with the indicated siRNA. D) Clonogenic assay was evaluated in U2OS transfected with the indicated siRNA. (TIF) [file pgen.1005792.s006.tif]

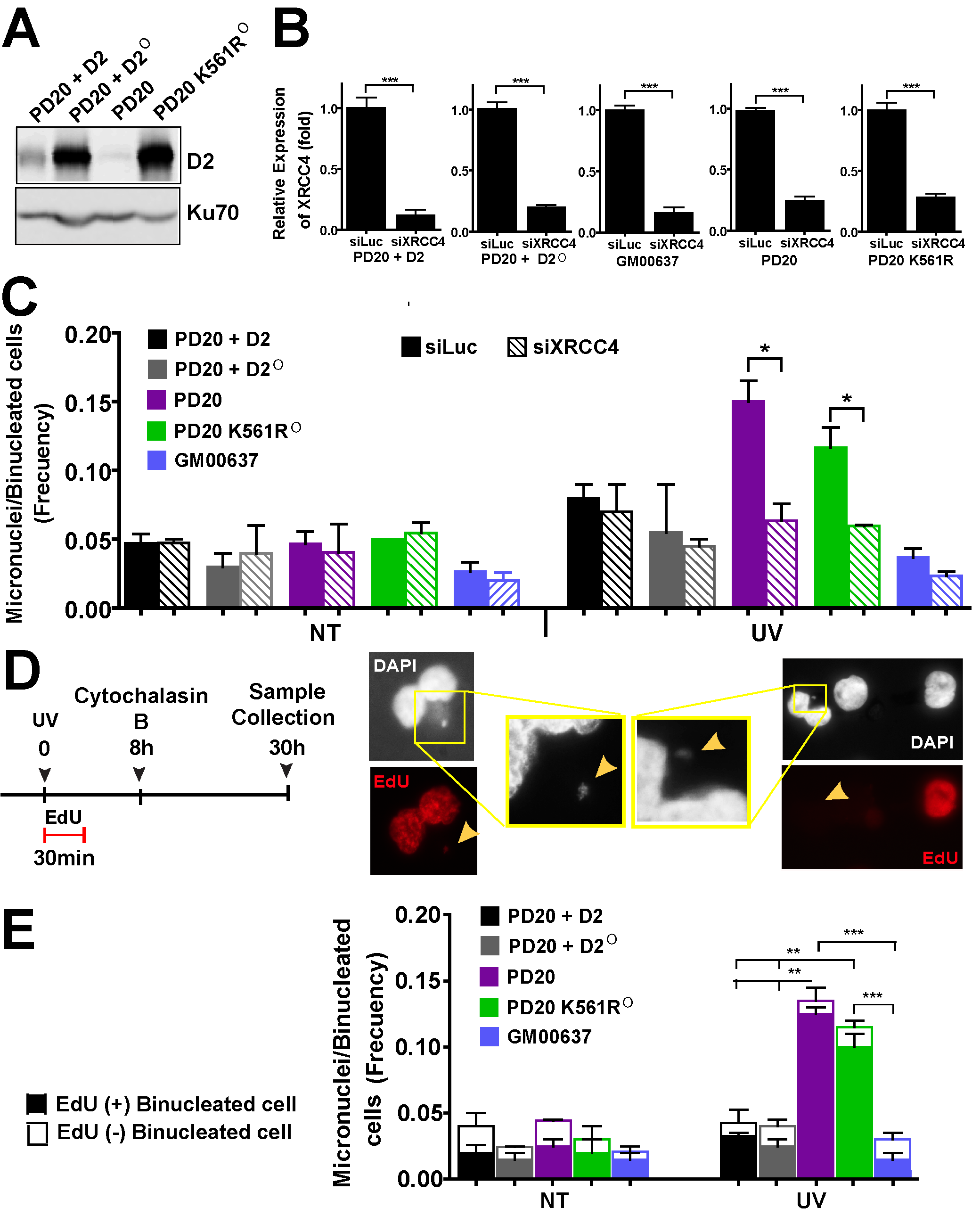

Supplement: S7 Fig — A) W.B. showing FANCD2 levels in PD20 cells and in PD20 cells complemented with FANCD2 (PD20 +D2), overexpressing FANCD2 (PD20+D2O) and the K561R FANCD2 mutant (PD20 +D2 K561RO). B) Quantitative real-time RT-PCR of XRCC4 was performed in PD20 cells lines described in A. Samples were normalized using GAPDH primers. C) MN accumulation in PD20 cells lines transfected with control and XRCC4 siRNA and UV irradiated (5 J/m2). D) Time line depicting the protocol followed to identify binucleated cells which were transiting S phase at the time of UV irradiation. E) Frequency of MN accumulation in Edu (+) and Edu (-) PD20 cells after UV (5 J/m2) irradiation. (TIF) [file pgen.1005792.s007.tif]

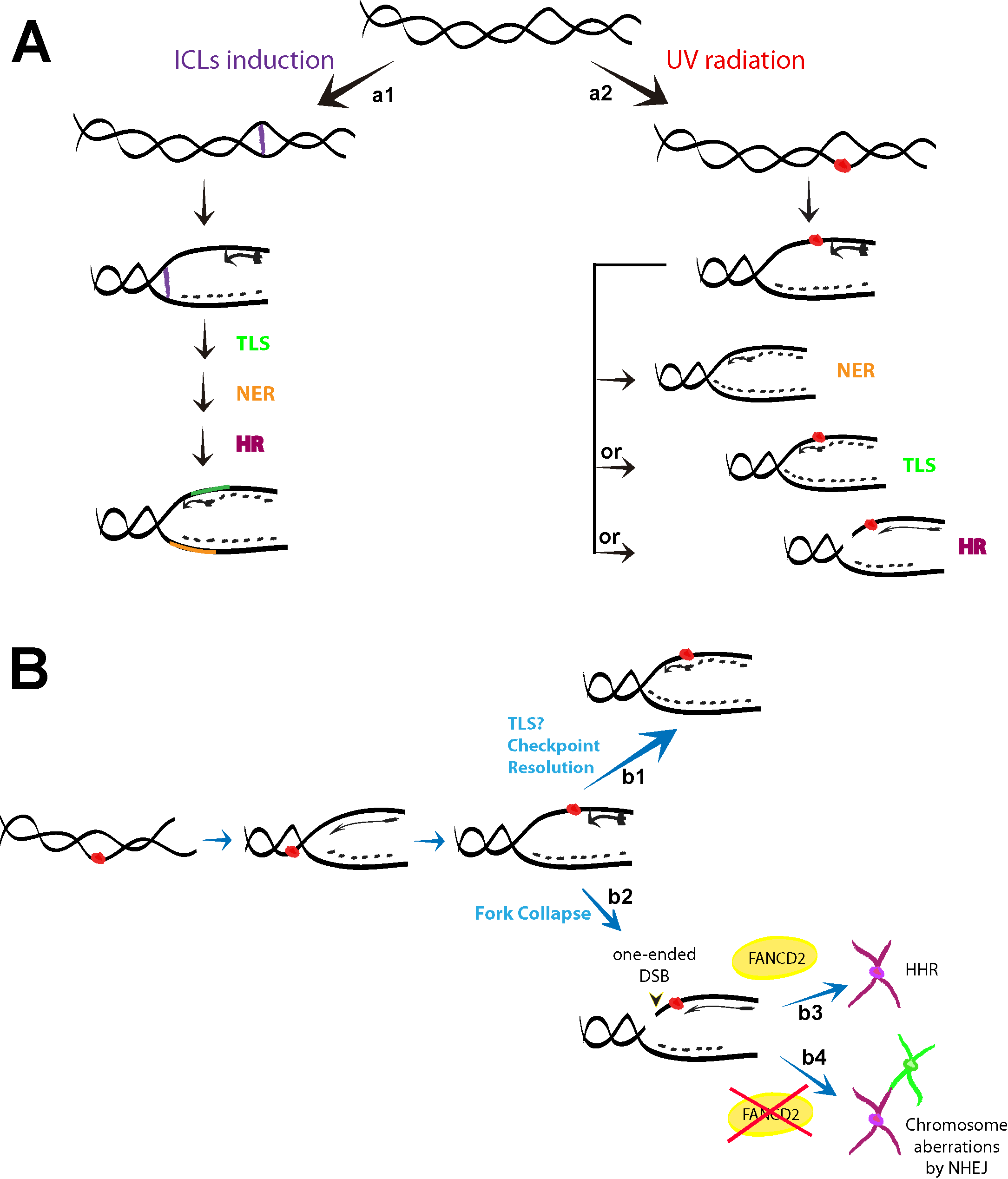

Supplement: S8 Fig — A) The interplay between TLS, NER and HRR at inter- and intra-strand crosslinks. A1) It has been extensively reported in the literature that the FA pathway coordinates the onset of TLS, NER and HRR at ICLs. A2) It has also been reported that TLS, NER and HRR are activated after UV irradiation, albeit in this case, these are independent (not coordinated) events. B) The role of FANCD2 activation after UV irradiation. B1) When UV-triggered DNA lesions are encountered by replication forks TLS aids DNA replication by tolerating the UV-lesion. Checkpoint signals also assists DNA elongation by preventing the collapse of replication forks and promoting the TLS-dependent bypass of DNA lesions [93,97]. B2) However, a fraction of the replication forks that encounter DNA lesions are permanently/irreversibly stalled; such structures may collapse creating one-ended DSBs. B3) FANCD2 (and possibly other components of the canonical FA pathway) promotes HRR at such collapsed forks. B4) The depletion of FANCD2 disfavours the HRR-mediated resolution of replication-associated DSBs after UV irradiation and promotes NHEJ activation, thus jeopardizing the integrity of chromosomes. (TIF) [file pgen.1005792.s008.tif]
