# Supplementary figures and images for: Correction: Chromosomal Integrity after UV Irradiation Requires FANCD2-Mediated Repair of Double Strand Breaks
Source: PLoS Genet. 2023 Dec 20;19(12):e1011094. doi: 10.1371/journal.pgen.1011094 (PMC10732383; doi:10.1371/journal.pgen.1011094)

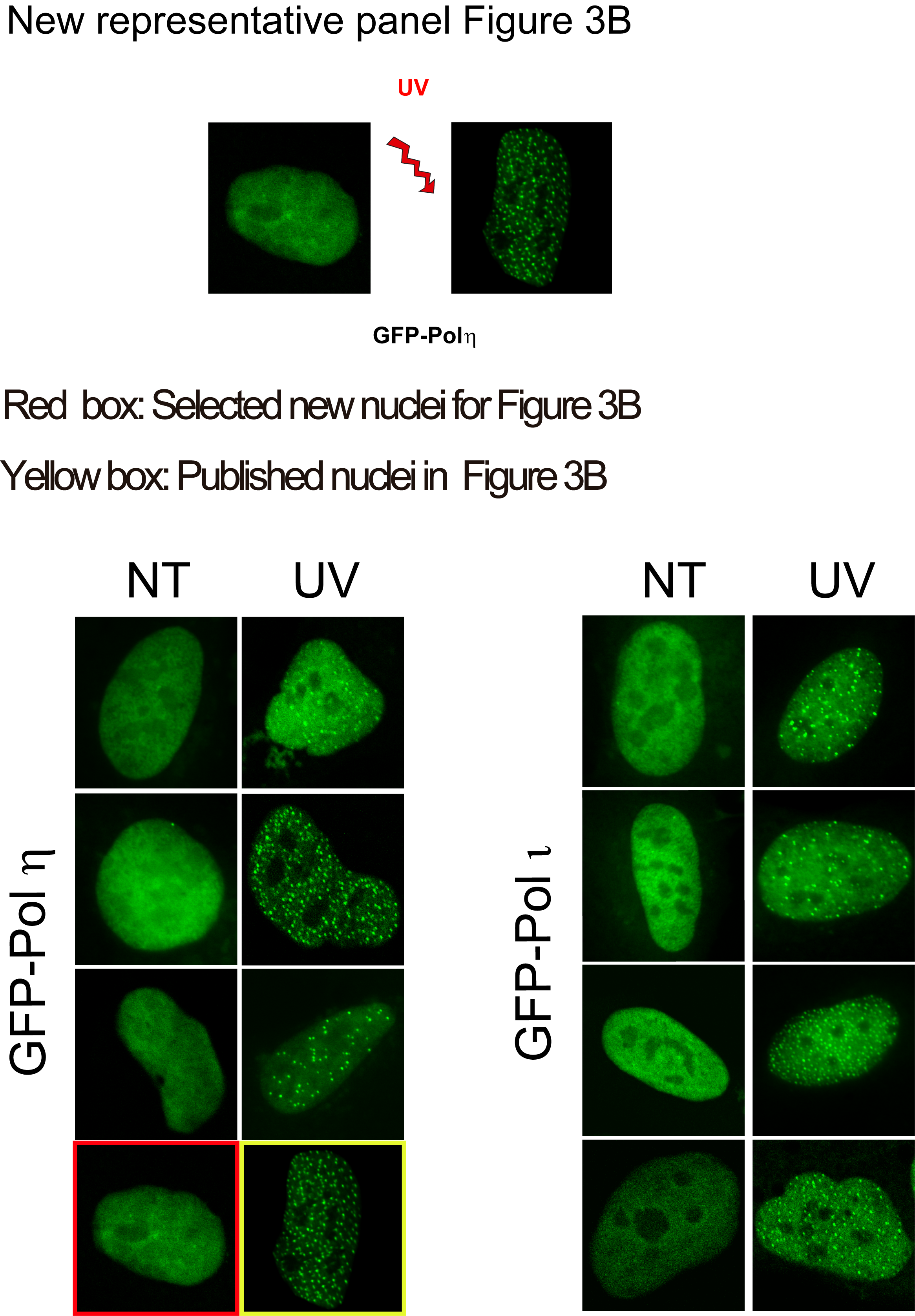

Supplement: S1 File — (TIF) [file pgen.1011094.s001.tif]

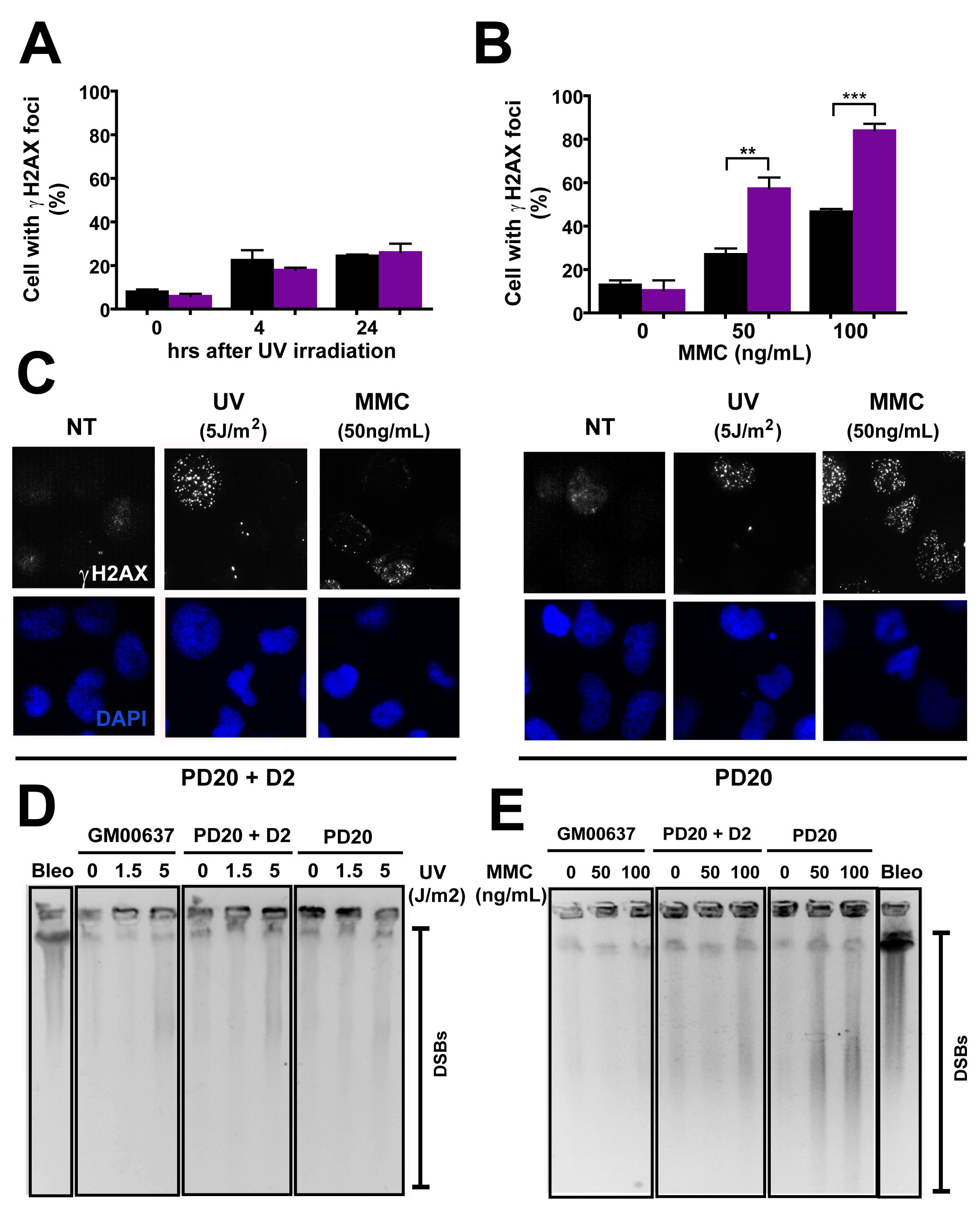

Supplement: S3 File — γH2AX and DAPI panels in PD20 cells without UV irradiation (NT) have been replaced. (TIF) [file pgen.1011094.s003.tif]

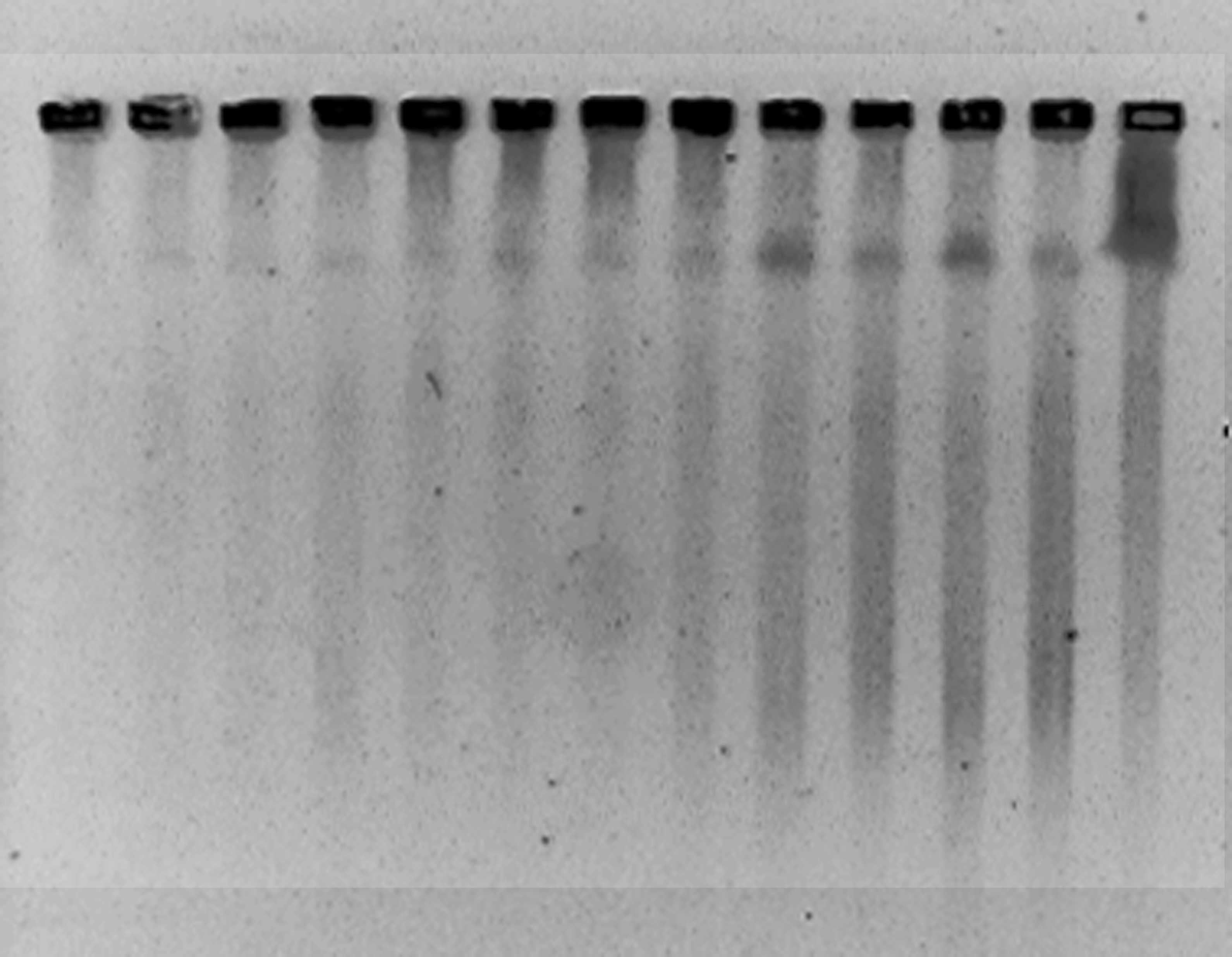

Supplement: S5 File — (A) Annotated original Western blot images, and (B) individual original Western blot images. (ZIP) [file pgen.1011094.s005.zip › S5 File. Original western blot images/S5B. Original western blot images/Tiffs PFGE/Figure 6A.tif]

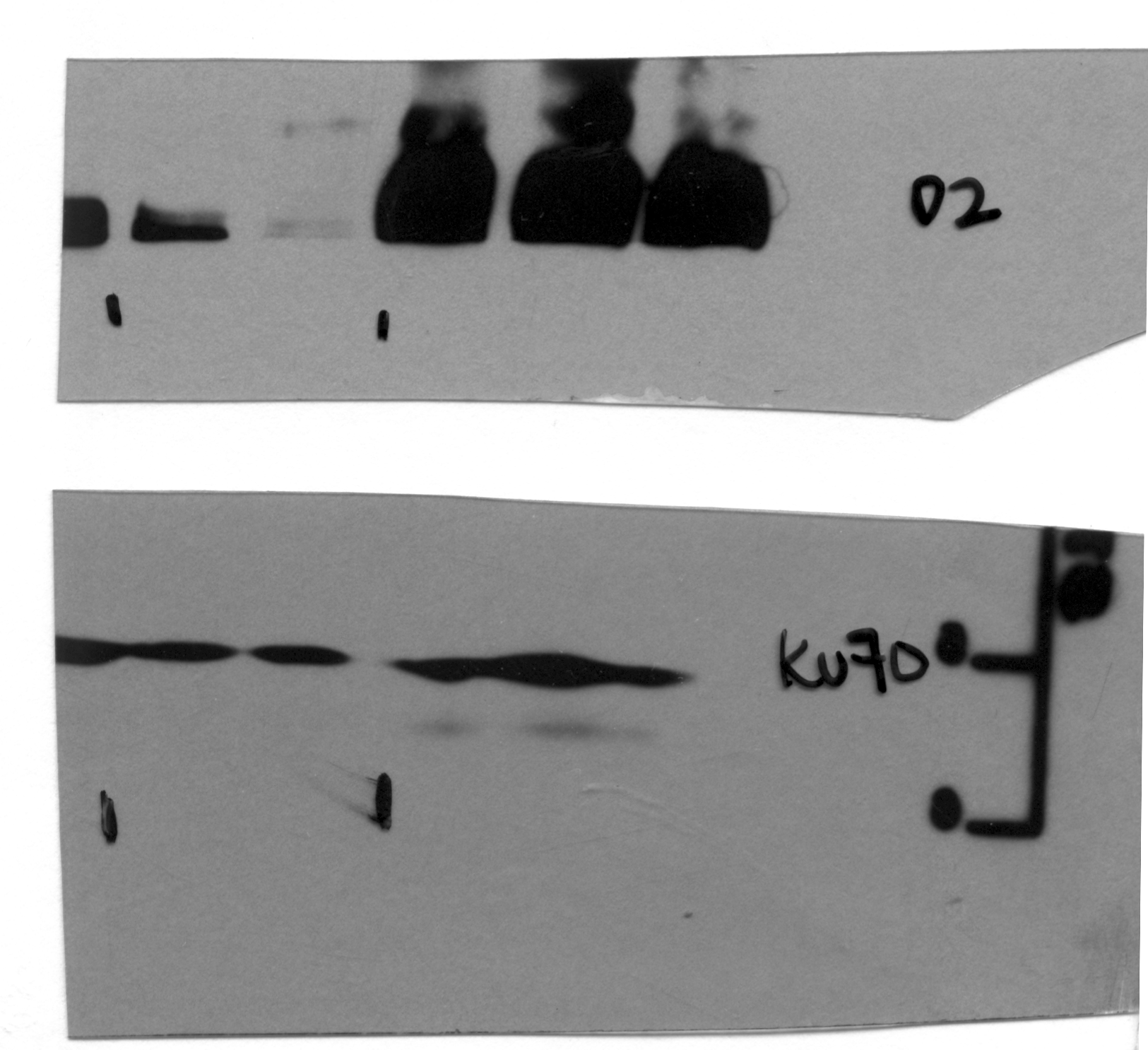

Supplement: S5 File — (A) Annotated original Western blot images, and (B) individual original Western blot images. (ZIP) [file pgen.1011094.s005.zip › S5 File. Original western blot images/S5B. Original western blot images/Tiffs WB/Figure 1A D2 and Ku70 Lower panel.tif]

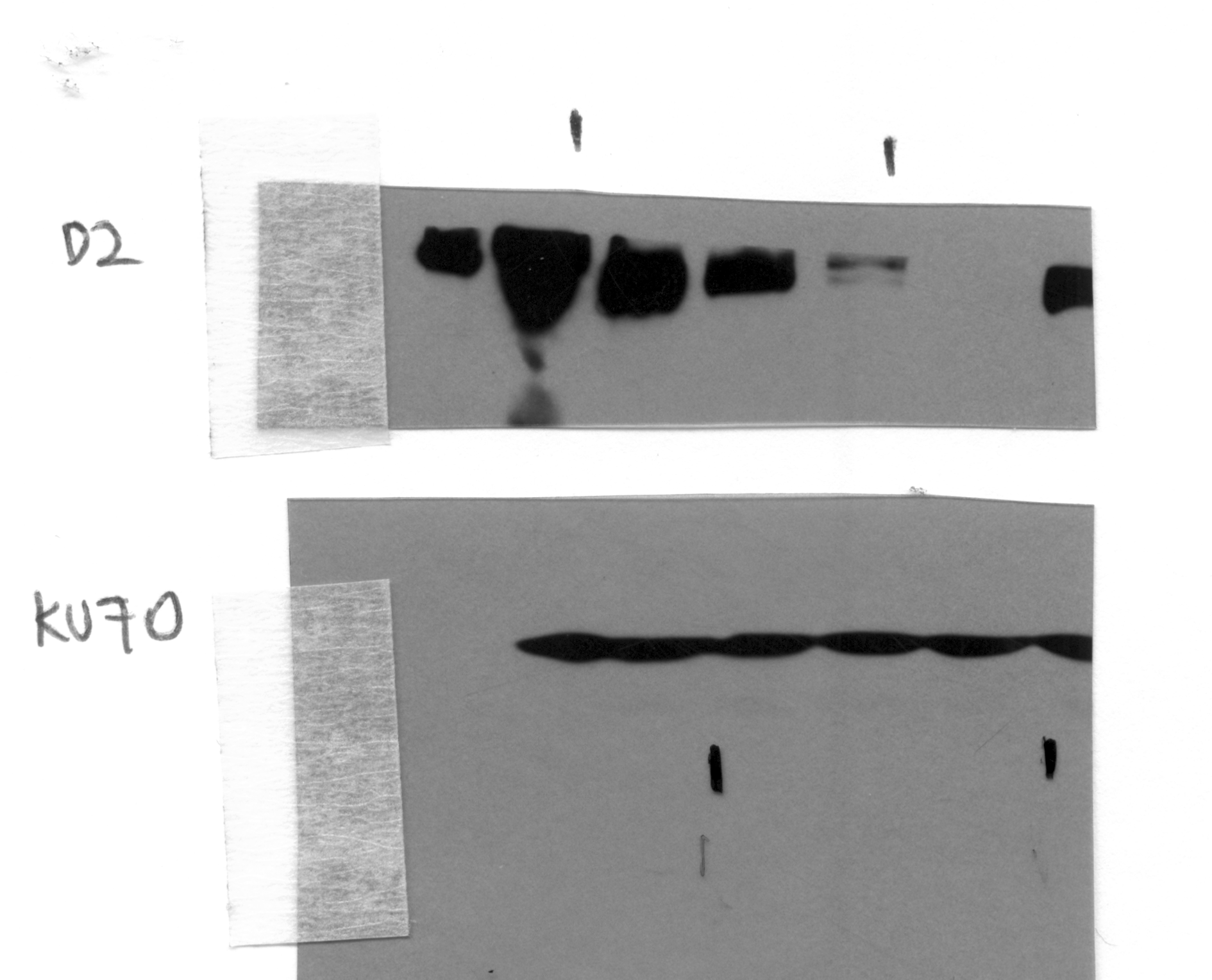

Supplement: S5 File — (A) Annotated original Western blot images, and (B) individual original Western blot images. (ZIP) [file pgen.1011094.s005.zip › S5 File. Original western blot images/S5B. Original western blot images/Tiffs WB/Figure 2C D2 and Ku70.tif]

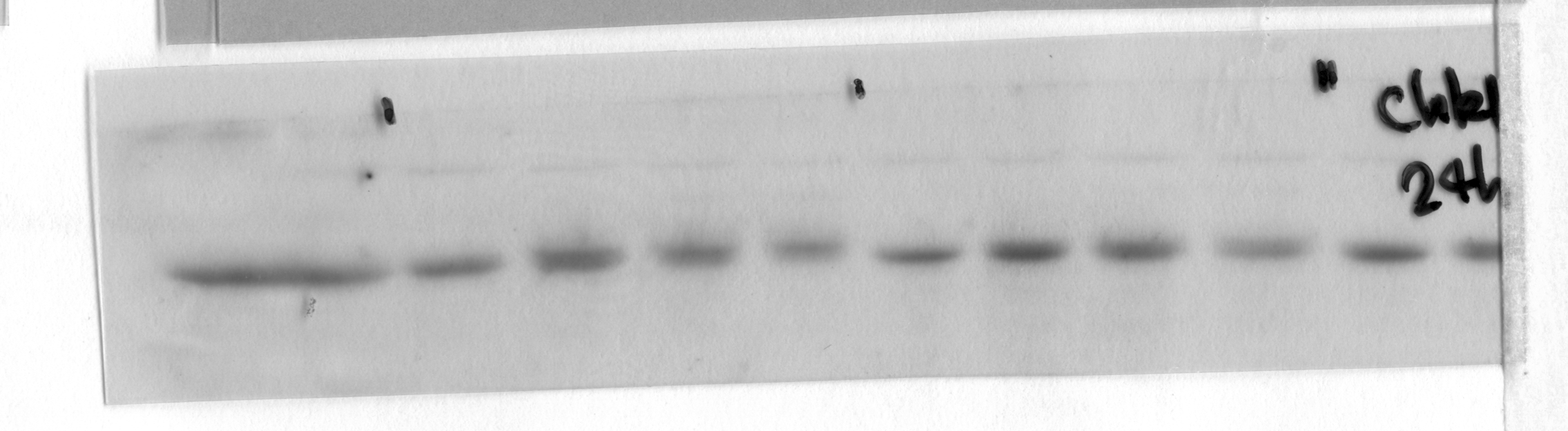

Supplement: S5 File — (A) Annotated original Western blot images, and (B) individual original Western blot images. (ZIP) [file pgen.1011094.s005.zip › S5 File. Original western blot images/S5B. Original western blot images/Tiffs WB/Figure 3C Chk1 24hs.tif]

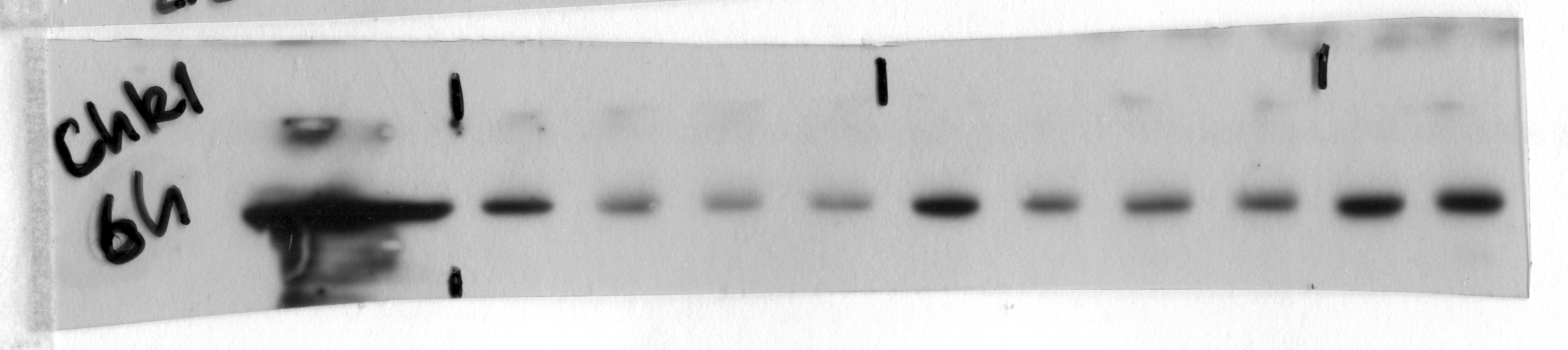

Supplement: S5 File — (A) Annotated original Western blot images, and (B) individual original Western blot images. (ZIP) [file pgen.1011094.s005.zip › S5 File. Original western blot images/S5B. Original western blot images/Tiffs WB/Figure 3C Chk1 6hs.tif]

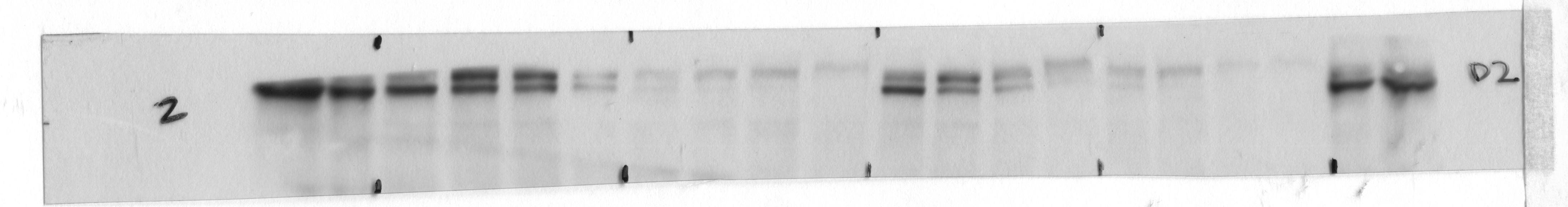

Supplement: S5 File — (A) Annotated original Western blot images, and (B) individual original Western blot images. (ZIP) [file pgen.1011094.s005.zip › S5 File. Original western blot images/S5B. Original western blot images/Tiffs WB/Figure 3C D2 24hs.tif]

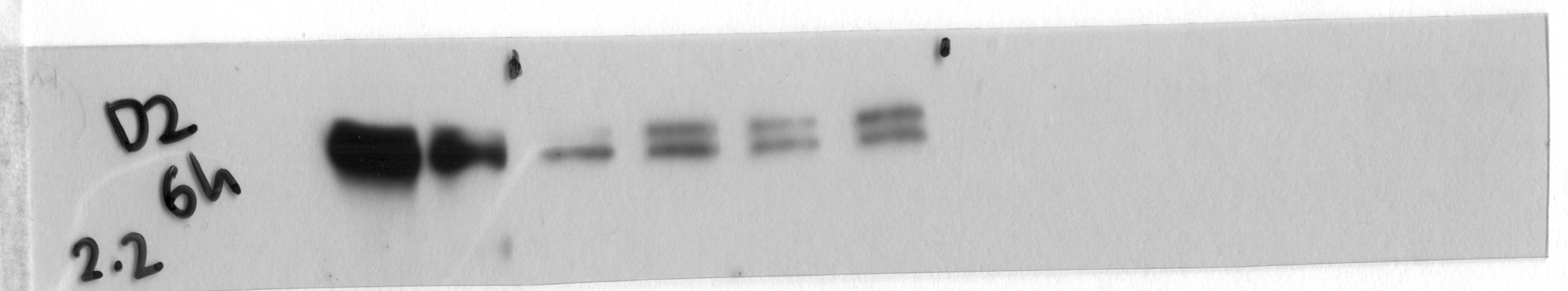

Supplement: S5 File — (A) Annotated original Western blot images, and (B) individual original Western blot images. (ZIP) [file pgen.1011094.s005.zip › S5 File. Original western blot images/S5B. Original western blot images/Tiffs WB/Figure 3C D2 6hs.tif]

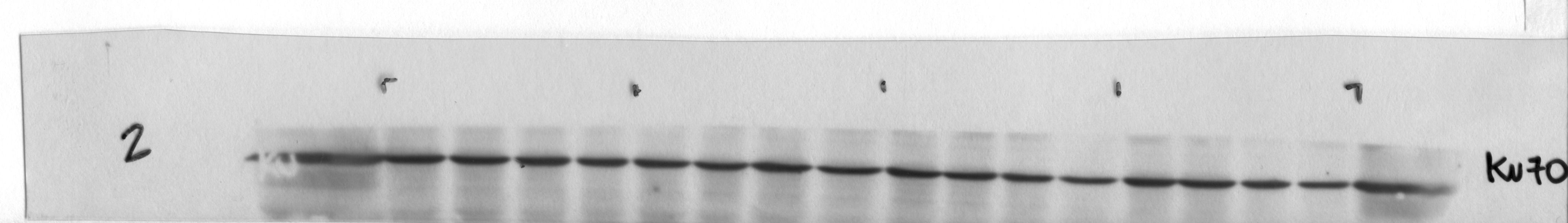

Supplement: S5 File — (A) Annotated original Western blot images, and (B) individual original Western blot images. (ZIP) [file pgen.1011094.s005.zip › S5 File. Original western blot images/S5B. Original western blot images/Tiffs WB/Figure 3C Ku70 6hs and 24hs.tif]

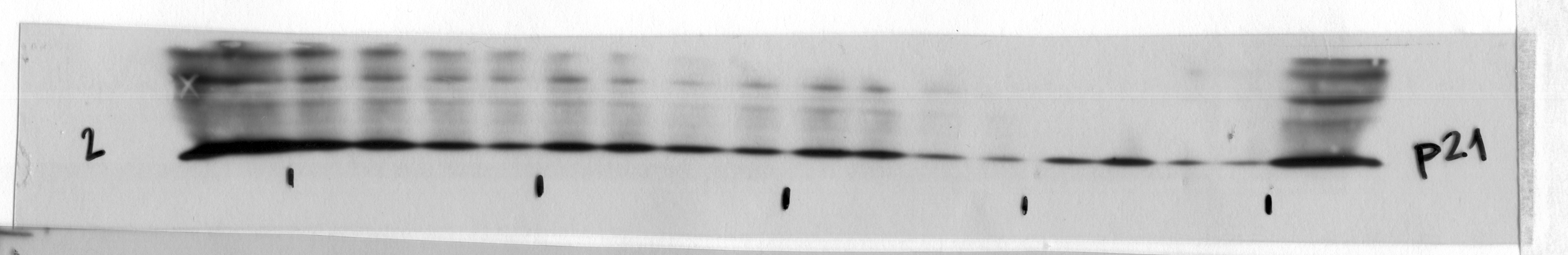

Supplement: S5 File — (A) Annotated original Western blot images, and (B) individual original Western blot images. (ZIP) [file pgen.1011094.s005.zip › S5 File. Original western blot images/S5B. Original western blot images/Tiffs WB/Figure 3C p21 6hs and 24hs.tif]

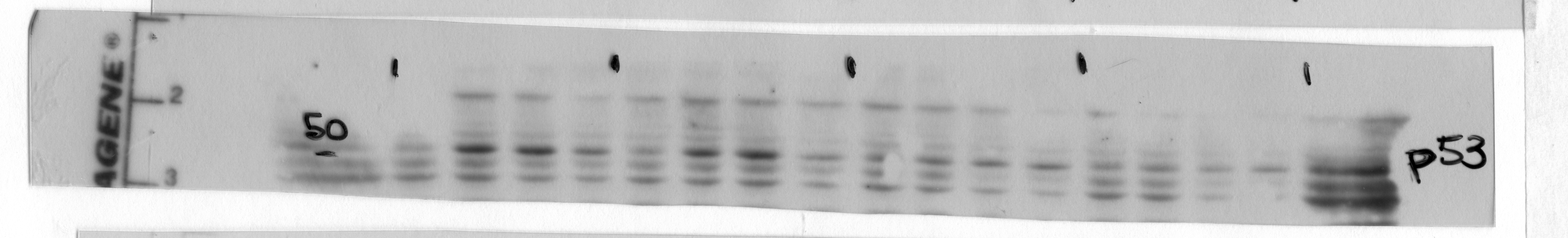

Supplement: S5 File — (A) Annotated original Western blot images, and (B) individual original Western blot images. (ZIP) [file pgen.1011094.s005.zip › S5 File. Original western blot images/S5B. Original western blot images/Tiffs WB/Figure 3C p53 6hs and 24hs.tif]

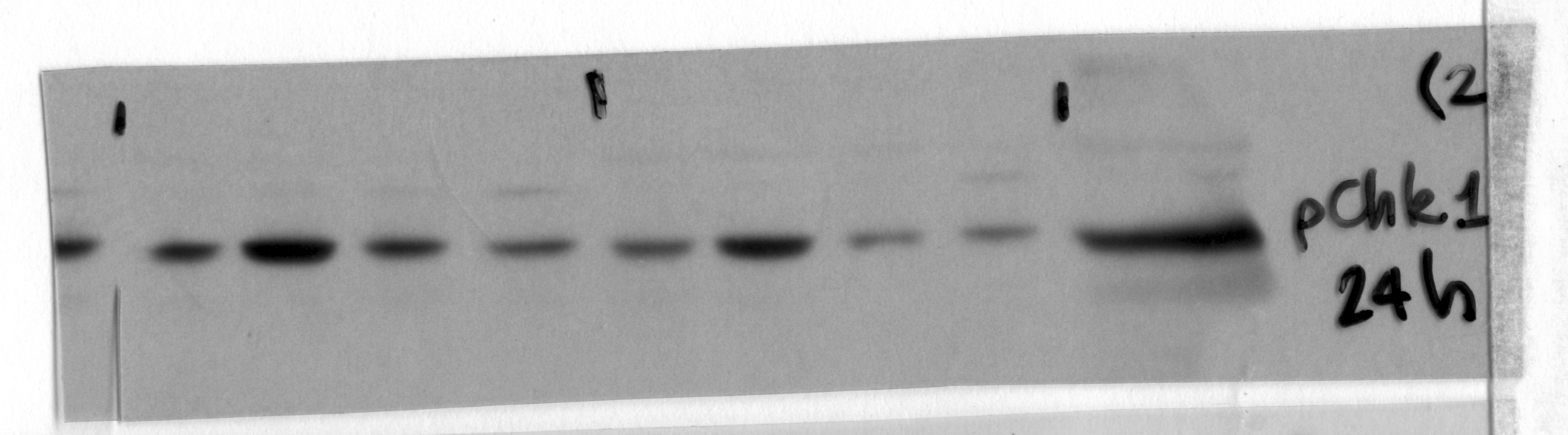

Supplement: S5 File — (A) Annotated original Western blot images, and (B) individual original Western blot images. (ZIP) [file pgen.1011094.s005.zip › S5 File. Original western blot images/S5B. Original western blot images/Tiffs WB/Figure 3C pChk1 24hs.tif]

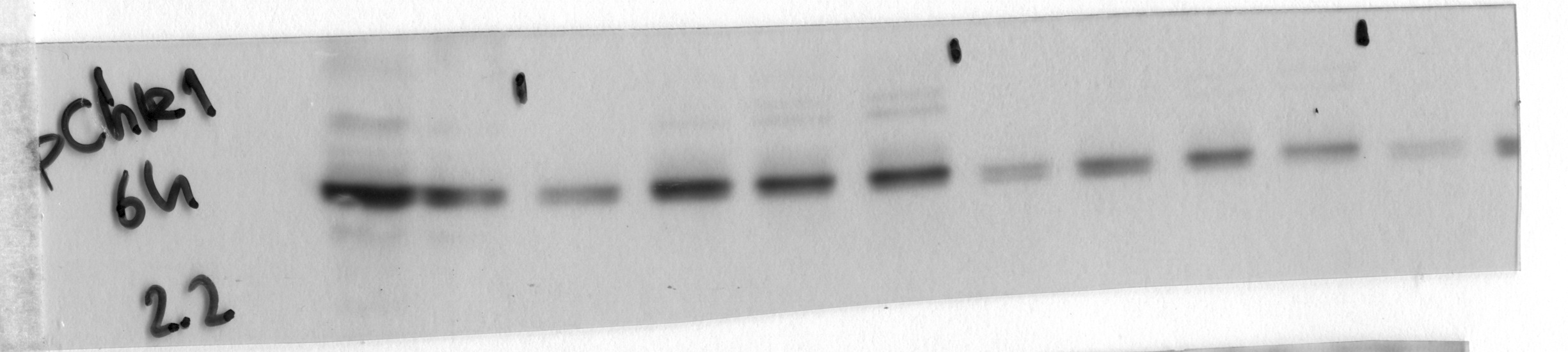

Supplement: S5 File — (A) Annotated original Western blot images, and (B) individual original Western blot images. (ZIP) [file pgen.1011094.s005.zip › S5 File. Original western blot images/S5B. Original western blot images/Tiffs WB/Figure 3C pChk1 6hs.tif]

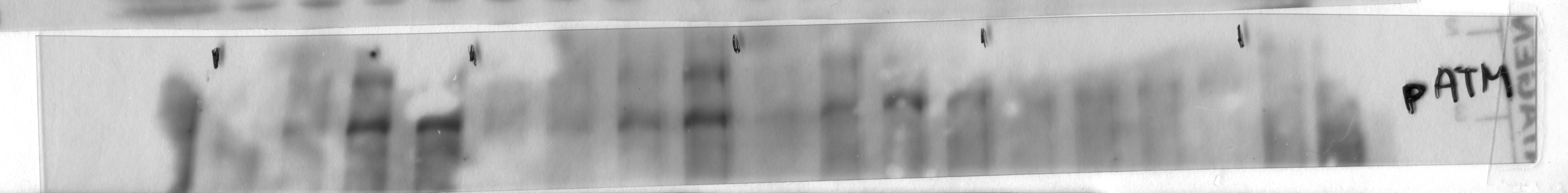

Supplement: S5 File — (A) Annotated original Western blot images, and (B) individual original Western blot images. (ZIP) [file pgen.1011094.s005.zip › S5 File. Original western blot images/S5B. Original western blot images/Tiffs WB/Figure 4D pATM.tif]

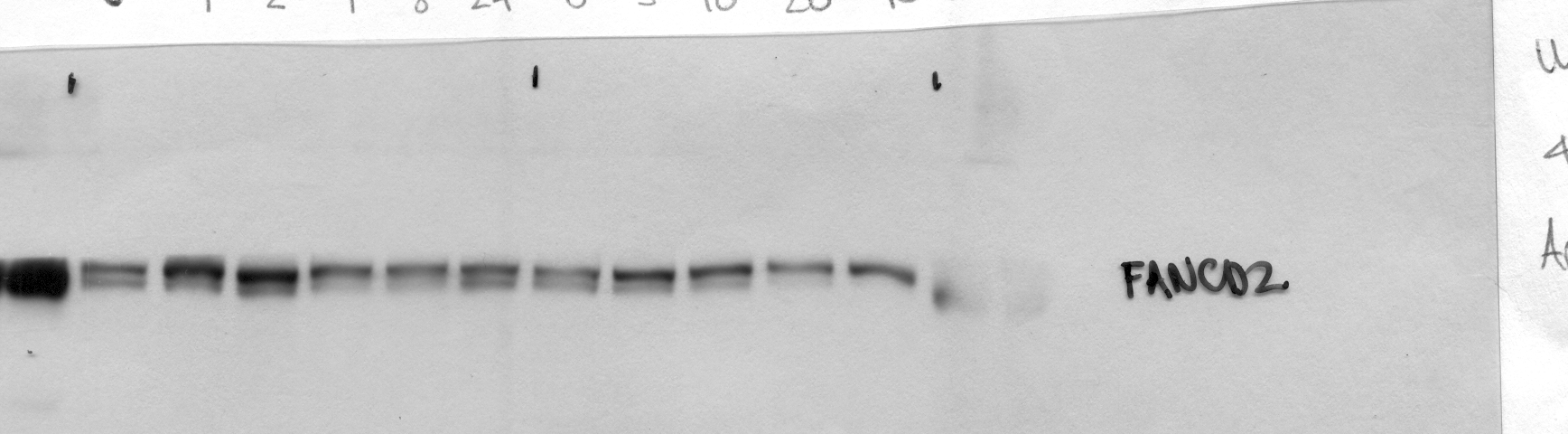

Supplement: S5 File — (A) Annotated original Western blot images, and (B) individual original Western blot images. (ZIP) [file pgen.1011094.s005.zip › S5 File. Original western blot images/S5B. Original western blot images/Tiffs WB/Suplementary Figure 1C and D D2 (PD20+D2).tif]

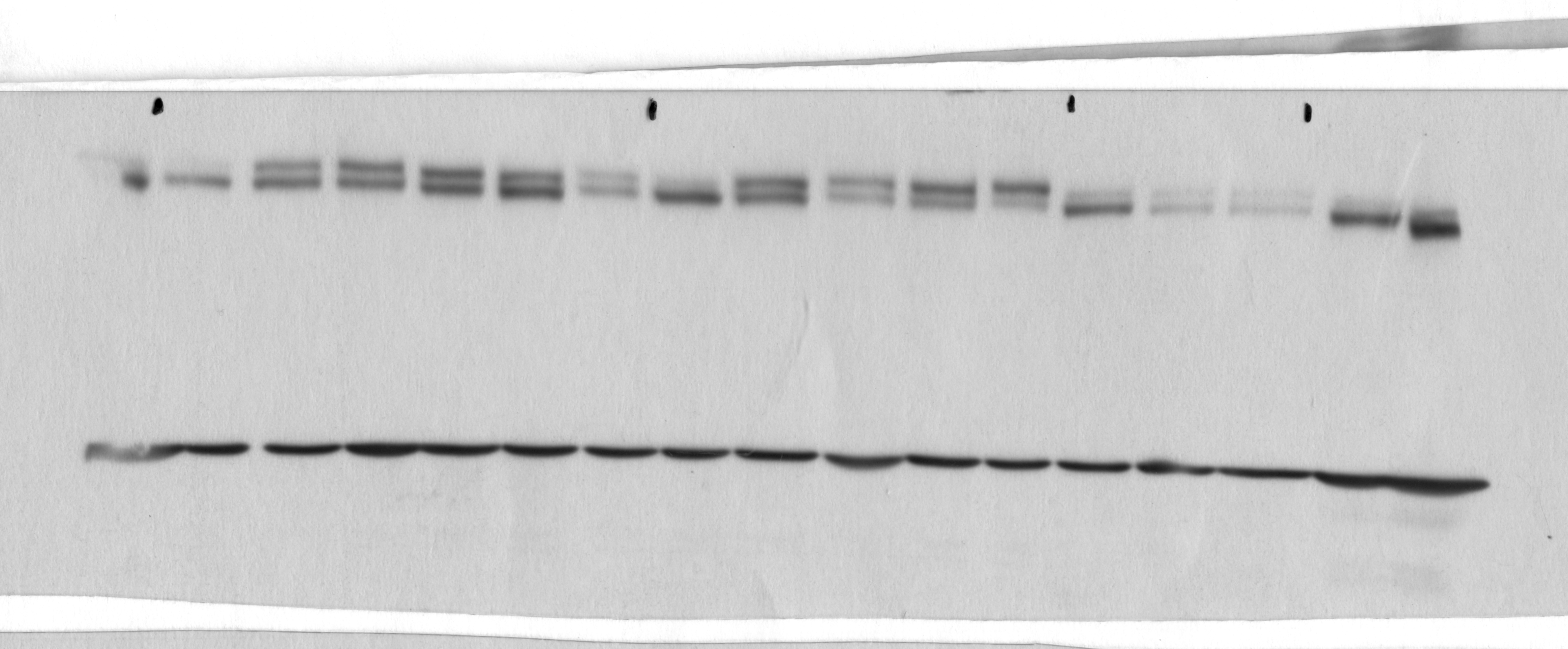

Supplement: S5 File — (A) Annotated original Western blot images, and (B) individual original Western blot images. (ZIP) [file pgen.1011094.s005.zip › S5 File. Original western blot images/S5B. Original western blot images/Tiffs WB/Suplementary Figure 1C and D D2 and Ku70 (U2OS).tif]

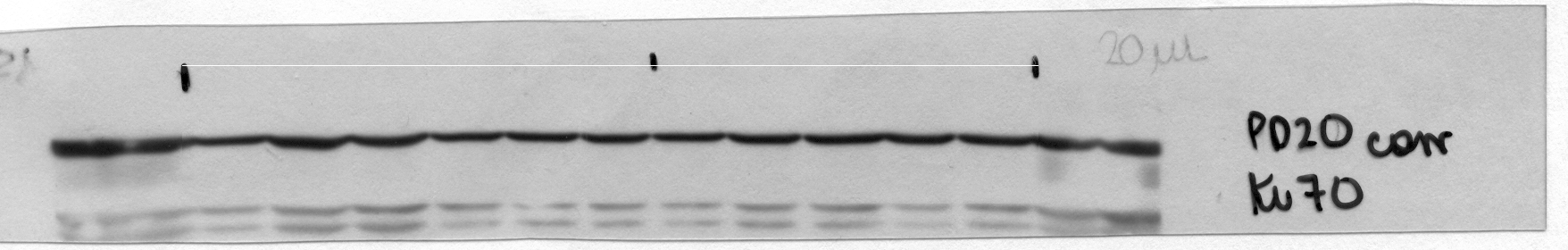

Supplement: S5 File — (A) Annotated original Western blot images, and (B) individual original Western blot images. (ZIP) [file pgen.1011094.s005.zip › S5 File. Original western blot images/S5B. Original western blot images/Tiffs WB/Suplementary Figure 1C and D Ku70 (PD20+D2).tif]

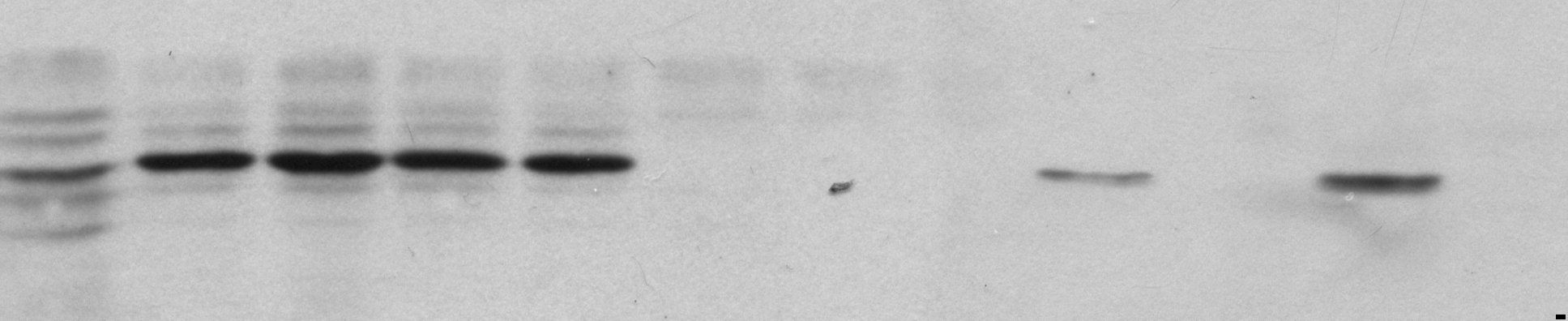

Supplement: S5 File — (A) Annotated original Western blot images, and (B) individual original Western blot images. (ZIP) [file pgen.1011094.s005.zip › S5 File. Original western blot images/S5B. Original western blot images/Tiffs WB/Suplementary Figure 1E Pol eta.tif]

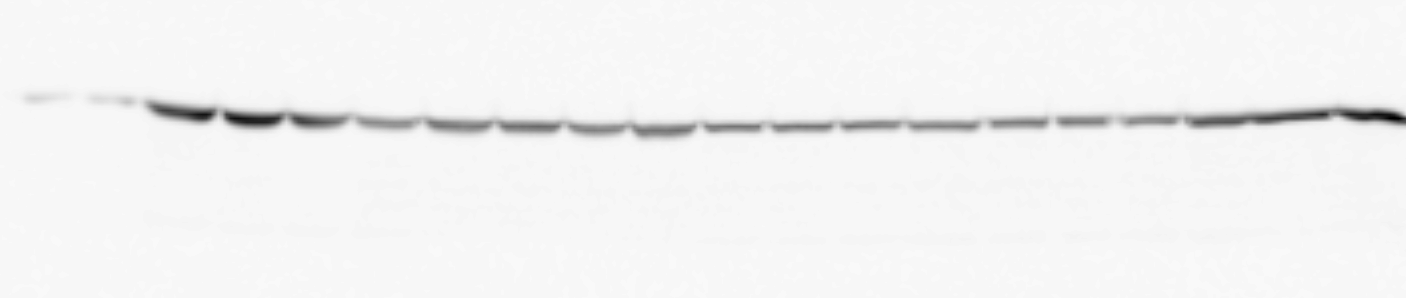

Supplement: S5 File — (A) Annotated original Western blot images, and (B) individual original Western blot images. (ZIP) [file pgen.1011094.s005.zip › S5 File. Original western blot images/S5B. Original western blot images/Tiffs WB/Suplementary Figure 3A Ku70 6 and 24hs.tif]
