# Supplementary material for: Correction: Chromosomal Integrity after UV Irradiation Requires FANCD2-Mediated Repair of Double Strand Breaks
Source: PLoS Genet. 2023 Dec 20;19(12):e1011094. doi: 10.1371/journal.pgen.1011094 (PMC10732383; doi:10.1371/journal.pgen.1011094)

Figure 1A

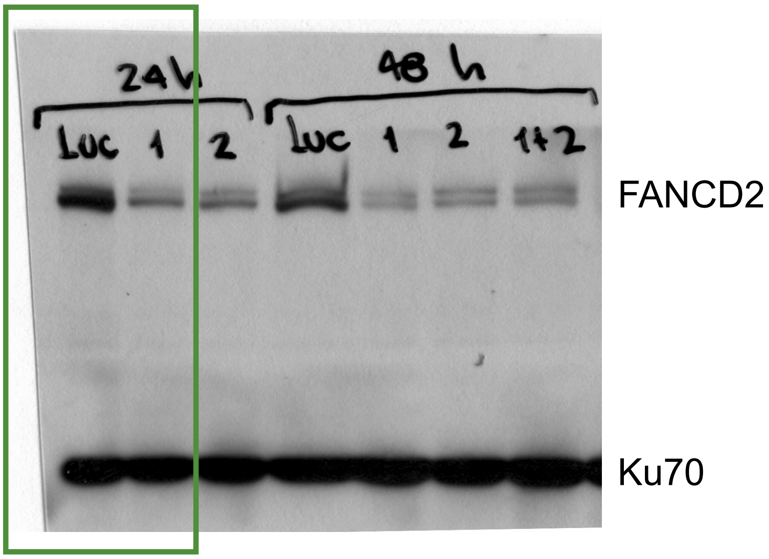

Figure 1A

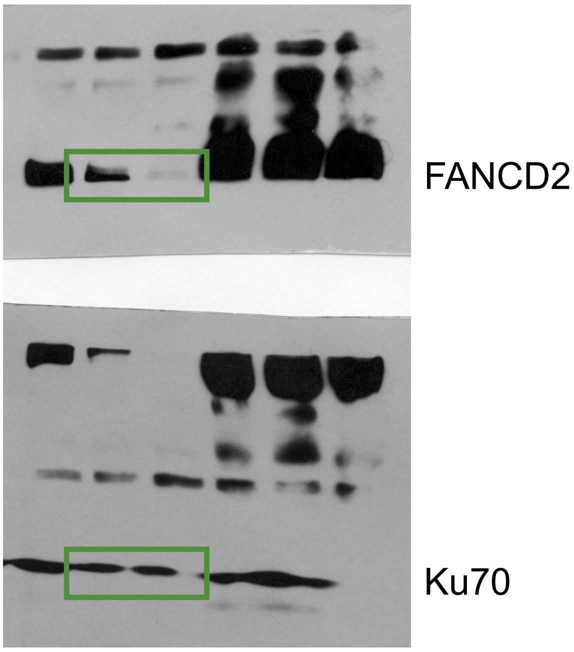

Figure 2C

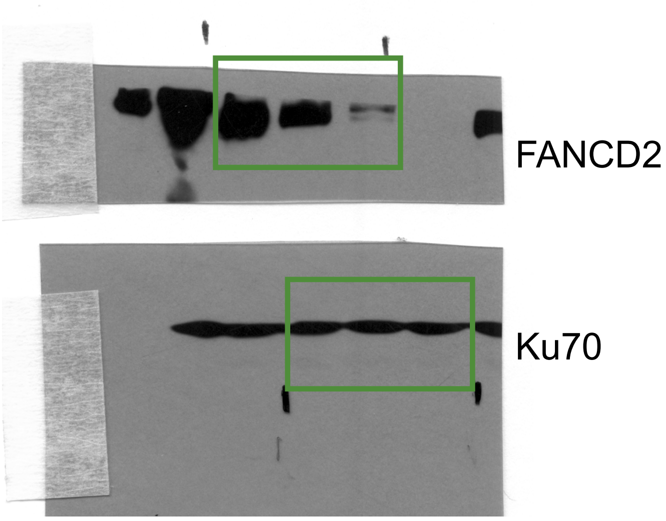

Figure 3A

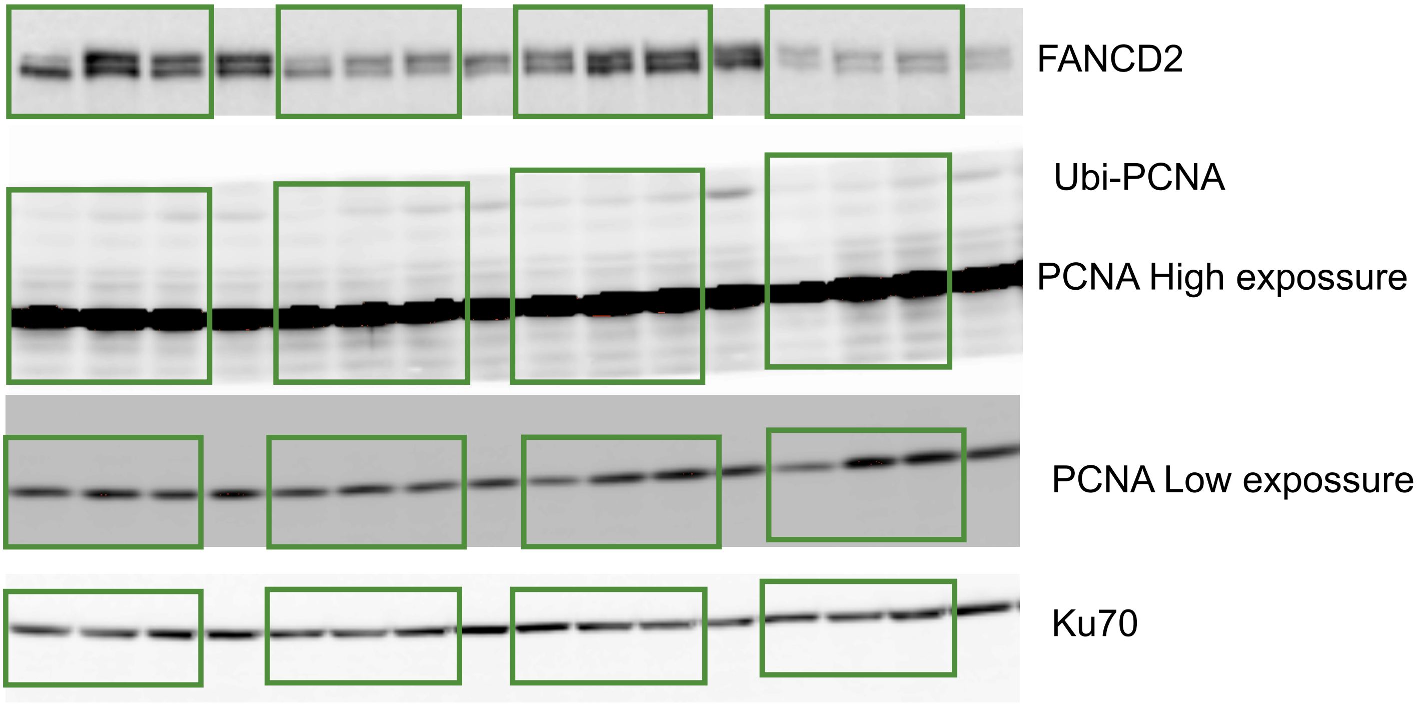

Figure 3C

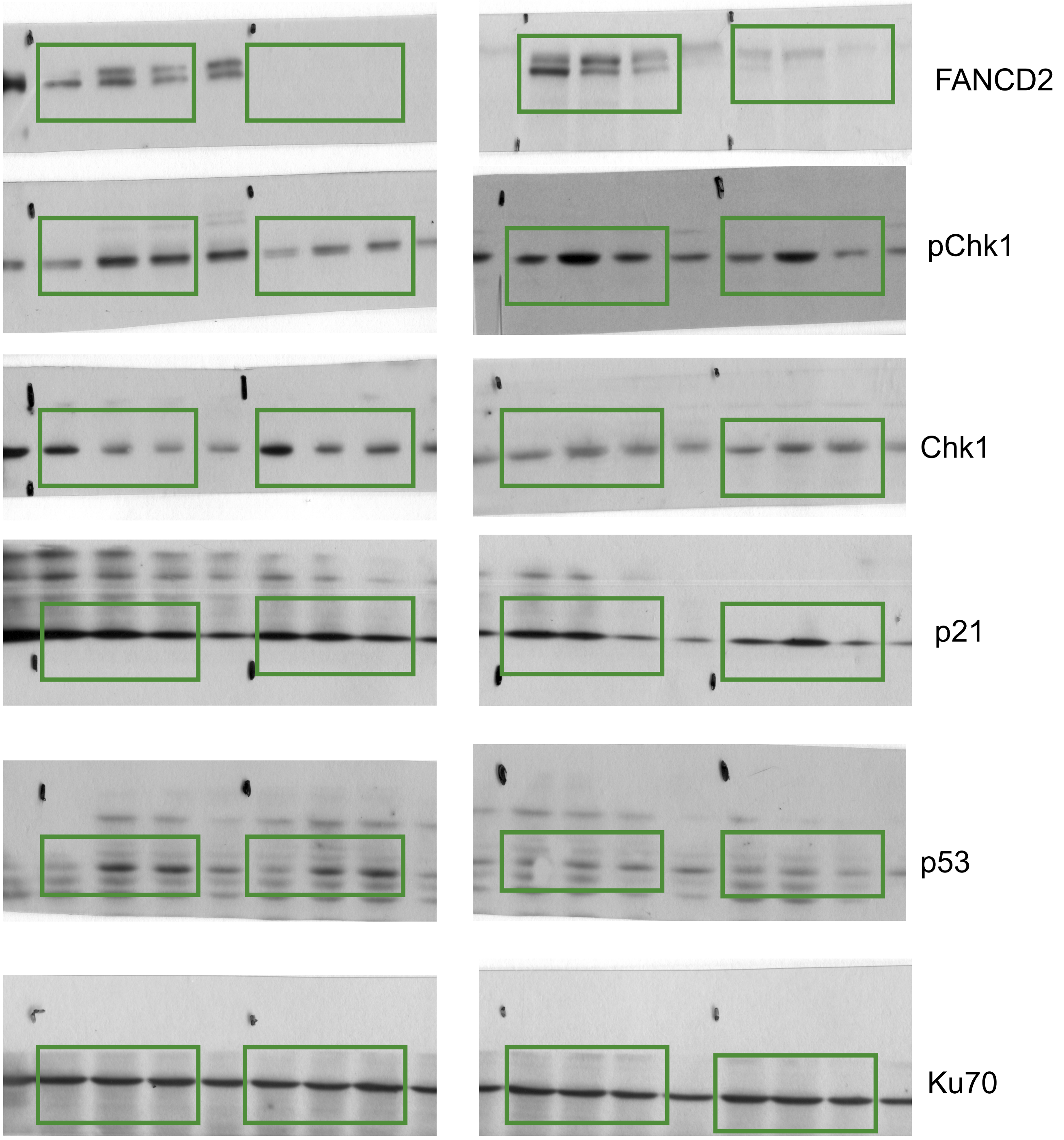

Figure 4D

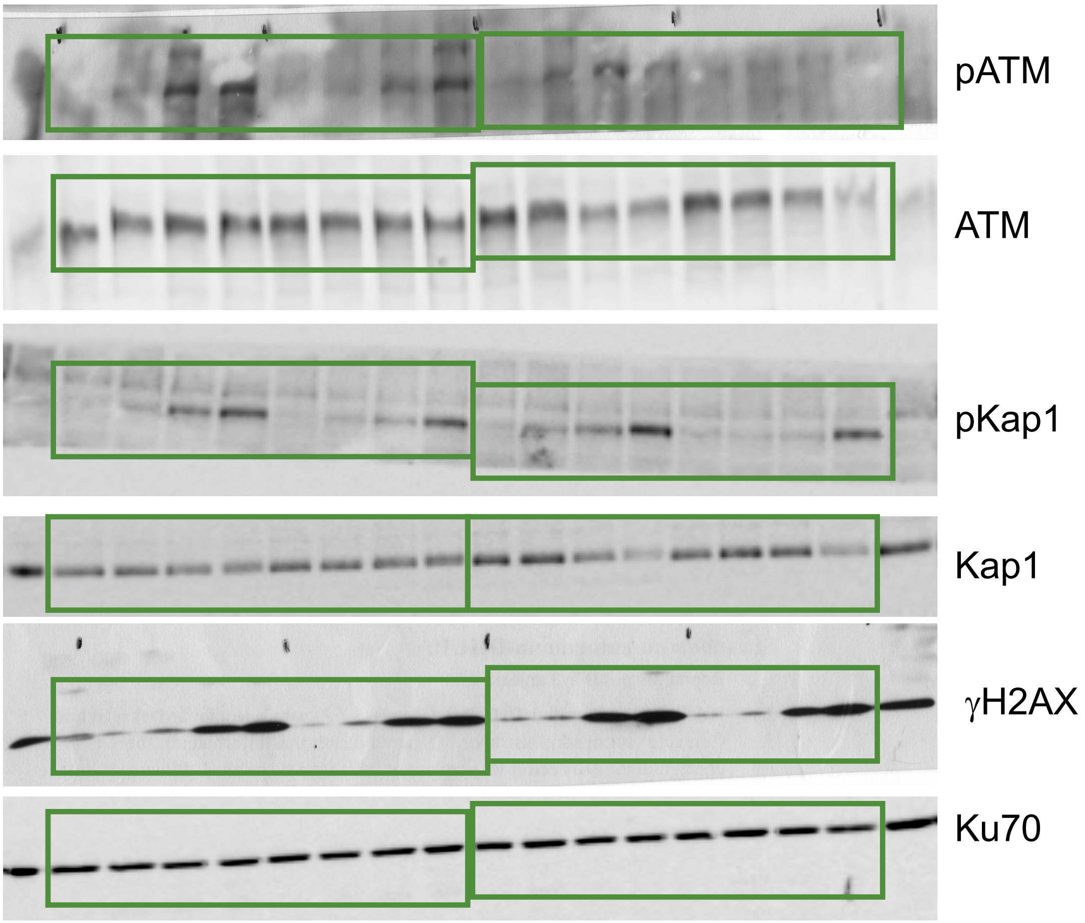

Supplementary Figure 1C

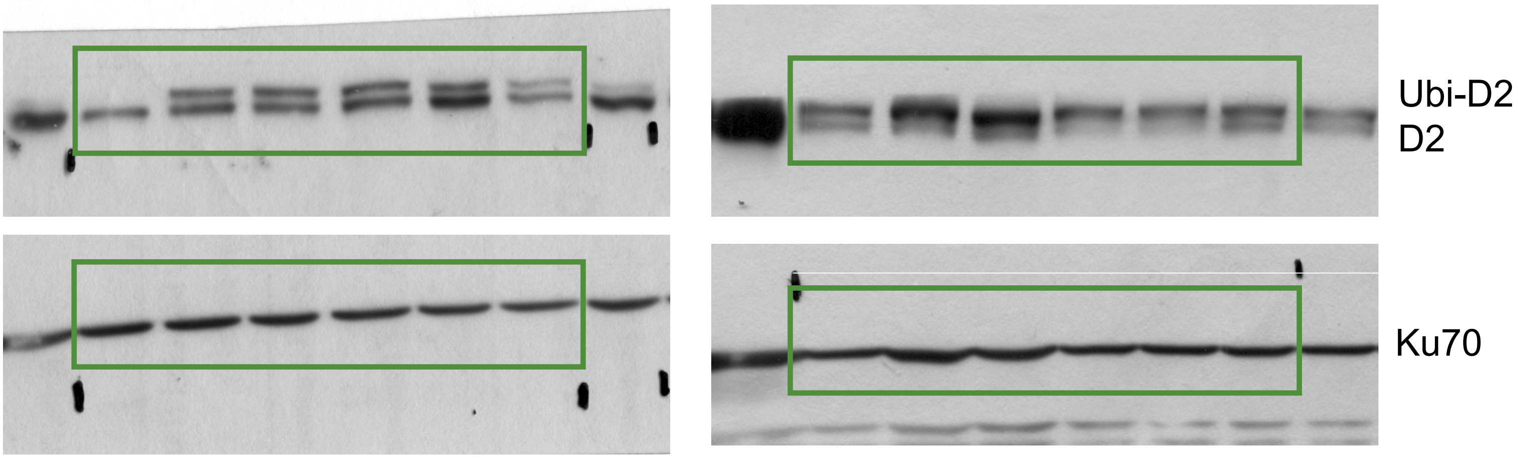

Supplementary Figure 1D

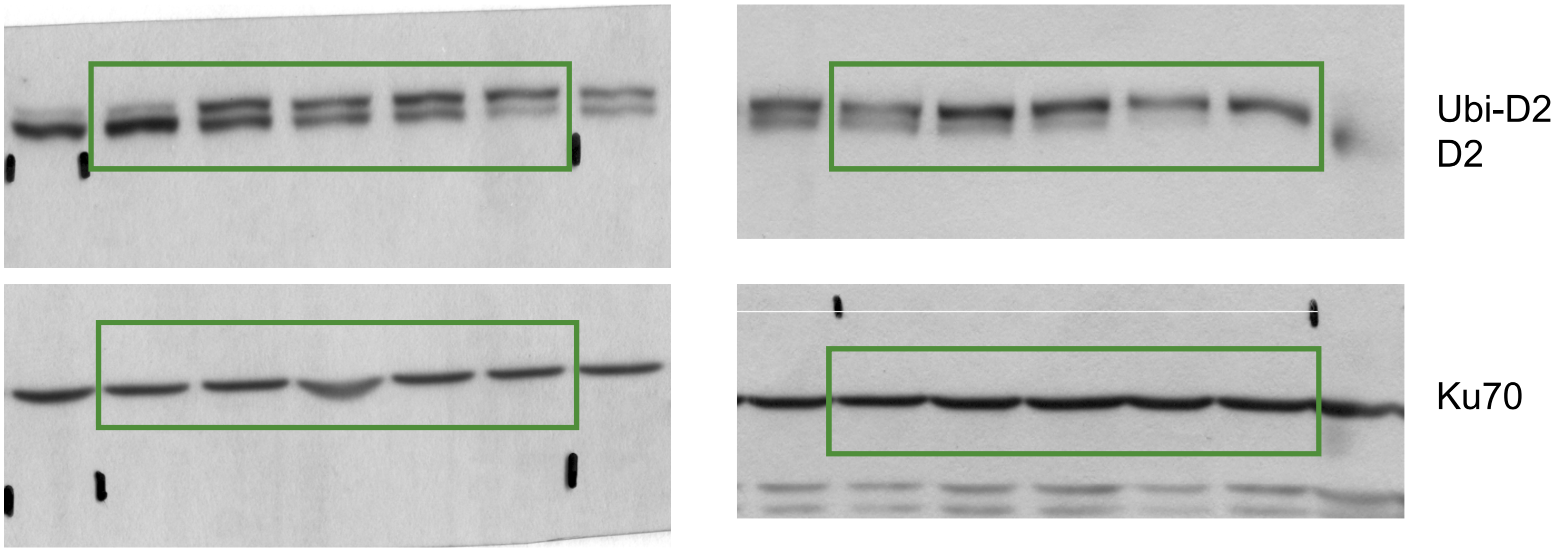

Supplementary Figure 1E

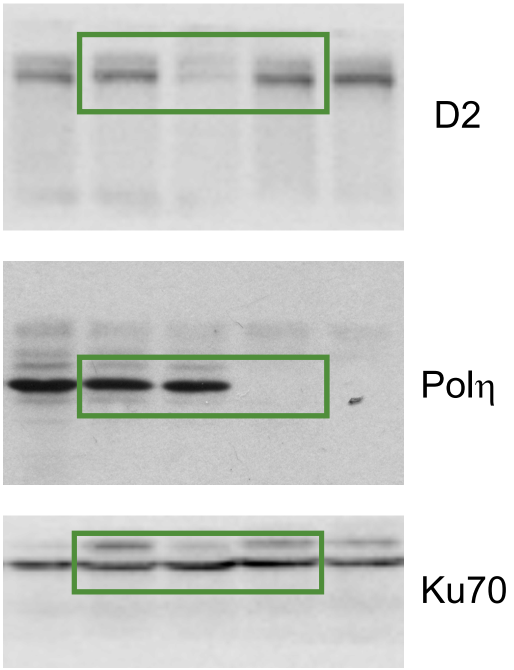

Supplementary Figure 1F

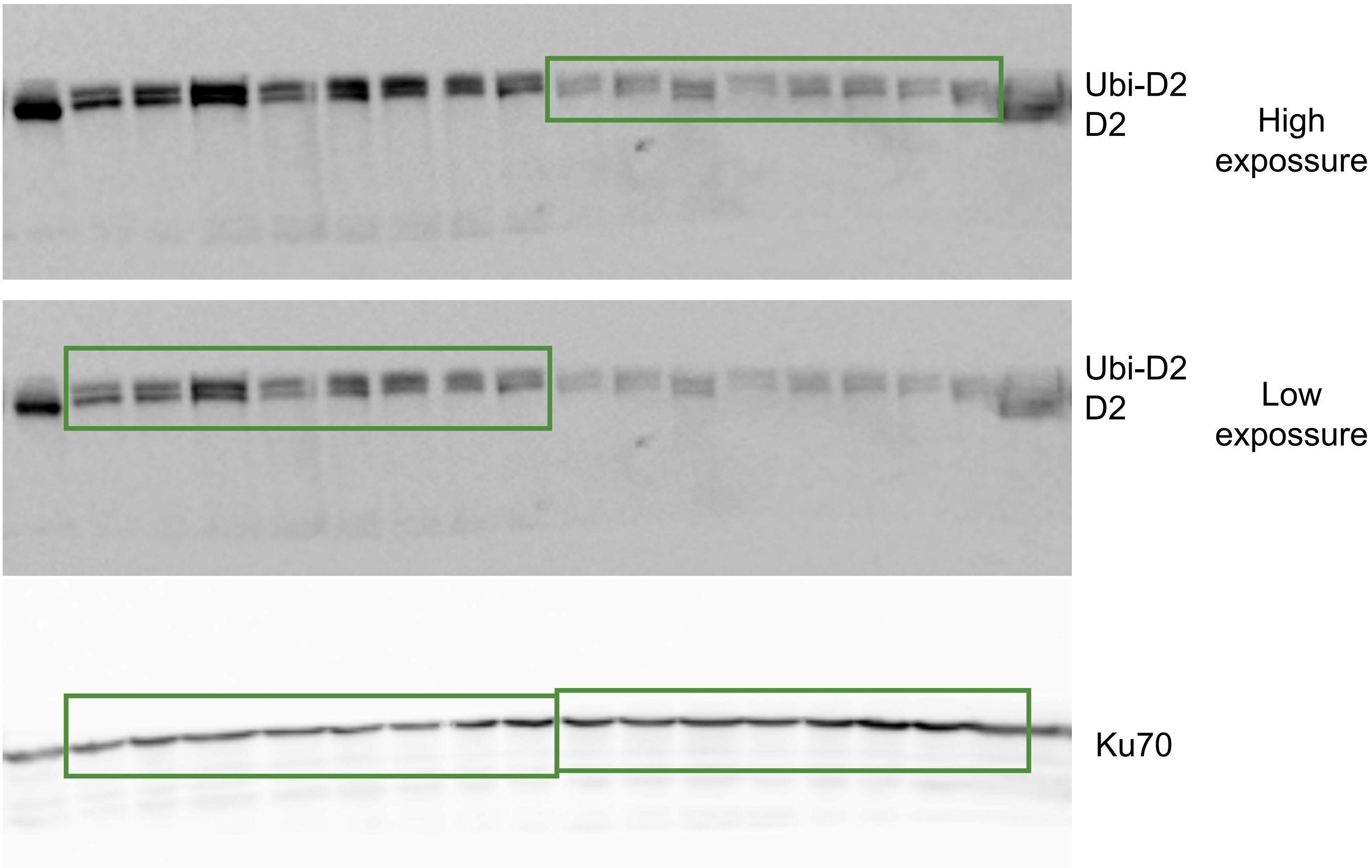

Supplementary Figure 3A

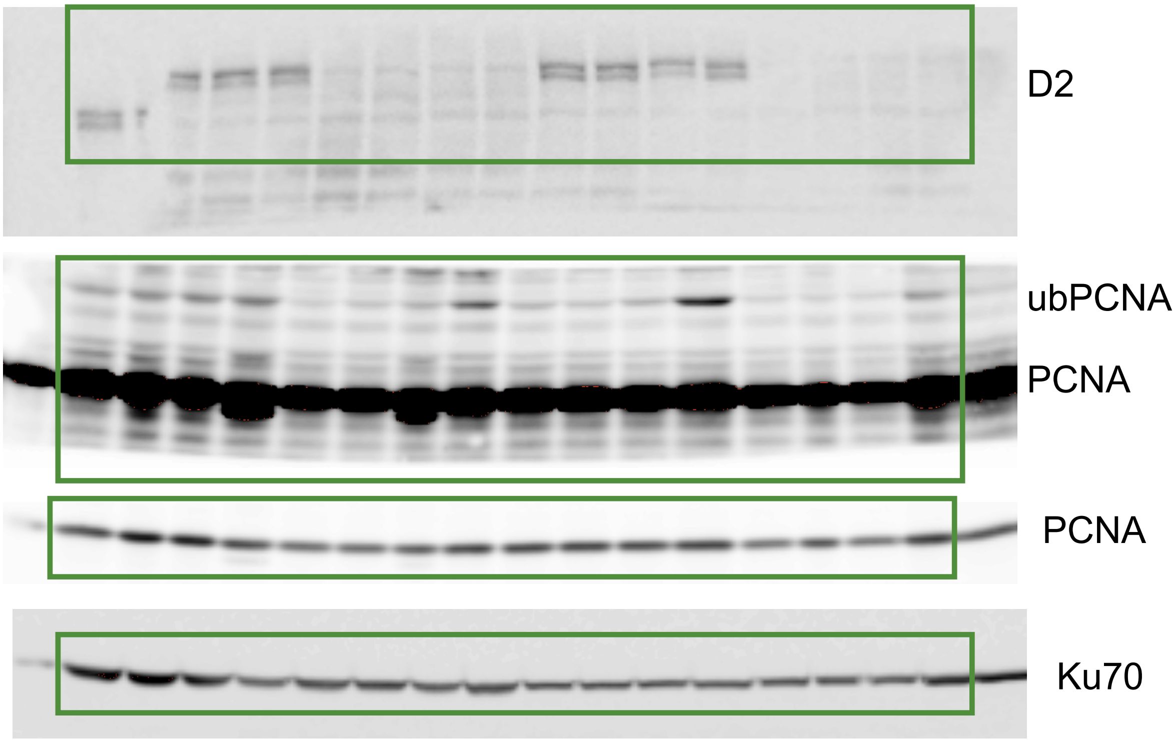

Supplementary Figure 3B

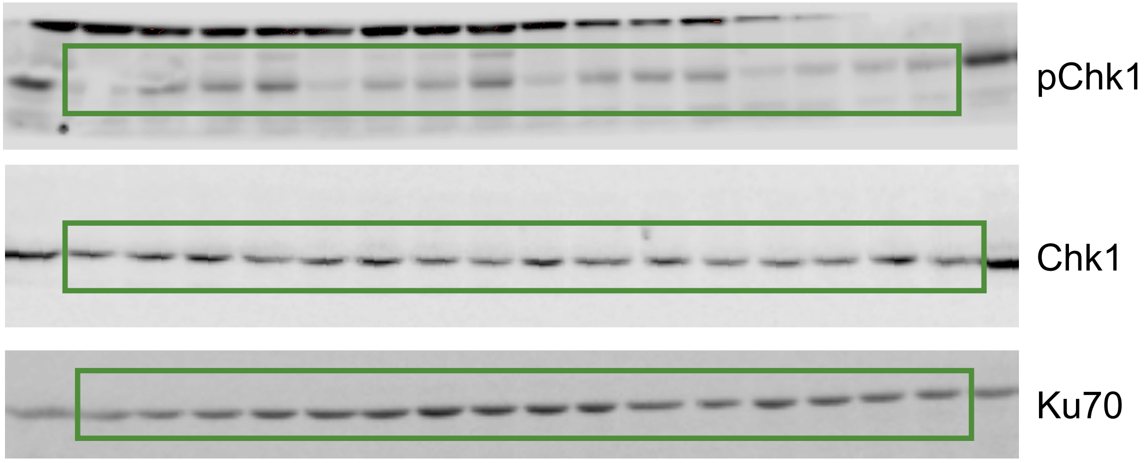

Supplementary Figure 3C

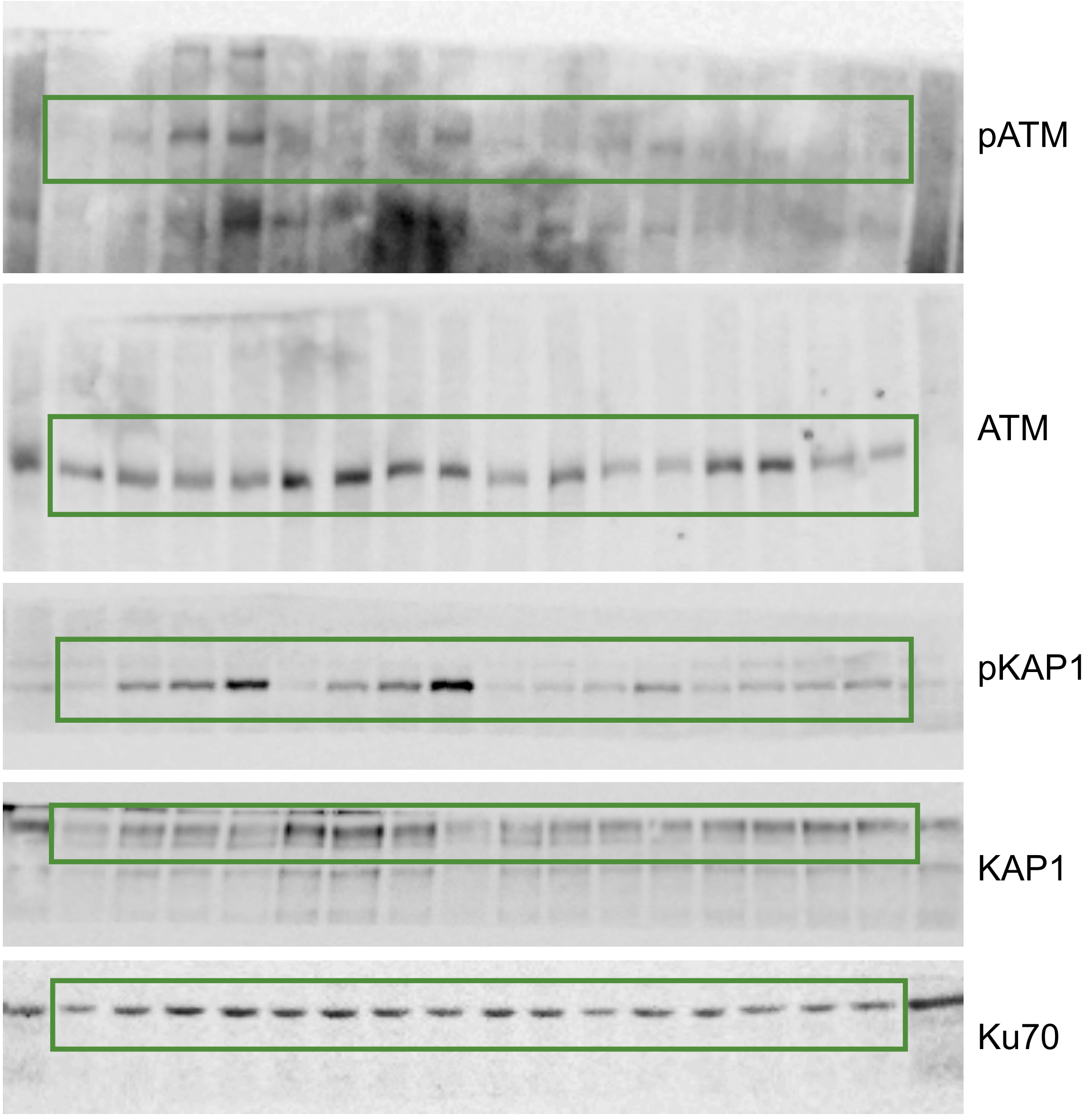

Supplementary Figure 7A

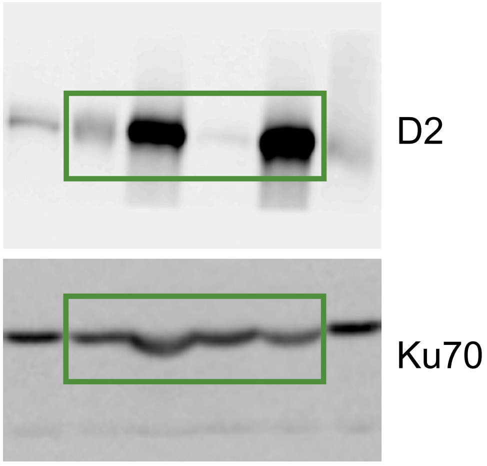

Figure 4 E

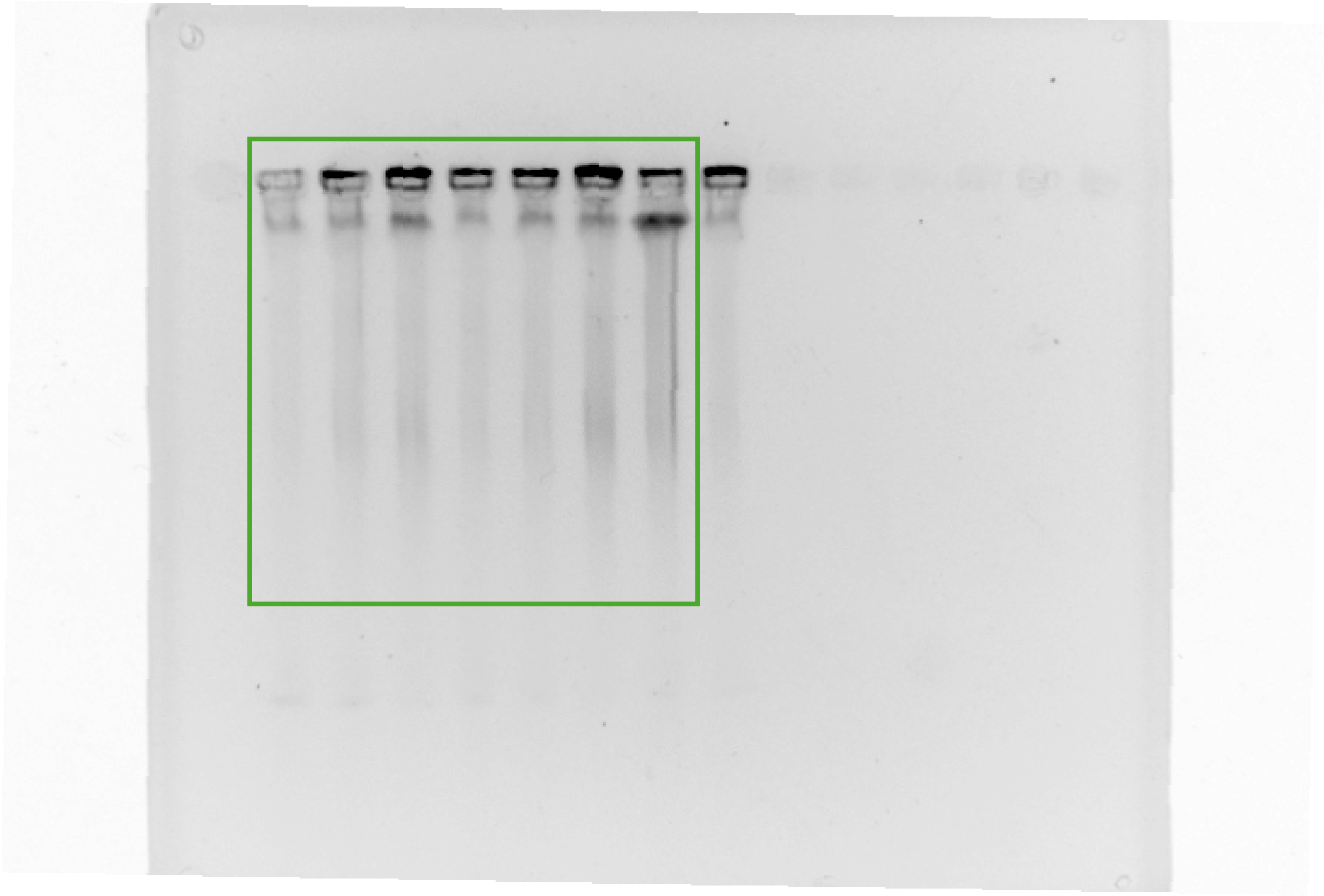

Figure 6A

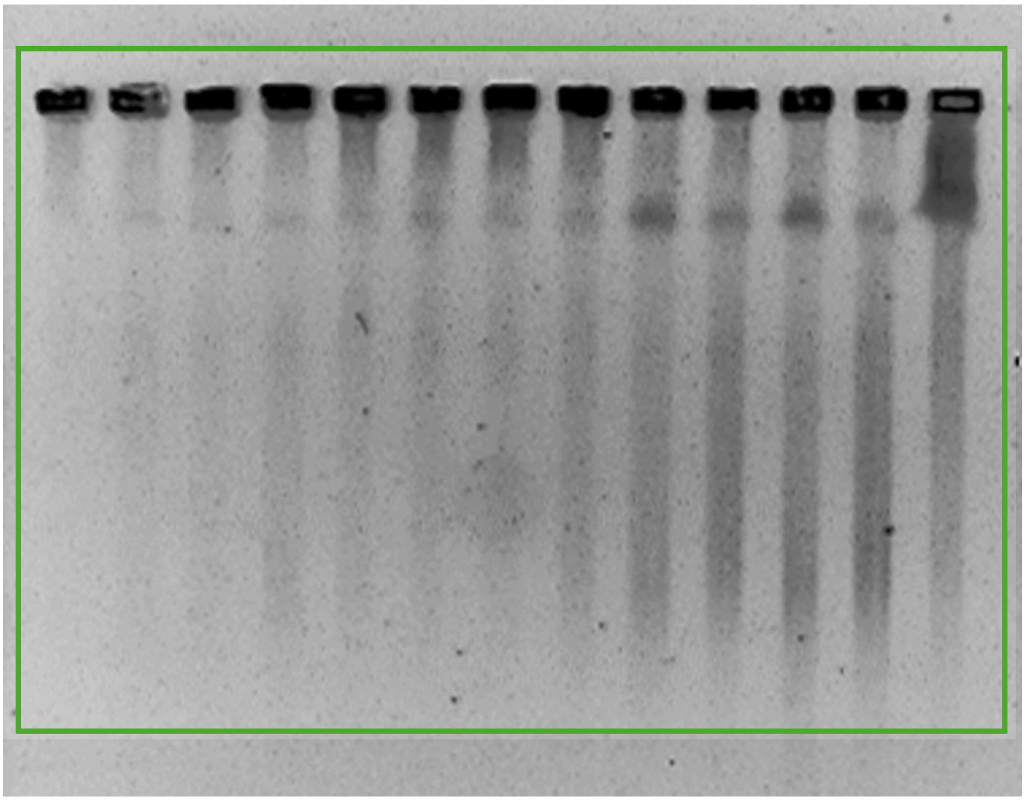

Supplementary Figure 4 D

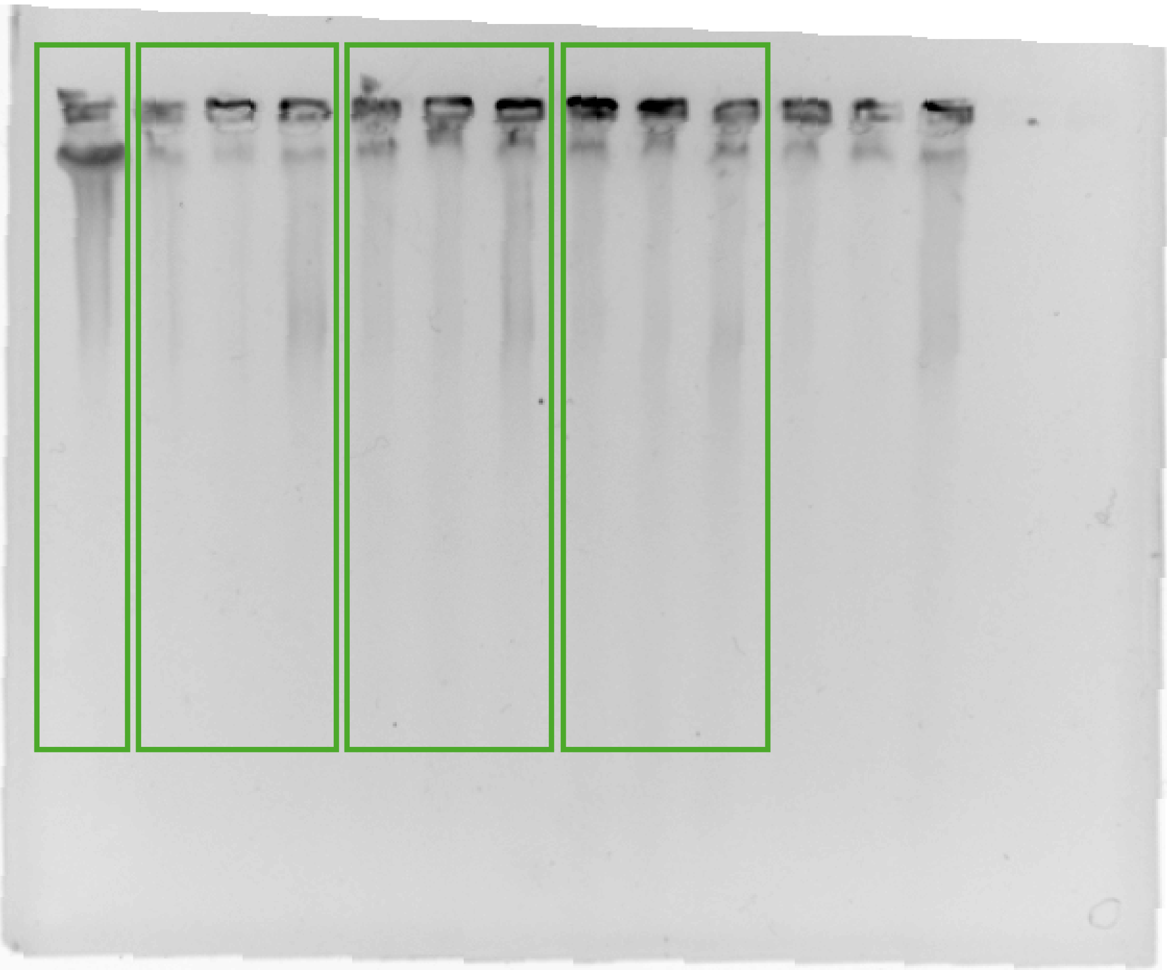

Supplementary Figure 4 E

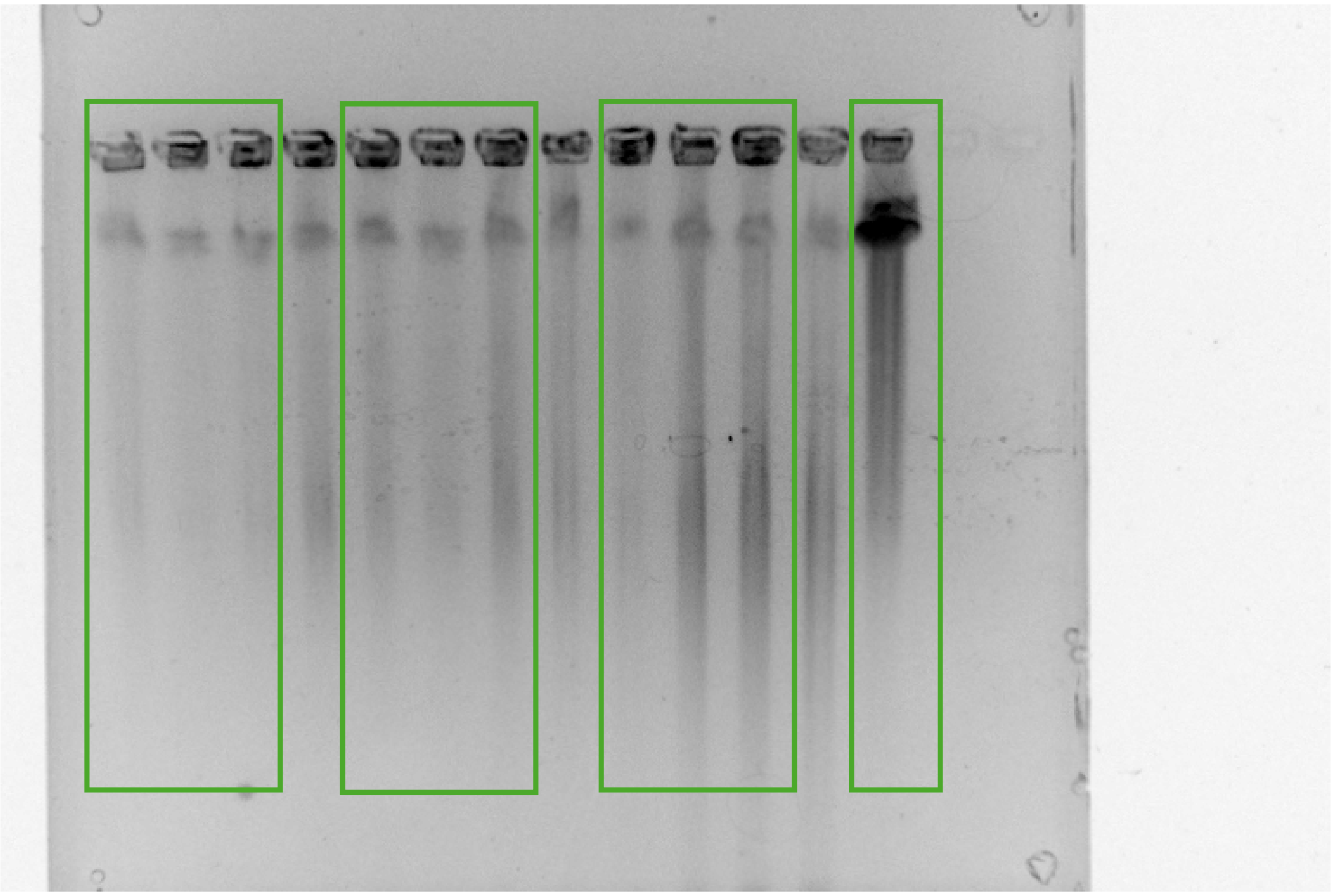

Supplementary Figure 6 C

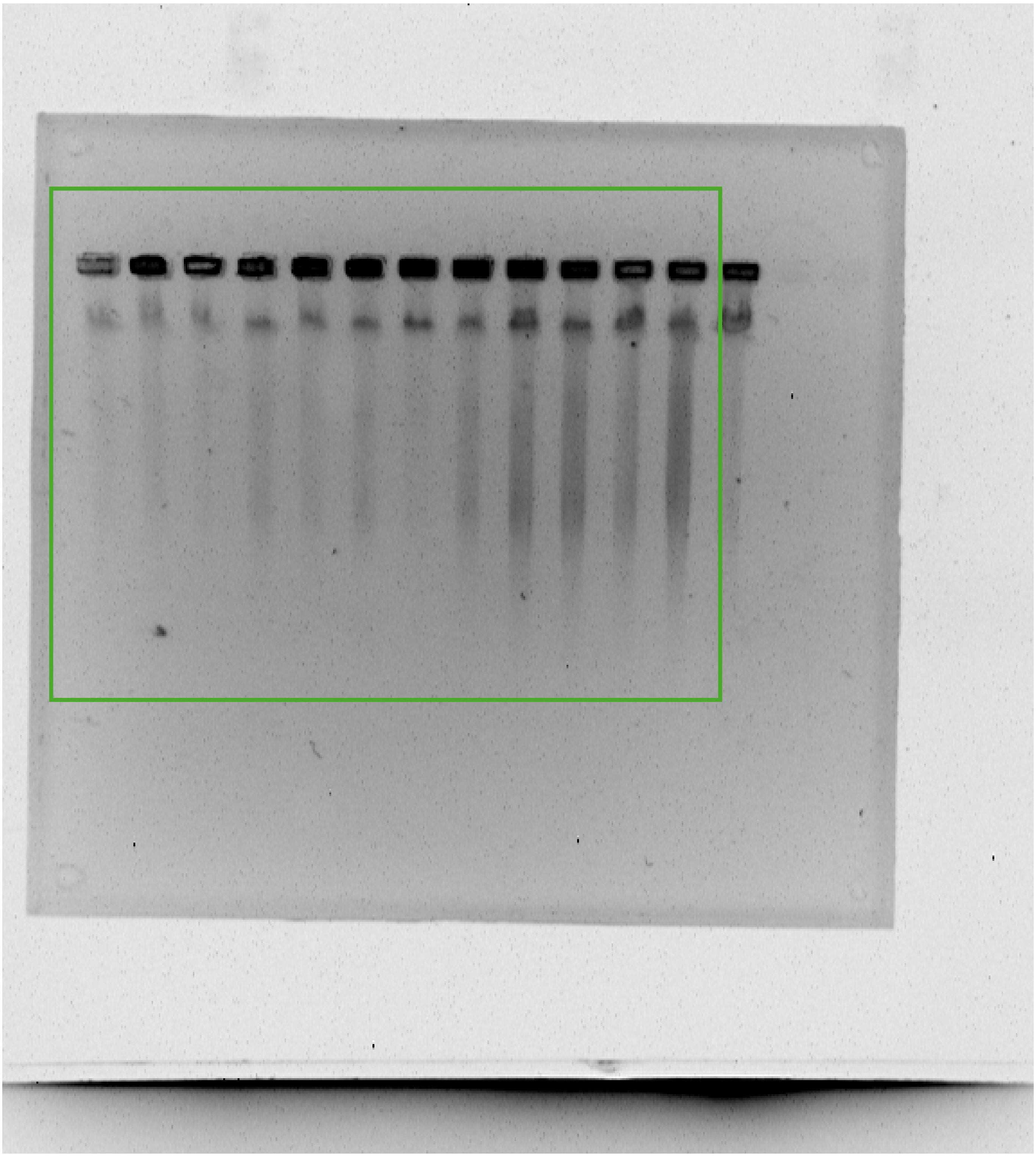

Supplement: S5 File — (A) Annotated original Western blot images, and (B) individual original Western blot images. (ZIP) [file pgen.1011094.s005.zip › S5 File. Original western blot images/S5A File. Annotated original western blots.pdf]
